# Toxicogenomic analysis of exposure to TCDD, PCB126 and PCB153: identification of genomic biomarkers of exposure to AhR ligands

**DOI:** 10.1186/1471-2164-11-583

**Published:** 2010-10-19

**Authors:** Bladimir J Ovando, Corie A Ellison, Chad M Vezina, James R Olson

**Affiliations:** 1 Department of Pharmacology and Toxicology, School of Medicine and Biomedical Sciences, University at Buffalo, Buffalo, New York 14214, USA; 2 School of Pharmacy and Molecular and Environmental Toxicology Center, University of Wisconsin, Madison, Wisconsin 53705, USA

## Abstract

**Background:**

Two year cancer bioassays conducted by the National Toxicology Program have shown chronic exposure to dioxin-like compounds (DLCs) to lead to the development of both neoplastic and non-neoplastic lesions in the hepatic tissue of female Sprague Dawley rats. Most, if not all, of the hepatotoxic effects induced by DLC's are believed to involve the binding and activation of the transcription factor, the aryl hydrocarbon receptor (AhR). Toxicogenomics was implemented to identify genomic responses that may be contributing to the development of hepatotoxicity in rats.

**Results:**

Through comparative analysis of time-course microarray data, unique hepatic gene expression signatures were identified for the DLCs, 2,3,7,8-tetrachlorodibenzo-p-dioxin (TCDD) (100 ng/kg/day) and 3,3',4,4',5-pentachlorobiphenyl (PCB126) (1000 ng/kg/day) and the non-DLC 2,2',4,4',5,5',-hexachlorobiphenyl (PCB153) (1000 μg/kg/day). A common time independent signature of 41 AhR genomic biomarkers was identified which exhibited at least a 2-fold change in expression following subchronic (13-wk) and chronic (52-wk) p.o. exposure to TCDD and PCB126, but not the non DLC, PCB153. Real time qPCR analysis validated that 30 of these genes also exhibited at least a 2-fold change in hepatic expression at 24 hr following a single exposure to TCDD (5 μg/kg, po). Phenotypic anchoring was conducted which identified forty-six genes that were differently expressed both following chronic p.o. exposure to DLCs and in previously reported studies of cholangiocarcinoma or hepatocellular adenoma.

**Conclusions:**

Together these analyses provide a comprehensive description of the genomic responses which occur in rat hepatic tissue with exposure to AhR ligands and will help to isolate those genomic responses which are contributing to the hepatotoxicity observed with exposure to DLCs. In addition, the time independent gene expression signature of the AhR ligands may assist in identifying other agents with the potential to elicit dioxin-like hepatotoxic responses.

## Background

Dioxin-like compounds (DLCs) such as polychlorinated biphenyls (PCBs) and polychlorinated dibenzo-p-dioxins (PCDDs) are prevalent contaminants which pose a risk to both public health and the environment. Exposure to PCBs and PCDDs has been associated with numerous adverse biological effects including reproductive toxicity, dermatotoxicity, immunotoxicity, developmental toxicity, neurotoxicity, carcinogenesis and hepatotoxicity [1-5]. The carcinogenic and hepatotoxic effects of DLCs have been shown to be gender dependent, with female rats being more susceptible than male rats [6]. The DLCs, 2,3,7,8-tetrachlorodibenzo-p-dioxin (TCDD) and 3,3',4,4',5-pentachlorobiphenyl (PCB126); and the non-DLC 2,2',4,4',5,5',-hexachlorobiphenyl (PCB153) were investigated by the National Toxicology Program in a two-year cancer bioassay evaluating their hepatotoxic and carcinogenic properties in female Sprague-Dawley (SD) rats [4,5,7,8]. Following 104 weeks of chronic p.o. exposure to TCDD (100 ng/kg/day) or PCB126 (1000 ng/kg/day), a significant and similar increase in the incidence and range of non-neoplastic and neoplastic lesions were observed in the livers of female rats (Table 1) [4,5]. The non-neoplastic lesions included, but were not exclusive to, hepatocyte hypertrophy, pigmentation, bile duct hyperplasia, oval cell hyperplasia, fatty diffuse change, necrosis, inflammation and cholangiofibrosis. The neoplastic lesions included hepatocellular adenoma and cholangiocarcinoma. A significant increase in the incidence of 6 of these non-neoplastic lesions and no neoplastic lesions were also observed following 52 weeks of exposure to TCDD or PCB126, while only hepatocyte hypertrophy was observed following 13 weeks of exposure (Table 1). Thus, the range of hepatotoxic responses to these DLCs is directly dependent on the duration of exposure. In comparison, chronic exposure (104 weeks) to the non-DLC PCB153 (1000 μg/kg/day) only caused a significant increase in the incidence of two non-neoplastic lesions (hepatocyte hypertrophy and diffuse fatty change) and did not lead to the formation of neoplasia (Table 1) [8].

**Table 1:** Neoplastic and non-neoplastic lesions observed in hepatic tissue of female Sprague-Dawley rats following 104 weeks of chronic p.o. exposure

	100 ng/kg/day TCDD	30 ng/kg/day PCB126	300 ng/kg/day PCB126	1000 ng/kg/day PCB126	1000 μg/kg/day PCB153
Number of Rats Examined	53	55	53	53	53
Hepatocyte Hypertrophy	52**^a^	23**	42**^a^	49**^a^	39**^a^
Multinucleated Hepatocytes	51**^b^	2	19**	49**^b^	NA
Fatty Change, Diffuse	48**^b^	7	30**	47**^b^	21**
Bile Duct Hyperplasia	53**^b^	7	14**	45**^b^	10
Bile Duct Cyst	21**	6	3	12*	NA
Oval Cell, Hyperplasia	53**	1	10**	40**	NA
Necrosis	17**	2	11	17**	NA
Pigmentation	53**^b^	11**	48**^b^	48**^b^	5
Eosiniphilic Focus	44**	10	17	17	NA
Inflammation	49**	40	51**	51**	NA
Nodular Hyperplasia	36**	NA	3	39**	1
Portal Fibrosis	27**	NA	2	10**	NA
Cholangiofibrosis	31**	1	3	22**	1
Toxic Hepatopathy	53**^b^	6*	39**	49**^b^	NA
Hepatocholangioma	17**	NA	NA	3	2
Hepatocellular Adenoma	13**	2	2	7*	NA
Cholangiocarcinoma	25**	NA	5*	22**	NA

Shown are the number of animals in each treatment group which exhibit the indicated liver pathology following exposure to TCDD (100 ng/kg/day), PCB126 (30, 300 or 1000 ng/kg/day) or PCB153 (1000 μg/kg/day). Data acquired from the National Toxicology Program [4,5,8]. ^a ^Response also significantly increased (p ≤ 0.01) following 13 and 52 weeks of exposure; ^b ^Response also significantly increased (p ≤ 0.01) following 52 weeks of exposure; * Statistically significant when compared to control with p-value ≤ 0.05; ** Statistically significant when compared to control with p-value ≤ 0.01; NA - not available.

Most, if not all, of the hepatotoxic effects induced by DLCs are believed to involve the binding and activation of the aryl hydrocarbon receptor (AhR). Ligand activation of the AhR induces changes in gene expression and function which are believed to be the major contributing factor to the development of hepatotoxicity, carcinogenicity and other toxic responses of DLCs. DLC-induced AhR-independent genomic and cellular responses have also been reported [9,10], however, these responses likely do not play a major role in the development of hepatotoxicity induced by DLCs. The importance of the AhR in DLC-induced toxicity has been determined in acute studies conducted with female AhR knockout mice. Toxic effects that were observed in wild type mice but were absent in AhR knockout mice, included wasting syndrome, thymic atrophy, lipid accumulation in hepatocytes (diffuse fatty change) and liver hypertrophy [11,12]. Acute TCDD toxicity is also gender, species and strain specific. Following acute exposure to TCDD, female Sprague Dawley rats exhibit a greater down-regulation in gene expression compared to male rats [13]. Sprague Dawley rats and C57BL/6 mice exhibit different hepatic gene expression profiles following acute TCDD exposure with rat-specific gene responses being associated with lipid metabolism and cell growth while mouse-specific responses are involved in immune function and lipid uptake/metabolism [14]. Long-Evans rats and Han/Wistar rats exhibit a 1000-fold difference in sensitivity to acute TCDD lethality [15] which is attributed to a point mutation in the AhR protein of Han/Wistar rats [16]. This suggests that the acute toxic effects of TCDD are dependent on AhR functionality, gender, species and strain, and suggest that the chronic toxic effects of DLCs are also mediated through persistent AhR activation.

Previous work from our laboratory has surveyed hepatic gene expression in response to AhR ligands and non-ligands following acute and 13 weeks of exposure, which were associated with liver hypertrophy in the absence of other hepatotoxic effects [13,17]. Although these studies have led to a better understanding of the acute and subchronic genomic responses to DLCs, the evaluation of hepatic gene expression following chronic exposure to DLCs is needed to effectively identify genomic factors that may be contributing to the hepatotoxic effects of these toxins which are observed following 52 and 104 weeks of exposure (see Table 1). Building on previous microarray experiments, comparative analysis was conducted between microarray data from subchronic (13 weeks) and chronic (52 weeks) time-points to identify genomic biomarkers that are sustained throughout chronic exposure. Genomic biomarkers that were shared by TCDD and PCB126, but not PCB153, were further analyzed for their acute responsiveness to ascertain a subset of genes which may serve as time-independent genomic biomarkers of exposure to AhR ligands in the female SD rat model. Finally, to relate differential hepatic gene expression to the liver pathology observed with chronic exposure to DLCs, phenotypic anchoring was conducted to associate differentially expressed genes with hepatocellular adenoma and cholangiocarcinoma. Together these analyses will provide a comprehensive description of the genomic responses which occur in rat hepatic tissue with subchronic and chronic exposure to AhR ligands and will help to isolate those genomic responses which are contributing to the hepatotoxicity observed with chronic exposure to DLCs.

## Methods

### Animal Exposures and Procurement of Liver Tissue

Liver tissues were obtained from the National Toxicology Program (NTP) 2-year cancer bioassay investigating the relative carcinogenic potencies of the AhR ligands TCDD and PCB126; and the non-ligand PCB153 [4,5,8]. Female SD rats were exposed 5 days a wk via oral gavage to toxicologically equivalent doses of TCDD (100 ng/kg/day) (Toxic equivalence factor (TEF) = 1.0), PCB126 (30 ng, 300 ng or 1000 ng/kg/day) (TEF = 0.1), PCB153 (1000 μg/kg/day) (TEF = 0.0) or a vehicle control of corn oil:acetone (99:1). Rats were exposed to these compounds for 13 wks (subchronic exposure) or 52 wks (chronic exposure). TEFs were determined using the 2005 TEF recommendations provided by the World Health Organization [18]. Liver tissue was also harvested from female SD rats at 24 hr following a single exposure to TCDD (5 μg/kg, po). This exposure was conducted to identify early responsive genes which were also shown to be differentially expressed (up- or down-) following exposures to DLCs. This acute dose of TCDD has been previously shown to result in hepatic tissue concentrations of dioxin similar to those observed with subchronic and chronic exposure [13]. All procedures were carried out with the approval of the University at Buffalo Institutional Animal Care and Use Committee (PMY14098Y).

### RNA Isolation and Hybridization

The storage and processing of liver samples was described earlier by Vezina *et al*. 2004. Following storage at -80°C, liver tissues were disrupted by homogenization and total RNA was isolated with the Qiagen RNeasy kit (Qiagen Inc., Valencia, CA). RNA integrity was assessed using the Agilent Bioanalyzer 2100 (Agilent Technologies, Palo Alto, CA). High quality RNA was transformed into biotinylated cRNA by the Roswell Park Cancer Institute Gene Expression Facility (Buffalo, NY) and hybridized to RGU34A GeneChips (Affymetrix, Santa Clara, CA) and scanned with the Affymetrix 428 scanner.

### Gene Microarray Data Analysis

Probe-level data from cell intensity files were background subtracted and normalized by the gc-Robust Multiarray Analysis (gcRMA) method using ArrayAssist^® ^(Stratagene, CA). Absolute fold changes and t-test statistics (including Benjamini-Hochberg false discovery rate (FDR) corrections) were calculated using ArrayAssist^®^. Probe-sets were filtered to identify those genes which exhibited a change in expression (up- or down-) of at least 2-fold and a t-test p-value ≤ 0.05 between treated (n = 3) and control (n = 3) groups. Comparative analysis was conducted using Microsoft excel (Microsoft Corporation, Redmond, WA) to further filter the data and identify genes that exhibited statistically significant change (≥ 2-fold) with two or more toxicants. Gene annotation and gene symbols were obtained through the Affymetrix NetAffx™ Analysis Center Software. Heat maps were constructed using TIGR Microarray Experiment Viewer 4.0 [19]. Student t-tests and ANOVA analysis with the post-hoc Tukey test were conducted between treatment groups using Minitab™ (Minitab Inc., State College, PA). A complete summary of gene microarray data is available through the Gene Expression Omnibus at the National Center for Biotechnology Information at http://www.ncbi.nlm.nih.gov/geo/ as accession numbers GSE5789 (13 week microarray data) and GSE22263 (52 week microarray data).

### Quantitative Real-time PCR analysis

Quantitative real-time polymerase chain reaction (qPCR) validated the hepatic expression of AhR genomic biomarkers in livers from rats at 24 hr following exposure to TCDD (5 μg/kg, po). Primers were selected from Entrez Gene rat gene reference sequences using Primer3 software [20]. The parameters for primer selection were described previously [17] and primer sequences are listed in additional file 1. Real-time qPCR was conducted on hepatic cDNA using the IQ SYBR green supermix kit (Bio-Rad Laboratories, Hercules, CA) as described previously in Ovando *et al*. (2006). Statistical comparisons of control vs. treated groups was performed with a 2-sample t-test using Minitab 15 statistical software (Minitab Inc., State College, PA)

### Identification of Dioxin Response Elements (DREs)

Gene regulatory regions spanning 5000 bp above and 1000 bp below the transcriptional start site of target genes were obtained from the University of California, Santa Cruz, Genome Browser using Entrez Gene GeneID numbers. All obtained sequences were analyzed for core DRE sequences (5'-GCGTG-3') using MatInspector (Genomatix Software GmbH, Munchen, Germany). Putative DREs were those with a core similarity of 1.0 and matrix similarity equal to or greater than the optimized matrix threshold [21].

### Phenotypic Anchoring of Gene Expression Data

Changes in gene expression associated with TCDD, PCB126 and PCB153 exposure were compared with changes in gene expression associated with rat hepatocellular adenoma (HCA) [22], human HCA [23]and human intrahepatic cholangiocarcinoma (ICC) [24,25]. Eighty-one percent of the human HCA tumors were from males [23] while 52% [24] and 41% [25] of the human ICC tumors were from males. Our approach was limited in that the HCA and ICC expression data was not reported on a gender specific basis thus preventing us from identifying shared gene responses based on gender. Ortholog identification and gene annotation of gene array data obtained from published studies was accomplished using ArrayTrack (Food and Drug Administration, NTCR) and/or NetAffix™ (Affymetrix Inc.) [26].

## Results

### Dose-response Analysis of Hepatic Gene Expression following Chronic Exposure to 30 ng, 300 ng and 1000 ng/kg/day PCB126

Increases in the incidence of non-neoplastic and neoplastic hepatic lesions were observed with increasing dose and duration of exposure to PCB126 (Table 1) [5]. To evaluate the effect of increasing dose of PCB126 on hepatic gene expression, microarray analysis was conducted on hepatic tissue of female SD rats following 52 weeks of chronic exposure to 30 ng, 300 ng and 1000 ng/kg/day PCB126. Gene array analysis showed a positive trend between PCB126 dose and the number of genes differentially expressed (Table 2). In addition, the magnitude of differential expression of several genes also increased with increasing dose of PCB126 (additional files 2, 3 and 4). Sixteen genes were identified which exhibited altered expression at all three doses (Table 3). Four of the sixteen genes were classic AhR responsive genes and exhibited statistically significant increases in expression with increasing dose of PCB126. These genes included *Cyp1a1 *(cytochrome P450 1A1), *Cyp1b1 *(cytochrome P450 1B1), *Ugt1a6 *(UDP glycosyltransferase 1 family, polypeptide A6) and *Ugt1a7 *(UDP glycosyltransferase 1 family, polypeptide A7) (Table 3). The remaining genes in Table 3 represent a novel set of sensitive genomic biomarkers for chronic exposure to PCB126.

**Table 2:** Summary of differentially expressed genes in the livers of female Sprague-Dawley rats following p.o. exposure to TCDD, PCB126 and PCB153 for 13 and 52 weeks

13 Week Subchronic Exposure Experiment	
Exposure Group	Number of Differentially Expressed Genes

100 ng/kg/day TCDD	103 (18)
1000 ng/kg/day PCB126	371 (164)
1000 μg/kg/day PCB153	39 (0)

52 Week Chronic Exposure Experiment	

Exposure Group	Number of Differentially Expressed Genes

100 ng/kg/day TCDD	299 (22)
30 ng/kg/day PCB126	52 (1)
300 ng/kg/day PCB126	128 (15)
1000 ng/kg/day PCB126	216 (171)
1000 μg/kg/day PCB153	47 (1)

Shown are the total number of probe sets exhibiting a ≥ 2-fold change in expression and a p-value < 0.05 following the specified treatments. In parenthesis are probe sets that are still significant following Benjamini-Hochberg false discovery rate correction.

**Table 3:** Relative fold change in 16 genes differentially expressed in livers of female Sprague-Dawley rats following 52 weeks of chronic p.o. exposure to 30 ng, 300 ng and 1000 ng/kg/day PCB126

			PCB126 dose (ng/kg/d)		
			
Transcript ID	Gene Symbol	Gene Name	30	300	1000
NM_012540	Cyp1a1^a^	Cytochrome P450, family 1, subfamily a, polypeptide 1	499*	1263*	1551*
NM_012940	Cyp1b1^a^	Cytochrome P450, family 1, subfamily b, polypeptide 1	18	866*	2091*
NM_173339	Ceacam10^b^	CEA-related cell adhesion molecule 10	17	660*	976*
NM_031530	Ccl2	Chemokine (C-C motif) ligand 2	5	11	31*
NM_001039691///NM_057105	Ugt1a6^a^	UDP glycosyltransferase 1 family, polypeptide A6	3	11*	16*
NM_130407	Ugt1a7^a^	UDP glycosyltransferase 1 family, polypeptide A7	3	23*	40*
NM_017127	Chka	Choline kinase alpha	2	7*	6*
NM_012656	Sparc	Secreted acidic cysteine rich glycoprotein	2	2	4*
NM_031569///NM_057098///XM_001055907///XM_345486	Oprl1///Tcea2	Opioid receptor-like 1///Transcription elongation factor A (SII), 2	2	4	4*
NM_024127	Gadd45a	Growth arrest and DNA-damage-inducible 45 alpha	2	4	4*
NM_012600	Me1	Malic enzyme 1	2	2*	4*
NM_001037979	Adipor2^b^	Adiponectin receptor 2	-3	-2	-6*
NM_012672	Thrb	Thyroid hormone receptor beta	-3	-3	-3*
NM_032071	Synj2	Synaptojanin 2	-3	-3	-4*
XM_001071608///XM_213849	Nfix	Nuclear factor I/X	-3	-3	-5*
NM_012988	Nfia	Nuclear factor I/A	-4	-4	-3*

^a ^Statistically different (p-value = 0.05) between doses using ANOVA with Tukey post-hoc test; ^b ^Statistically different between 300 ng and 1000 ng doses only; * Genes with p-value < 0.05 following Benjamini-Hochberg FDR correction.

### Identifying Genomic Biomarkers of Subchronic and Chronic Exposure to TCDD, PCB126 and PCB153

During the 2-year cancer bioassays conducted by the NTP, it was observed that continuous exposure to DLCs beyond 30 weeks was necessary to lead to the formation of hepatic neoplastic lesions (Table 1). Rats treated with TCDD or PCB126 for 30 weeks, and then with vehicle control for the remainder of the two-year cancer bioassay showed no difference in the incidence of hepatocellular adenoma or cholangiocarcinoma when compared to control animals [4,5]. This suggests that persistent AhR activation with long-term alterations in gene expression are necessary for the development of hepatic neoplasia.

**Figure 1:**
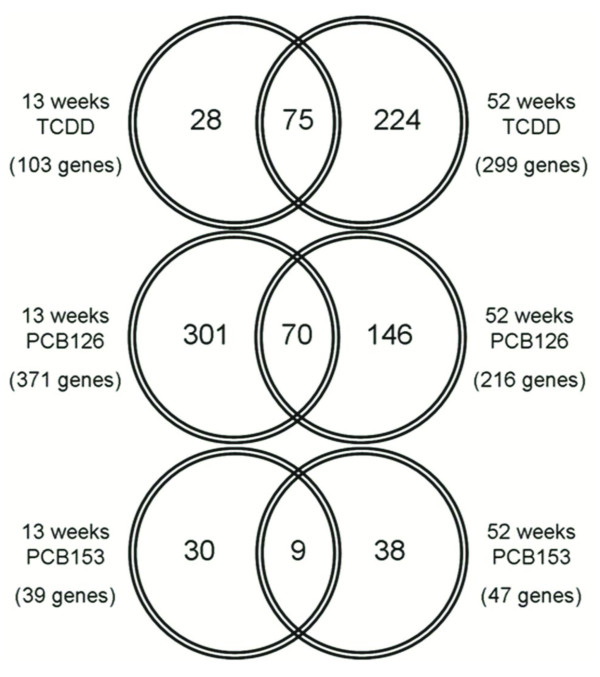
**Comparison of gene expression profiles following 13 and 52 week exposure to TCDD, PCB126 or PCB153**. Venn diagram depicting the number of genes shared by the hepatic gene expression profiles in female Sprague-Dawley rats following 13 weeks and 52 weeks of exposure to 100 ng/kg/day TCDD, 1000 ng/kg/day PCB126 or 1000 μg/kg/day PCB153. In parenthesis is the total number of significantly different genes differentially expressed for each exposure group. Genes were deemed significantly different if they expressed a ≥2-fold change in expression and a p-value ≤ 0.05 as determined by student t-test analysis.

**Figure 2:**
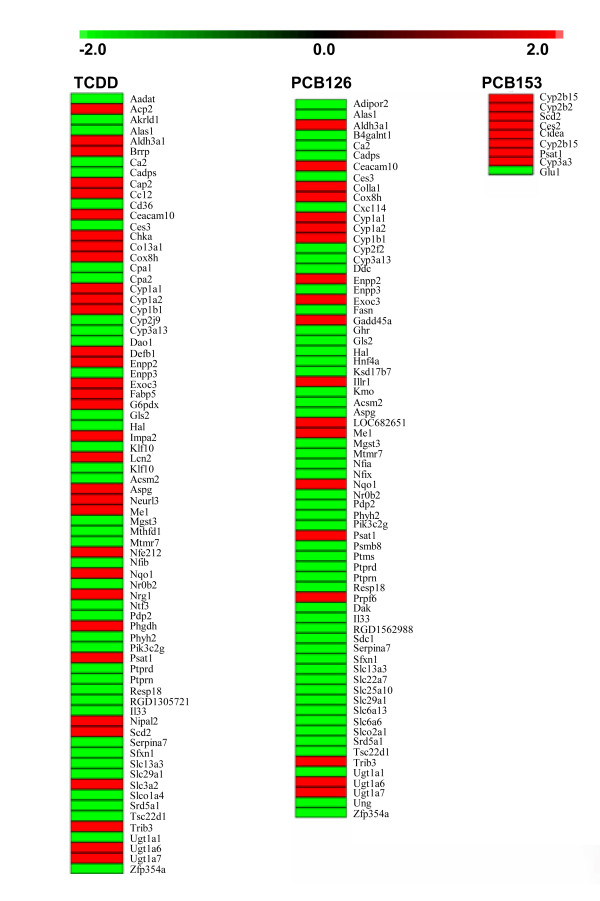
**Heat map of hepatic gene expression signature for TCDD, PCB126 and PCB153**. Heat map depicting hepatic gene expression signatures in female Sprague-Dawley rats exposed to TCDD, PCB126 and PCB153. The expression signature for each compound represents genes differentially expressed (≥2-fold change) following both 13 and 52 weeks of exposure.

To identify genomic responses which are sustained throughout chronic exposure, comparative analysis of time-course microarray data was conducted. Subchronic (13 weeks) and chronic (52 weeks) exposure to TCDD (100 ng/kg/day), led to the differential expression of 103 and 299 genes, respectively (Table 2 and additional files 5 and 6). Through comparative analysis of subchronic and chronic exposure, 75 genes were identified that exhibited the same differential expression pattern at both time points (Figures 1 and 2). Following a similar paradigm for exposure to PCB126 (1000 ng/kg/day), 70 genes were identified that sustained the same differential expression pattern at both time points (Figures 1 and 2 and additional files 4 and 7). The non-hepatotoxic PCB153 (Table 1) caused the sustained differential expression of only 9 genes following subchronic and chronic exposure (Figures 1 and 2 and additional files 8 and 9). The sustained genomic responses to TCDD, PCB126 and PCB153 serve as genomic biomarkers of subchronic and chronic exposure to these compounds.

### Identification of Genomic Biomarkers of Exposure to AhR Ligands

**Figure 3:**
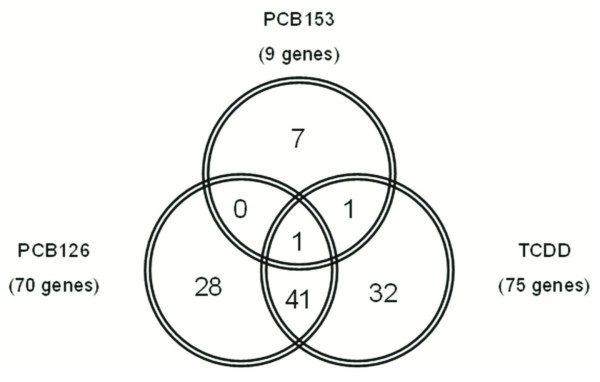
**Comparison of hepatic gene expression signatures for TCDD, PCB126 and PCB153**. Venn diagram depicting the number of genes shared by the hepatic gene expressions signatures in female Sprague-Dawley rats following subchronic and chronic exposure to TCDD (100 ng/kg/day), PCB126 (1000 ng/kg/day) or PCB153 (1000 μg/kg/day). In parenthesis is the total number of genes differentially expressed for each compound following both 13 and 52 weeks of exposure. The 41 genes shared in common by exposures to TCDD and PCB126 represent genomic biomarkers of subchronic and chronic exposure to AhR ligands or the time independent gene expression signature of the AhR ligands.

The hepatic gene expression signatures of TCDD and PCB126, while not identical, did exhibit a great deal of overlap. Genes that are shared by both expression signatures represent genomic biomarkers of subchronic and chronic exposure to two AhR ligands at toxic equivalent doses. Forty-one genomic biomarkers were identified that were shared by the expression signatures of TCDD and PCB126, but not PCB153 (Table 4 and Figure 3). These 41 genes are genomic biomarkers of exposure to two different AhR ligands and may be applicable to other AhR ligands as biomarkers of exposure (Table 4). Only one gene, *Psat1 *(phosphoserine aminotransferase 1), was found to be shared by the expression signatures of all three compounds (Figures 2 and 3), where its expression was up-regulated 3- to 8-fold following exposure to TCDD, PCB126, and PCB153.

**Table 4:** Relative fold change in genes differentially expressed in livers of female Sprague-Dawley rats following 13 weeks and 52 weeks of p.o. exposure to 1000 ng/kg/day PCB126 and 100 ng/kg/day TCDD, and 24 h post-exposure to 5 μg/kg TCDD, p.o.

Gene Symbol	Gene Name	13 wk PCB126^a^	13 wk TCDD^a^	52 wk PCB126^a^	52 wk TCDD^a^	24 h TCDD^b^	DRE site in Rat^c^	DRE site in Human^c^
Cyp1a1	Cytochrome P450, family 1, subfamily a, polypeptide 1	1140*	1026*	1551*	1277*	127881**	YES (8)	YES (11)
Cyp1b1	Cytochrome P450, family 1, subfamily b, polypeptide 1	519*	743*	2091*	1288*	121^d^**	YES (9)	YES (7)
Ceacam10	CEA-related cell adhesion molecule 10	369*	620*	976*	973*	8.5**	NO	N/P
Cox8h	Cytochrome c oxidase subunit VIII-H (heart/muscle)	66*	312*	24*	27*	N/D	NO	N/A
Nqo1	NAD(P)H dehydrogenase, quinone 1	21*	20	9*	8*	37.0**	YES (7)	YES (2)
Ugt1a7	UDP glycosyltransferase 1 family, polypeptide A7	14*	85*	40*	33*	24.5**	YES (4)	YES (2)
Ugt1a6	UDP glycosyltransferase 1 family, polypeptide A6	11*	56*	16*	12*	383.6**	YES (6)	YES (2)
Cyp1a2	Cytochrome P450, family 1, subfamily a, polypeptide 2	7*	3*	2*	2	38.5**	NO	YES (3)
Enpp2	Ectonucleotide pyrophosphatase/phosphodiesterase 2	6*	6*	5*	4	2.6**	YES (1)	NO
Exoc3	Exocyst complex component 3	6*	32*	21*	23*	8.1**	NO	YES (7)
Aldh3a1	Aldehyde dehydrogenase family 3, member A1	5*	57*	1493*	1156*	10.2**	YES (2)	YES (5)
Me1	Malic enzyme 1	4*	3	4*	3	2.4**	YES (2)	YES (2)
Trib3	Tribbles homolog 3 (Drosophila)	2*	8*	13*	16	2.2**	YES (1)	YES (3)
Tsc22d1	TSC22 domain family, member 1	-2	-2	-6*	-4	1.5	YES (4)	YES (2)
Gls2	Glutaminase 2 (liver, mitochondrial)	-2*	-3	-6*	-4	-2.5**	YES (2)	YES (3)
Zfp354a	Zinc finger protein 354A	-2	-2	-8*	-5	-5.3	YES (1)	YES (1)
Alas1	Aminolevulinic acid synthase 1	-2*	-2	-3	-3	-1.6	N/A	YES (2)
Phyh2	Phytanoyl-CoA 2-hydroxylase 2	-2	-4	-3*	-3	-1.6	YES (1)	YES (1)
Ca2	Carbonic anhydrase 2	-3	-8*	-6*	-3	-1.9^d^	NO	YES (4)
Sfxn1	Sideroflexin 1	-3*	-3*	-3*	-2	-2.9	NO	YES (2)
Mtmr7	Myotubularin related protein 7	-3*	-3	-5*	-5	-3.0**	N/A	YES (2)
Pik3c2g	Phosphatidylinositol 3-kinase, C2 domain containing, gamma polypeptide	-3	-2	-3*	-3	-1.5	NO	NO
Acsm2	Acyl-CoA synthetase medium-chain family member 2	-3	-26	-12*	-14	-3.9	YES (2)	N/P
Slc29a1	Solute carrier family 29 (nucleoside transporters), member 1	-3*	-3	-3*	-2*	-2.1	YES (2)	YES (1)
Cadps	Ca2+-dependent secretion activator	-3	-3	-3	-3	-2.6**	YES (1)	YES (5)
Hal	Histidine ammonia lyase	-3	-2	-17*	-10	-1.3	YES (2)	NO
Cyp3a13	Cytochrome P450, family 3, subfamily a, polypeptide 13	-3	-139*	-610*	-606*	-5.6^d^**	NO	N/P
Ptprd	Protein tyrosine phosphatase, receptor type, D	-3*	-2	-3	-2	-1.9	YES (4)	YES (2)
Ces3	Carboxylesterase 3	-3*	-9	-9*	-6	-2.8^d^	NO	YES (2)
Mgst3	Microsomal glutathione S-transferase 3	-3	-3	-4*	-4	-3.4**	YES (7)	YES (4)
Serpina7	Serine (or cysteine) peptidase inhibitor, clade A (alpha-1 antipeptidase, antitrypsin), member 7	-3	-15	-21*	-16	-3.3^d^	NO	NO
Nr0b2	Nuclear receptor subfamily 0, group B, member 2	-3*	-2	-3*	-2	-6.4**	NO	YES (2)
Aspg	Asparaginase homolog (S. cerevisiae)	-4*	-10	-41*	-28	-4.1**	YES (8)	N/P
Il33	Il33 interleukin 33	-4*	-3	-3*	-3	-2.1**	NO	N/P
Ptprn	Protein tyrosine phosphatase, receptor type, N	-4	-3	-4*	-3	-1	YES (2)	YES (1)
Srd5a1	Steroid 5 alpha-reductase 1	-4	-6	-14*	-11	-2.0^d^**	YES (1)	YES (8)
Enpp3	Ectonucleotide pyrophosphatase/phosphodiesterase 3	-4*	-4*	-5*	-4	-2.3**	NO	NO
Pdp2	Pyruvate dehydrogenase phosphatase isoenzyme 2	-4	-3	-9*	-3	-1.8	YES (1)	YES (3)
Ugt1a1	UDP glycosyltransferase 1 family, polypeptide A1	-5*	-3	-3*	-3	-1.3	YES (4)	YES (4)
Slc13a3	Solute carrier family 13 (sodium-dependent dicarboxylate transporter), member 3	-5	-7	-4*	-5	-3.3^d^**	YES (3)	YES (1)
Resp18	Regulated endocrine-specific protein 18	-7	-6	-15*	-34	-5.7**	NO	N/A

Shown are genes for which expression from microarray analysis significantly differed (p < 0.05 as determined by the student t-test), by at least 2-fold, compared to the control group. ^a ^Fold changes in hepatic gene expression from microarray analysis; ^b ^Fold changes in hepatic gene expression from qPCR at 24 h post-exposure to TCDD; ^c ^DREs identified in genomic DNA sequences spanning 5000 bp above and 1000 bp below the transcriptional start site. The number in parenthesis indicates the number of DREs present; ^d ^Values from Ovando et al. 2006; *Genes with p-value < 0.05 following Benjamini-Hochberg FDR correction; **Statistically different (P ≤ 0.05) between control and treated animals (n = 3) for qPCR; N/P - Gene not present in human genome; N/D - Gene expression could not be evaluated for that treatment; N/A - Not available in the UCSC genome bioinformatics database.

The sustained differential expression of these 41 AhR genomic biomarkers at both subchronic and chronic time-points suggests that these genomic responses are time-independent. To validate these biomarkers and determine if the differential expression of these genomic biomarkers are time-independent, real-time qPCR was utilized to evaluate hepatic gene expression in female SD rats at 24 h post-exposure to an acute dose of TCDD (5 μg/kg, po.) (Table 4). Thirty of these 41 genes exhibited a 2-fold or greater change in expression 24 h post-exposure to TCDD (5 μg/kg). While acute exposure to TCDD resulted in less than a 2-fold change in the hepatic expression of 10 AhR genomic biomarkers, 9 of the 10 genes exhibited a similar trend in the up or down regulation observed following subchronic and chronic exposure to TCDD and PCB126 (Table 4). The 30 genes confirmed through real-time qPCR to be up- or down-regulated (≥ 2-fold change in expression) represent time-independent genomic biomarkers of AhR ligands that are responsive at acute, subchronic and chronic time-points and may be applied as a diagnostic and mechanistic tool for the evaluation of AhR-ligand like activity in the female SD rat model.

The 41 AhR genomic biomarkers were analyzed for the presence of putative DRE sites (5'-GCGTG-3') within 5000 bp above and 1000 bp below the transcriptional start site. The gene regulatory sequences were obtained for 39 of the 41 genes. Sequences for *Alas1 *(aminolevulinate, delta-, synthase 1) and *Mtmr7 *(myotubularin related protein 7) were not available through the UCSC genome bioinformatic database and thus prevented analysis for DRE sites. Of the 39 genes assessed, 25 of the genes contained one or more putative DRE site (Table 4). This included genes which have been previously shown to be regulated by AhR ligands including the cytochrome P450 genes *Cyp1a1 *and *Cyp1b1 *[10] the UDP glycotransferase genes *Ugt1a6 *and *Ugt1a7 *[27], *Hal *(histidine ammonia lyase) [28], *Nqo1 *(NAD(P)H dehydrogenase, quinone 1) [14], *Srd5a1 *(steroid-5-alpha-reductase, alpha polypeptide) [29] and Tsc22d1 (TSC22 domain family, member 1; also known as *Tgfb1i4) *[30].

### Phenotypic Anchoring of 52-week Hepatic Gene Expression to Hepatocellular Adenoma and Cholangiocarcinoma

Following chronic exposure to TCDD and PCB126 a significant increase in the incidence of HCA and cholangiocarcinoma (CC) is observed in the livers of female SD rats (Table 1). The appearance of HCA and CC was observed with 104 weeks of exposure to TCDD and PCB126, but not at earlier time points or with PCB153. To relate genomic responses to the observed liver pathology, comparative analysis was conducted between the 52 week hepatic gene expression profiles of TCDD, PCB126 and PCB153, and gene array data from published studies on human ICC [24], human HCA [23] and rat HCA [22].

Human ICC gene expression profiles were obtained from microarray studies conducted on 13 [25] and 25 [24] microdissected cholangiocarcinomas. Between the two studies, 24 genes were identified as exhibiting the same differential expression pattern in human ICC and in rat liver following 52 weeks of chronic exposure to TCDD and/or PCB126, but not PCB153 (Table 5). Among these genes, 4 were unique to the Obama *et al*. (2005) ICC expression profile, 18 were unique to the Miller *et al*. (2009) ICC expression profile and 2 genes were shared between the two ICC expression profiles. The two genes that were present in both ICC expression profiles were *Gata6 *(GATA binding protein 6) and *Timp3 *(tissue inhibitor of metalloproteinase 3).

**Table 5:** Relative fold change in genes differentially expressed in human intrahepatic cholangiocarcinoma (ICC) and the hepatic gene expression profiles of rats exposed to TCDD, PCB126, and PCB153 p.o. for 52 weeks

Transcript ID	Rat Ortholog	Rat Gene Name	Human Ortholog	**ICC **[25]	**ICC **[24]	TCDD 100 ng/kg/day	PCB126 1000 ng/kg/day	PCB153 1000 μg/kg/day
NM_144755	Trib3	Tribbles homolog 3 (Drosophila)	TRIB3	18	N/C	16	13	N/C
NM_016988	Acp2	Acid phosphatase 2, lysosomal	ACP2	2	N/C	3	2	N/C
NM_021261	Tmsb10	Thymosin, beta 10	TMSB10	2	N/C	2	N/C	N/C
NM_017059	Bax	Bcl2-associated X protein	BAX	4	N/C	2	N/C	N/C
NM_019287	Apob	Apolipoprotein B	APOB	N/C	-3	-9	N/C	N/C
NM_019185	Gata6	GATA binding protein 6	GATA6	-4	-5	-3	-4	N/C
NM_013219	Cadps	Ca2+-dependent secretion activator	CADPS	N/C	-16	-3	-3	N/C
NM_012886	Timp3	Tissue inhibitor of metalloproteinase 3 (Sorsby fundus dystrophy, pseudoinflammatory)	TIMP3	-11	-3	-2	N/C	N/C
NM_017158	Cyp2c7	Cytochrome P450, family 2, subfamily c, polypeptide 7	CYP2C9	N/C	-3	-2	-2	N/C
NM_134432	Agt	Angiotensinogen (serpin peptidase inhibitor, clade A, member 8)	AGT	N/C	-6	-2	N/C	N/C
NM_053598	Nudt4	Nudix (nucleoside diphosphate linked moiety X)-type motif 4	NUDT4	-3	N/C	-2	N/C	N/C
NM_013029	St8sia3	ST8 alpha-N-acetyl-neuraminide alpha-2,8-sialyltransferase 3	ST8SIA3	-2	N/C	-2	N/C	N/C
NM_012596	Lepr	Leptin receptor	LEPR	-8	N/C	-2	N/C	N/C
NM_022501	Crip2	Cysteine-rich protein 2	CRIP	-9	N/C	-3	-3	N/C
NM_031132	Tgfbr2	Transforming growth factor, beta receptor II	TGFBR2	-4	N/C	-3	N/C	N/C
NM_001025271	Sfpq	Splicing factor proline/glutamine rich (polypyrimidine tract binding protein associated)	SFPQ	-5	N/C	-3	N/C	N/C
NM_001007235	Itpr1	Inositol 1,4,5-triphosphate receptor 1	ITPR1	-3	N/C	-3	-3	N/C
NM_053328	Bhlhb2	Basic helix-loop-helix domain containing, class B2	BHLHB2	-4	N/C	-4	N/C	N/C
NM_001013137	Cxcl14	Chemokine (C-X-C motif) ligand 14	CXCL14	-11	N/C	-4	-10	N/C
NM_031561	Cd36	Cd36 antigen	CD36	-5	N/C	-6	-4	N/C
NM_031135	Klf10	Kruppel-like factor 10	KLF10	-4	N/C	-10	N/C	N/C
NM_022251	Enpep	Glutamyl aminopeptidase	ENPEP	-2	N/C	-12	-5	N/C
NM_031648	Fxyd1	FXYD domain-containing ion transport regulator 1	FXYD1	-14	N/C	N/C	-2	N/C
NM_053698	Cited2	Cbp/p300-interacting transactivator, with Glu/Asp-rich carboxy-terminal domain, 2	CITED2	-4	N/C	N/C	-4	N/C

N/C indicates no change in gene expression for that treatment.

Human and Sprague-Dawley rat HCA gene expression profiles obtained from microdissected HCA tissues [22] were used for comparative analysis. Additional rat HCA gene expression profiles were kindly provided by Dr. Sun Hee Yim (National Cancer Institute, Bethesda, MD). Seventeen genes were identified which exhibited the same differential expression pattern in human HCA as that seen in the livers of rats exposed for 52 weeks to TCDD and/or PCB126 (Table 6). Additionally, seven genes were identified which exhibited the same differential expression pattern in both rat HCA gene expression studies and in livers from rats exposed for 52 weeks to TCDD and/or PCB126, but not PCB153 (Table 6). Interestingly, the genes *Gata6*, *Agt *(angiotensinogen (serpin peptidase inhibitor, clade A, member 8)) and *Bhlhb2 *(basic helix-loop-helix domain containing, class B2) were down-regulated in ICC and HCA as well as the 52 week expression profiles of TCDD and/or PCB126, but not PCB153.

**Table 6:** Relative fold change in genes differentially expressed in human hepatocellular adenoma (HCA), rat HCA and the hepatic gene expression profiles of rats exposed to TCDD, PCB126, and PCB153 p.o. for 52 weeks

Transcript ID	Gene Symbol	Rat Gene Name	Human Ortholog	**HCA **[23]	**HCA **[22]	TCDD 100 ng/kg/day	PCB126 1000 ng/kg/day	PCB153 1000 μg/kg/day
NM_053401	Ngfrap1	Nerve growth factor receptor (TNFRSF16) associated protein 1	NGFRAP1	N/C	13	8	6	N/C
NM_012752	Cd24	CD24 antigen	CD24	10	N/C	7	4	N/C
NM_017127	Chka	Choline kinase alpha	CHKA	2	N/C	5	6	N/C
NM_057104	Enpp2	Ectonucleotide pyrophosphatase/phosphodiesterase 2	ENPP2	4	N/C	4	5	N/C
NM_017166	Stmn1	Stathmin 1	STMN1	2	N/C	4	N/C	2
NM_145878	Fabp5	Fatty acid binding protein 5, epidermal	FABP5	2	N/C	4	3	N/C
NM_022298	Tuba1	Tubulin, alpha 1	TUBA4A	N/C	11	3	N/C	N/C
NM_053515	Slc25a4	Solute carrier family 25 (mitochondrial carrier; adenine nucleotide translocator), member 4	SLC25A4	N/C	8	3	N/C	N/C
NM_012862	Mgp	Matrix Gla protein	MGP	4	N/C	3	3	N/C
NM_012946	Sparcl1	SPARC-like 1 (mast9, hevin)	SPARCL1	3	N/C	2	2	N/C
NM_030987	Gnb1	Guanine nucleotide binding protein, beta 1	GNB1	2	N/C	N/C	3	N/C
NM_031795	Ugcg	UDP-glucose ceramide glucosyltransferase	UGCG	2	N/C	N/C	N/C	2
NM_023103	Mug1	Murinoglobulin 1 homolog (mouse)	none	N/C	-5	-2	N/C	N/C
NM_134432	Agt	Angiotensinogen (serpin peptidase inhibitor, clade A, member 8)	AGT	N/C	-5	-2	N/C	N/C
NM_012899	Alad	Aminolevulinate, delta-, dehydratase	ALAD	-2	N/C	-2	-3	N/C
NM_053770	Argbp2	Arg/Abl-interacting protein ArgBP2	ARGBP2	-3	N/C	-2	N/C	N/C
NM_022508	Mthfd1	Methylenetetrahydrofolate dehydrogenase (NADP+ dependent), methenyltetrahydrofolate cyclohydrolase, formyltetrahydrofolate synthase	MTHFD1	-5	N/C	-2	N/C	N/C
NM_012716	Slc16a1	Solute carrier family 16 (monocarboxylic acid transporters), member 1	SLC16A1	-3	N/C	-2	N/C	N/C
NM_013177	Got2	Glutamate oxaloacetate transaminase 2, mitochondrial	GOT2	-2	N/C	N/C	N/C	-2
NM_012737	Apoa4	Apolipoprotein A-IV	APOA4	N/C	-9	-3	-4	N/C
NM_053923	Pik3c2g	Phosphatidylinositol 3-kinase, C2 domain containing, gamma polypeptide	PIK3C2G	-3	N/C	-3	-3	N/C
NM_024484	Alas1	Aminolevulinic acid synthase 1	ALAS1	-3	N/C	-3	-3	2
NM_019185	Gata6	GATA binding protein 6	GATA6	-3	N/C	-3	-4	N/C
NM_053328	Bhlhb2	Basic helix-loop-helix domain containing, class B2	BHLHB2	-2	N/C	-4	N/C	N/C
XM_001076124///XM_001076147///XM_001076171///XM_341825	Prodh2	Proline dehydrogenase (oxidase) 2	PRODH2	-3	N/C	N/C	-3	N/C
NM_147206	Cyp3a13	Cytochrome P450, family 3, subfamily a, polypeptide 13	CYP3A4	N/C	-9	-606	-610	N/C

N/C indicates no change in gene expression for that treatment.

## Discussion

Toxicological studies conducted by the National Toxicology Program have shown a significant increase in the incidence of hepatic neoplastic and non-neoplastic lesions in female SD rats following chronic exposure to TCDD and PCB126 [4,5,7]. Studies with AhR knockout mice have shown that the acute toxicity of TCDD is dependent on the functionality of the AhR [11,12]. This suggests that the hepatotoxic effects of TCDD and related dioxin-like compounds (DLCs) are mediated through the AhR, and changes in gene expression resulting from activation of this transcription factor are likely the principle mode of toxicity of these compounds. In an effort to identify the genomic responses that may be contributing to the observed liver toxicity, toxicogenomics was conducted to provide a comprehensive description of hepatic gene expression with acute exposure to TCDD and subchronic and chronic exposure to TCDD and PCB126, the most potent dioxin-like PCB.

Through the comparative analysis of time-course microarray data, hepatic gene expression signatures of subchronic and chronic exposure to TCDD, PCB126 and PCB153 were identified (Figures 1 &2). The hepatic gene expression signature of PCB126 (1000 ng/kg/day) consists of 70 genes which show sustained differential expression at both subchronic (13 weeks) and chronic (52 weeks) time points (Figure 1). In addition, a dose response analysis of hepatic gene expression was conducted following 52 weeks of chronic exposure to 30 ng, 300 ng and 1000 ng/kg/day PCB126. Gene array analysis showed a positive correlation between PCB126 dose and the number of genes differentially expressed (Table 2). A similar dose response relationship has been reported for female mice subjected to an acute exposure to PCB126 [31]. Comparative analysis of the hepatic expression profiles of chronic (52 weeks) exposure to 30 ng, 300 ng and 1000 ng/kg/day PCB126 identified 16 genes which were differentially expressed at all three concentrations (Table 3). Interestingly, of those 16 genes, *Ccl2 *(chemokine (C-C motif) ligand 2), *Chka *(choline kinase alpha), *Thrb *(thyroid hormone receptor beta) and *Synj2 *(synaptojanin 2) are not present in the 13- and 52 week hepatic gene expression signature of PCB126 (Figure 2). This indicates that even though differential expression of *Ccl2*, *Chka*, *Thrb *and *Synj2 *are sensitive endpoints of chronic PCB126 exposure, as evident in their responsiveness at 30 ng/kg/day PCB126, these changes do not manifest themselves following 13 weeks of subchronic exposure to 1000 ng/kg/day PCB126. These four genes help illustrate the caution that one must use in categorizing a gene as a biomarker of exposure. As seen in these results, *Ccl2*, *Chka*, *Thrb *and *Synj2 *are examples of sensitive genomic responses to chronic PCB126 exposure, however, they do not exhibit the early subchronic responsiveness that would make them beneficial as biomarkers in early stage identification of PCB126 exposure.

Pathological data shows that continuous exposure to TCDD and PCB126 beyond a period of 13 weeks is necessary to cause the formation of hepatic neoplastic and non-neoplastic lesions [4,5]. Considering the relevance of genomic responses to the toxicity of DLCs, these data suggest that changes in gene expression that are sustained throughout chronic treatment are playing a pivotal role in the development of hepatic lesions. Seventy-five and 70 genes were identified which showed sustained differential expression following subchronic (13 weeks) and chronic (52 weeks) exposure to TCDD and PCB126, respectively (Figures 1 and 2). The sustained differential expression of these genes over a 52 week span suggests that these genes are likely playing an important role in the hepatotoxic effects of TCDD and PCB126. Nine genes showed sustained differential expression following subchronic and chronic exposure to PCB153 (Figures 1 and 2). Only one gene, *Psat1*, was differentially expressed (up-regulated 3- to 8-fold) in the expression signatures of PCB153, TCDD and PCB126 (Figure 3). Psat1 is a phosphoserine aminotransferase involved in serine biosynthesis whose expression has been shown to be up-regulated in colon adenocarcinoma [32], colorectal cancer [33] and breast cancer [34]. Additionally, increased expression of *Psat1 *in colorectal cancer and breast cancer is associated with a poor regression of tumor metastases following therapy [33,34]. The increase expression of *Psat1 *following TCDD, PCB126 and PCB153 treatments suggests that its response is not specific to DLCs. The identification of unique gene expression profiles in Sprague-Dawley rats exposed to DLCs (TCDD or PCB126) versus non-DLCs (PCB153) corroborates similar observations previously reported in ovariectomized C57BL/6 mice [35].

From the hepatic gene expression signatures of PCB126 and TCDD, 41 genomic biomarkers were identified that are shared by both compounds, following 13 and 52 weeks of exposure (Table 4). The observation that these 41 genomic biomarkers are shared by two AhR ligands suggests that differential expression of these genes requires AhR activation. Of the 41 AhR-ligand genomic biomarkers, 30 exhibited a 2-fold or greater change in expression 24 h post-exposure to an acute dose of TCDD, as determined by real-time qPCR. In addition, approximately 40%of these genes have shown a 2-fold change in expression following acute exposure to TCDD in other studies conducted on female and/or male Sprague-Dawley rats [14,27], adding further support that these genomic biomarkers represent time-independent primary responses in gene expression to AhR ligands. Ten of the AhR genomic biomarkers resulted in a less than 2-fold change following acute exposure to TCDD, however, 9 of these biomarkers exhibited a similar trend in the up or down regulation observed following subchronic and chronic exposure to TCDD and PCB126 (Table 4). Furthermore, other microarray studies have shown that following an acute exposure to TCDD, the majority of these genomic biomarkers exhibit a similar response as that seen in our study [14,29,30], thus providing further evidence for their roles as biomarkers.

Seven of the 41 genomic biomarkers are members of the "AhR gene battery" which are a group of genes known to be regulated by the AhR [36]. Genomic biomarker genes which fall into this category include *CYP1a1*, *CYP1a2 *(cytochrome P450 1A2*)*, *CYP1b1*, *Nqo1, Ugt1a6, Ugt1A7 *and *Aldh3a1 *(aldehyde dehydrogenase 3A1*)*. More novel genes included among the 41 genomic biomarkers include genes involved in trafficking/transport (*Cadps, Exoc3*, *Serpina7*, *Slc13a3 *and *Slc29a1*), cell adhesion (*Ceacam10 *and *Enpp2*), cell signaling (*Ptprd*, *Ptprn *and *Trib3*) and development/differentiation (*Enpp3 and Srd5a1*). Enpp2 (Ectonucleotide pyrophosphatase/phosphodiesterase 2; also known as autotaxin), a tumor cell motility stimulating factor [37], was up-regulated following TCDD and PCB126 exposure. This agrees with previous observations implicating *Enpp2 *as being one of the most commonly up-regulated genes in cancer cells and being widely involved in tumor progression, invasion and metastasis [38]. Ptprd (Receptor-type tyrosine-protein phosphatase delta*)*, a protein tyrosine phosphatase, has been identified as a tumor suppressor [39,40] whose expression is down-regulation in breast, colon and glioblastoma tumors [39,41]. The down-regulation of *Ptprd *following TCDD and PCB126 exposure likely contributes to the neoplastic effects of the compounds. Trib3 (Tribbles homolog 3 (Drosophila)) is a regulatory protein which has been shown to be up-regulated following stressful conditions [42,43], consistent with its up-regulation following TCDD and PCB126 exposure.

Twenty-five of the 41 AhR-ligand genomic biomarkers contained one or more putative DRE within 5000 bp upstream and 1000 bp downstream from the transcriptional start site (Table 4). However, genes such as *Cyp3a13 *(cytochrome P450 3A13), *Ces3 *(carboxylesterase 3) and *Serpina7 *(serine (or cysteine) peptidase inhibitor, clade A (alpha-1 antiproteinase, antitrypsin), member 7) did not contain a putative DRE in the region examined (-5000 bp to +1000 bp), suggesting that an activated AhR may not directly bind to these genes. Interestingly, even though *Cyp3a13*, *Ces3 *and *Serpina7 *do not contain any DREs in their promoter region, their acute sensitivity to TCDD has been previously shown to be dependent on a functional AhR [13]. This indicates that the presence or lack of a DRE in the promoter region does not solely determine the response of a gene following TCDD exposure; it is also possible that a DRE located outside the region examined here is able to influence gene expression.

In order to relate changes in gene expression to the observed hepatotoxicity, the 52 week hepatic gene expression profiles from TCDD and PCB126 treated rats were compared to the expression profiles from previously published studies [22,24] that examined hepatic neoplastic lesions similar to those observed in the NTP studies. Through this approach, an attempt was made to identify common genes which may play a role in the development and progression of the neoplastic effects observed with DLCs. This comparison identified 24, 17 and 7 genes which were differentially expressed with exposure to DLCs and human ICC, human HCA and rat HCA, respectively (Tables 5 and 6). Interestingly, of the genes common to both DLC exposure and the examined disease states, *Alas1*, *Cadps *(Ca2+-dependent secretion activator), *Cyp3a13*, *Enpp2*, *Pik3c2g *(phosphatidylinositol 3-kinase, C2 domain containing, gamma polypeptide) and *Trib3 *were also present among the 41 time independent AhR genomic biomarkers. For both of the HCA and ICC studies there were genes which did not overlap between the similar disease states which is likely due to inter-individual differences in the tumor micro-environment, environmental conditions and other genetic components.

The genes *Gata6 *and *Timp3 *were down-regulated in both of the human ICC expression profiles and following TCDD exposure. *Gata6 *was also down-regulated following PCB126 exposure and in the human HCA expression profile. Additionally, the genes *Bhlhb2*, *Agt *and *Gata6 *were down-regulated in the ICC and HCA disease states and following exposure to DLCs. Gata6 is a zinc finger transcription factors which can regulate gene expression and cell cycle progression [44,45]. Expression of *Gata6 *is significantly depressed in most human adrenocortical tumors [46,47] and it has been hypothesized that decreased expression of *Gata6 *may be an important event for the escape of tumor cells from normal control mechanisms [48]. Timp3 is a matrix metalloproteinase with proapoptotic activity [49] whose expression is significantly lower in human cholangiocarcinomas [50]. It has been suggested that *Timp3 *may serve as a tumor suppressor gene in cholangiocarcinoma [50]. *Bhlhb2 *(also known as *Dec1*) is a hypoxia-induced gene whose expression is elevated in several malignant tumors [51-53]. The down-regulation of *Bhlhb2 *in HCA, ICC and following TCDD exposure suggest that these tumor micro-environments are not hypoxic. Agt, is a known precursor of angiotensin I and has shown antitumor effects *in vitro *[54]*and in vivo *[55] by inducing apoptosis and decreasing endothelial cell proliferation [56]. The down-regulation of *Agt *in HCA, ICC and following TCDD exposure likely contributes to the formation of neoplastic lesions.

It should be noted that of the 50 genes shared by the 52-week gene expression data (TCDD, PCB126, and PCB153) and gene expression data from the published reports of ICC and HCA, only 4 genes (*Got2*, *Ugcg*, *Stmn1 *and *Alas1*) were found to be differentially expressed by the non-DLC PCB153. Gene expression of Got2 (glutamic-oxaloacetic transaminase 2, mitochondrial (aspartate aminotransferase 2), a mitochondrial enzyme involved in energy transduction [57], was down-regulated in the PCB153 and human HCA expression profiles while Ugcg (UDP-glucose ceramide glucosyltransferase), an enzyme involved in glycosphingolipid biosynthesis [58], gene expression was up-regulated in these two expression profiles. Stmn1 (stathmin 1), a cellular protein involved in mictotubule destabilization [59], is over expressed in a wide variety of human cancers including liver, breast, lung and prostate cancer [60-63]. *Stmn1 *was up-regulated in the TCDD, PCB153 and human HCA expression profiles suggesting that while it is a good marker for different types of human cancer, it may not be a valid biomarker for DLC exposure in Sprague Dawley rats. Gene expression of *Alas1*, an enzyme involved in heme biosynthesis [64], was down-regulated in the TCDD, PCB126 and human HCA profiles but up-regulated in the PCB153 expression profile, suggesting that down-regulation of *Alas1 *may promote tumor development.

## Conclusions

Toxicogenomic analysis has identified hepatic genomic biomarkers of exposure to the AhR ligands, TCDD and PCB126; and the non-dioxin-like compound, PCB153. From these genomic biomarkers, time-independent hepatic gene expression signatures were constructed that are unique to TCDD, PCB126 and PCB153. In addition to identifying gene expression signatures for the dioxin-like compounds TCDD and PCB126, 41 common genomic biomarkers were identified which are shared by these AhR ligands. These 41 common genomic biomarkers may serve as biomarkers of exposure to other AhR ligands and can be used in the risk assessment of other environmental toxins believed to exert their effect through AhR activation. Together, the data collected in this study can serve to guide future investigations in assessing risk of dioxin-like compounds and elucidating the mechanisms of action by which dioxin-like compounds induce their hepatotoxic and carcinogenic effects.

## Authors' contributions

Microarray analysis was performed by BJO and CMV. Real-time qPCR analysis was performed by CAE. Phenotypic anchoring was performed by BJO and CAE. The project was conceived and designed by JRO. BJO wrote the first draft of the manuscript, which all authors edited and approved.

## Supplementary Material

Additional file 1 **Rat primer sequences used for real-time qPCR analysis**. Oligonucleotide sequences for the forward and reverse primers used for real-time qPCR. Click here for file

Additional file 2 **Microarray gene expression following 52 weeks of chronic p.o. exposure to 30 ng/kg/day PCB126 **A list of the 52 genes differentially expressed following 52 weeks of chronic exposure to 30 ng/kg/day PCB126. A gene was considered to be differentially expressed if it displayed a gene expression fold change of 2 or greater. Click here for file

Additional file 3 **Microarray gene expression following 52 weeks of chronic p.o. exposure to 300 ng/kg/day PCB126 **A list of the 128 genes differentially expressed following 52 weeks of chronic exposure to 300 ng/kg/day PCB126. A gene was considered to be differentially expressed if it displayed a gene expression fold change of 2 or greater. Click here for file

Additional file 4 **Microarray gene expression following 52 weeks of chronic p.o. exposure to 1000 ng/kg/day PCB126 **A list of the 216 genes differentially expressed following 52 weeks of exposure to 1000 ng/kgday PCB126. A gene was considered to be differentially expressed if it displayed a gene expression fold change of 2 or greater. Click here for file

Additional file 5 **Microarray gene expression following 13 weeks of subchronic p.o. exposure to 100 ng/kg/day TCDD **A list of the 103 genes differentially expressed following 13 weeks of subchronic exposure to 100 ng/kg/day TCDD. A gene was considered to be differentially expressed if it displayed a gene expression fold change of 2 or greater. Click here for file

Additional file 6 **Microarray gene expression following 52 weeks of chronic p.o. exposure to 100 ng/kg/day TCDD **A list of the 299 genes differentially expressed genes following 52 weeks of chronic exposure to 100 ng/kg/day TCDD. A gene was considered to be differentially expressed if it displayed a gene expression fold change of 2 or greater. Click here for file

Additional file 7 **Microarray gene expression following 13 weeks of subchronic p.o. exposure to 1000 ng/kg/day PCB126 **A list of the 371 genes differentially expressed genes following 13 weeks of subchronic exposure to 1000 ng/kg/day PCB126. A gene was considered to be differentially expressed if it displayed a gene expression fold change of 2 or greater. Click here for file

Additional file 8 **Microarray gene expression following 13 weeks of subchronic p.o. exposure to 1000 μg/kg/day PCB153 **A list of the 39 genes differentially expressed following 13 weeks of subchronic exposure to 1000 μg/kg/day PCB153. A gene was considered to be differentially expressed if it displayed a gene expression fold change of 2 or greater. Click here for file

Additional file 9 **Microarray gene expression following 52 weeks of chronic p.o. exposure to 1000 μg/kg/day PCB153 **A list of the 47 genes differentially expressed following 52 weeks of chronic exposure to 1000 μg/kg/day PCB153. A gene was considered to be differentially expressed if it displayed a gene expression fold change of 2 or greater. Click here for file
