# Supplementary material for: Toxicogenomic analysis of exposure to TCDD, PCB126 and PCB153: identification of genomic biomarkers of exposure to AhR ligands
Source: BMC Genomics. 2010 Oct 19;11:583. doi: 10.1186/1471-2164-11-583 (PMC3091730; doi:10.1186/1471-2164-11-583)
Supplement: Additional file 1 — Rat primer sequences used for real-time qPCR analysis. Oligonucleotide sequences for the forward and reverse primers used for real-time qPCR. [file 1471-2164-11-583-S1.DOC]

| **Additional file 1: Rat Primer Sequences for Real-time qPCR** | | |
| --- | --- | --- |
| **Gene** | **Left primer** | **Right primer** |
| Cyp1a1 | CTGGTTCTGGATACCCAGCTG | CCTAGGGTTGGTTACCAGG |
| Cyp1b1 | CGTCTGATGCTTTCAGCAAAGG | GCAGGCTTTCCAACTAAGCCAG |
| Ceacam10 | AACCTAGCAGGCAGCAGAGAC | TGAGGCTGTGAGCAGTAGTCC |
| Nqo1 | TTCCAGAAACGACATCACAGG | AGCTACAATATCCGGGCTCAG |
| Ugt1a7 | ACTTTTCTCTCCCGTCAGTGG | TGGGGACATATGAAGGAGGAC |
| Ugt1a6 | CTTACTCTCTGAAAGGATGGCTTG | AGCAGCTTGTCACCTAGAACTGAG |
| Cyp1a2 | GTCACCTCAGGGAATGCTGTG | GTTGACAATCTTCTCCTGAGG |
| Enpp2 | GCCCTGAGATGACAAATCCTC | ATAACGTTCACACAGCGATGC |
| Exoc3 | GTCGCCATCATCAATAACTGC | TGACTCTGACACAGGCTTTCC |
| Aldh3a1 | CTAGTGGTCAAAGGGGGTGTC | ACAATCTTCCCTACGGCTGTG |
| Me1 | GGCAGTCCTTTTGATCCAGTC | CACTACCCCAAGAGCAACTCC |
| Trib3 | TTTTTGGGCCAAGCTAAACTG | GCTTGTCATCCAGACAGCATC |
| Tgfb1i4 | CGAACAACAGCATAGCAGAGG | CTGGAAAGGGAGACATCAAGG |
| Gls2 | AGAGCAAGAGAGGTGGGTGAG | AGCAGTAGCAGCATGTCTTGG |
| Zfp354a | AGATGGCTCCTGAGCAAAGAG | AGATCCAGCTTCTTCCACTCG |
| Alas1 | TCGAAGTGATGAGCGAGAGAG | GGTAATGGTCTGGGGTTTGTG |
| Phyh2 | TACATGTTCGGTGTCGTAGGC | GCTCATTCCTCATCCCAATG |
| Ca2 | TGGTTCACTGGAACACCAAA | GGCAGGTCCAATCTTCAAAA |
| Sfxn1 | GACTCAATGCCCTAACCAAGC | CTCATCAGCGGGATGTTAATG |
| Mtmr7 | GGTCGAACCTGTGGAAGAATC | GGAGCTGTAGGCAGATGAAGG |
| Pik3c2g | CTTCCAGGTTTCCAGACCAAG | AACTAGCTGTGGGAGGCAGTC |
| LOC246263 | AACCACCCAGATGAAATCCAC | CACAGCATCCACTTCCTGAAC |
| Slc29a1 | TCTCTACCCGCCAACAGAAAC | AACAACCCAATGGTGACTGTG |
| Cadps | TTCATCCGTGACCTACACTGG | GACGCAGGACTCAATCATGTC |
| Hal | AGATGTGGTCCCCAAAGAGTG | CAGGCCTTCTTTTGGTTTCAG |
| Cyp3a13 | CCACCAGCATGAAAGACATC | GTCCTGTGGGTTGTTAAGGG |
| Ptprd | ACTTGGGAACATTCGAACCTG | CCTTCTAAAGGCTGCAGGATG |
| Ces3 | GGCCATTTCTGAGAGTGGTGTG | GCAGGCAATGAACCATAACAGC |
| Mgst3 | ACTGTGTGCTCTGCTTTCCAG | AAGGCGACTGAGAGGTGAAAG |
| Serpina7 | TCTGGCTCTAGCACCCAAAC | GATCAAATGCTGGAAGCCC |
| Nr0b2 | GCATTGTGTGAAGTCCTGGAG | GATGTTCTTGAGGGTGGAAGC |
| LOC246266 | TCTGGAGACGGCTAGAGGAAG | TCCCGAAGACCGTGTTTACTC |
| RGD1311155 | TGCATGTTCAACAAAGACGTG | CATGCAGGACTCAAAGCAGAG |
| Ptprn | TTGTTCGCATCTGGAAGTCTG | CTGGAGAACTGGGGAAGTGAC |
| Srd5a1 | AGAGAAGCCTTCCTGCAACA | TAGCCCAAAGGAAGAGCAAA |
| Enpp3 | GACCTCAGCTGTCGAAAATCC | TGTCAATCCTGACGTTCTTGG |
| Pdp2 | CCTTGGCTTTGCAGGTTAGAC | GATGGAACTCGGGATTTTGAG |
| Ugt1a1 | GGTGGGATAAACTGCCTTCAG | GAGACCATGGATCCCAAAGAG |
| Slc13a3 | CCCTCACTCAAGTGGTGGTT | TATTCCAGGGCACTGTCTCC |
| Resp18 | ATTGTACCCCAAGGCATGTTC | GATACTGGGGATGGAGTCTGC |
| 18S | GAGCGAAAGCATTTGCCAAG | GGCATCGTTTATGGTCGGAA |
|  | | |
