# Supplementary material for: Toxicogenomic analysis of exposure to TCDD, PCB126 and PCB153: identification of genomic biomarkers of exposure to AhR ligands
Source: BMC Genomics. 2010 Oct 19;11:583. doi: 10.1186/1471-2164-11-583 (PMC3091730; doi:10.1186/1471-2164-11-583)
Supplement: Additional file 2 — Microarray gene expression following 52 weeks of chronic p.o. exposure to 30 ng/kg/day PCB126 A list of the 52 genes differentially expressed following 52 weeks of chronic exposure to 30 ng/kg/day PCB126. A gene was considered to be differentially expressed if it displayed a gene expression fold change of 2 or greater. [file 1471-2164-11-583-S2.DOC]

| **Additional file 2: List of 52 genes differentially expressed following 52 weeks of chronic p.o. exposure to 30ng/kg/day PCB126** | | | |
| --- | --- | --- | --- |
| Transcript ID | Gene Symbol | Gene Name | Fold Change |
| NM_012540 | Cyp1a1 | Cytochrome P450, family 1, subfamily a, polypeptide 1 | 499* |
| NM_031841 | Scd2 | Stearoyl-Coenzyme A desaturase 2 | 22 |
| NM_012940 | Cyp1b1 | Cytochrome P450, family 1, subfamily b, polypeptide 1 | 18 |
| NM_173339 | Ceacam10 | CEA-related cell adhesion molecule 10 | 17 |
| NM_031530 | Ccl2 | Chemokine (C-C motif) ligand 2 | 5 |
| NM_012531 | Comt | Catechol-O-methyltransferase | 5 |
| NM_012881 | Spp1 | Secreted phosphoprotein 1 | 4 |
| NM_139100 | Slc25a3 | Solute carrier family 25 (mitochondrial carrier; adenine nucleotide translocator), member 3 | 3 |
| NM_001039691 /// NM_057105 | Ugt1a6 | UDP glycosyltransferase 1 family, polypeptide A6 | 3 |
| NM_001014245 | Cpsf7 | Cleavage and polyadenylation specific factor 7, 59kda | 3 |
| NM_130407 | Ugt1a7 | UDP glycosyltransferase 1 family, polypeptide A7 | 3 |
| NM_001004084 | RT1-Bb | RT1 class II, locus Bb | 3 |
| NM_001012215 /// NM_001014773 /// NM_001037135 /// NM_001037137 /// NM_001037139 /// NM_001037140 /// NM_001037153 /// NM_001037154 /// NM_001037156 /// NM_001037158 /// NM_001037159 /// NM_001037337 /// NM_053943 | Pcdhgc3 /// Pcdhga9 /// Pcdhga11 /// Pcdhga7 /// Pcdhga5 /// Pcdhga8 /// Pcdhgb7 /// Pcdhgb5 /// Pcdhga2 /// Pcdhga3 /// Pcdhga10 /// Pcdhga12 /// Pcdhga1 | Protocadherin gamma subfamily C, 3 /// protocadherin gamma subfamily A, 9 /// protocadherin gamma subfamily A, 11 /// protocadherin gamma subfamily A, 7 /// protocadherin gamma subfamily A, 5 /// protocadherin gamma subfamily A, 8 /// protocadherin gamma b7 /// protocadherin gamma subfamily B, 5 /// protocadherin gamma a2 /// protocadherin gamma A3 /// protocadherin gamma subfamily A, 10 /// protocadherin gamma subfamily A, 12 /// protocadherin gamma a1 | 3 |
| NM_017127 | Chka | Choline kinase alpha | 2 |
| NM_012656 | Sparc | Secreted acidic cysteine rich glycoprotein | 2 |
| NM_031569 /// NM_057098 /// XM_001055907 /// XM_345486 | Prpf6 | Pre-mrna processing factor 6 homolog (S. Cerevisiae) | 2 |
| NM_031036 | Gnaq | Guanine nucleotide binding protein, alpha q polypeptide | 2 |
| NM_017353 | Slc7a5 | Solute carrier family 7 (cationic amino acid transporter, y+ system), member 5 | 2 |
| NM_024127 | Gadd45a | Growth arrest and DNA-damage-inducible 45 alpha | 2 |
| NM_012600 | Me1 | Malic enzyme 1 | 2 |
| NM_019203 | Tsx | Testis specific X-linked gene | 2 |
| NM_134326 | Alb | Albumin | 2 |
| NM_030845 | Cxcl1 | Chemokine (C-X-C motif) ligand 1 | 2 |
| NM_053769 | Dusp1 | Dual specificity phosphatase 1 | 2 |
| NM_138913 | Oas1 | 2',5'-oligoadenylate synthetase 1, 40/46kda | -2 |
| XM_001064355 /// XM_342068 | Fam160b1 | Family with sequence similarity 160, member B1 | -2 |
| NM_199081 | Slc35b1 | Solute carrier family 35, member B1 | -2 |
| NM_053589 | Rab14 | RAB14, member RAS oncogene family | -2 |
| NM_057208 /// NM_173111 | Tpm3 | Tropomyosin 3, gamma | -2 |
| NM_024391 | Hsd17b2 | Hydroxysteroid (17-beta) dehydrogenase 2 | -2 |
| XM_001065075 /// XM_341241 | Mxd4 | Max dimerization protein 4 | -2 |
| NM_138905 | Ppap2b | Phosphatidic acid phosphatase type 2B | -2 |
| NM_023991 | Prkaa2 | Protein kinase, AMP-activated, alpha 2 catalytic subunit | -2 |
| NM_022671 | Onecut1 | One cut domain, family member 1 | -2 |
| NM_053883 | Dusp6 | Dual specificity phosphatase 6 | -2 |
| NM_031235 | Pard3 | Par-3 (partitioning defective 3) homolog (C. Elegans) | -2 |
| XM_001078203 /// XM_214035 | Recc1 | Replication factor C 1 | -2 |
| NM_031642 | Klf6 | Kruppel-like factor 6 | -2 |
| NM_012903 | Anp32a | Acidic (leucine-rich) nuclear phosphoprotein 32 family, member A | -3 |
| NM_024369 | Fstl1 | Follistatin-like 1 | -3 |
| NM_001037979 | Adipor2 | Adiponectin receptor 2 | -3 |
| NM_031563 | Ybx1 | Y box protein 1 | -3 |
| NM_053887 | Map3k1 | Mitogen activated protein kinase kinase kinase 1 | -3 |
| NM_012672 | Thrb | Thyroid hormone receptor beta | -3 |
| XR_009418 | LOC688018 | Similar to SH3-domain binding protein 3 | -3 |
| XM_001057445 /// XM_341542 | Cul2 | Cullin 2 | -3 |
| NM_032071 | Synj2 | Synaptojanin 2 | -3 |
| NM_031776 | Gda | Guanine deaminase | -3 |
| XM_001071608 /// XM_213849 | Nfix | Nuclear factor I/X | -3 |
| NM_031051 /// XM_001054317 /// XM_001074688 /// XM_001077597 /// XM_001081626 /// XM_212679 /// XM_575621 /// XR_006037 /// XR_007991 | Mif /// RGD1565459/// LOC365286 /// RGD1560513 /// LOC679748 /// LOC686548 | Macrophage migration inhibitory factor /// similar to ribosomal protein L10a /// similar to Macrophage migration inhibitory factor (MIF) (Phenylpyruvate tautomerase) (Glycosylation-inhibiting factor) (GIF) (Delayed early response protein 6) (DER6) /// similar to macrophage migration inhibitory factor /// similar to Macrophage migration inhibitory factor (MIF) (Phenylpyruvate tautomerase) (Glycosylation-inhibiting factor) (GIF) (Delayed early response protein 6) (DER6) /// similar to Macrophage migration inhibitory factor (MIF) (Phenylpyruvate tautomerase) (Glycosylation-inhibiting factor) (GIF) (Delayed early response protein 6) (DER6) | -3 |
| NM_012988 | Nfia | Nuclear factor I/A | -4 |
| NM_012637 | Ptpn1 | Protein tyrosine phosphatase, non-receptor type 1 | -4 |
| Shown above are a list of differentially expressed genes with a fold change ≥ 2-fold and a p-value < 0.05 as determined by t-test.  * Statistically significant with a p-value of < 0.05 following Benjamini-Hochberg FDR Correction. | | | |
