# Supplementary material for: Toxicogenomic analysis of exposure to TCDD, PCB126 and PCB153: identification of genomic biomarkers of exposure to AhR ligands
Source: BMC Genomics. 2010 Oct 19;11:583. doi: 10.1186/1471-2164-11-583 (PMC3091730; doi:10.1186/1471-2164-11-583)
Supplement: Additional file 3 — Microarray gene expression following 52 weeks of chronic p.o. exposure to 300 ng/kg/day PCB126 A list of the 128 genes differentially expressed following 52 weeks of chronic exposure to 300 ng/kg/day PCB126. A gene was considered to be differentially expressed if it displayed a gene expression fold change of 2 or greater. [file 1471-2164-11-583-S3.DOC]

| **Additional file 3: List of 128 genes differentially expressed following 52 weeks of chronic p.o. exposure to 300ng/kg/day PCB126** | | | |
| --- | --- | --- | --- |
| Transcript ID | Gene Symbol | Gene Name | Fold Change |
| NM_012540 | Cyp1a1 | Cytochrome P450, family 1, subfamily a, polypeptide 1 | 1263* |
| NM_012940 | Cyp1b1 | Cytochrome P450, family 1, subfamily b, polypeptide 1 | 866* |
| NM_173339 | Ceacam10 | CEA-related cell adhesion molecule 10 | 660* |
| NM_031972 | Aldh3a1 | Aldehyde dehydrogenase family 3, member A1 | 152* |
| NM_130407 | Ugt1a7 | UDP glycosyltransferase 1 family, polypeptide A7 | 23* |
| NM_012786 | Cox8h | Cytochrom c oxidase subunit VIII-H (heart/muscle) | 19* |
| NM_001024964 | Exoc3 | Exocyst complex component 3 | 12 |
| NM_001039691 /// NM_057105 | Ugt1a6 | UDP glycosyltransferase 1 family, polypeptide A6 | 11* |
| NM_031530 | Ccl2 | Chemokine (C-C motif) ligand 2 | 11 |
| NM_198738 | Psat1 | Phosphoserine aminotransferase 1 | 9 |
| NM_031620 | Phgdh | 3-phosphoglycerate dehydrogenase | 9 |
| NM_017127 | Chka | Choline kinase alpha | 7* |
| NM_012531 | Comt | Catechol-O-methyltransferase | 6 |
| NM_130741 | Lcn2 | Lipocalin 2 | 6 |
| NM_017006 | G6pdx | Glucose-6-phosphate dehydrogenase X-linked | 6 |
| NM_017000 | Nqo1 | NAD(P)H dehydrogenase, quinone 1 | 6 |
| NM_057104 | Enpp2 | Ectonucleotide pyrophosphatase/phosphodiesterase 2 | 5 |
| NM_019203 | Tsx | Testis specific X-linked gene | 5* |
| XM_001068689 /// XM_001068737 /// XM_001068787 /// XM_220333 | Cyfip2 | Cytoplasmic FMR1 interacting protein 2 | 5 |
| NM_001014100 | Neurl3 | Neuralized homolog 3 (Drosophila) | 4 |
| NM_144755 | Trib3 | Tribbles homolog 3 (Drosophila) | 4 |
| NM_031569 /// NM_057098 /// XM_001055907 /// XM_345486 | Prpf6 /// Oprl1 /// Tcea2 | PRP6 pre-mrna processing factor 6 homolog (S. Cerevisiae) /// Opioid receptor-like 1 /// Transcription elongation factor A (SII), 2 | 4 |
| NM_024127 | Gadd45a | Growth arrest and DNA-damage-inducible 45 alpha | 4 |
| XM_001068218 /// XM_574074 | RGD1564865 | Similar to 20-alpha-hydroxysteroid dehydrogenase | 4* |
| NM_172224 | Impa2 | Inositol (myo)-1(or 4)-monophosphatase 2 | 4 |
| NM_017151 | Rps15 | Ribosomal protein S15 | 3 |
| NM_145878 | Fabp5 | Fatty acid binding protein 5, epidermal | 3 |
| NM_013123 | Il1r1 | Interleukin 1 receptor, type I | 3 |
| NM_177425 | Csrp2 | Cysteine and glycine-rich protein 2 | 3 |
| NM_001012215 /// NM_001014773 /// NM_001037135 /// NM_001037137 /// NM_001037139 /// NM_001037140 /// NM_001037153 /// NM_001037154 /// NM_001037156 /// NM_001037158 /// NM_001037159 /// NM_001037337 /// NM_053943 | Pcdhgc3 /// Pcdhga9 /// Pcdhga11 /// Pcdhga7 /// Pcdhga5 /// Pcdhga8 /// Pcdhgb7 /// Pcdhgb5 /// Pcdhga2 /// Pcdhga3 /// Pcdhga10 /// Pcdhga12 /// Pcdhga1 | Protocadherin gamma subfamily C, 3 /// protocadherin gamma subfamily A, 9 /// protocadherin gamma subfamily A, 11 /// protocadherin gamma subfamily A, 7 /// protocadherin gamma subfamily A, 5 /// protocadherin gamma subfamily A, 8 /// protocadherin gamma b7 /// protocadherin gamma subfamily B, 5 /// protocadherin gamma a2 /// protocadherin gamma A3 /// protocadherin gamma subfamily A, 10 /// protocadherin gamma subfamily A, 12 /// protocadherin gamma a1 | 3 |
| NM_001008847 | RT1-Da | RT1 class II, locus Da | 3 |
| NM_001004235 | Mrpl37 | Mitochondrial ribosomal protein L37 | 2 |
| NM_012600 | Me1 | Malic enzyme 1 | 2* |
| NM_013069 | Cd74 | CD74 antigen (invariant polypeptide of major histocompatibility complex, class II antigen-associated) | 2 |
| NM_031036 | Gnaq | Guanine nucleotide binding protein, alpha q polypeptide | 2 |
| NM_012541 | Cyp1a2 | Cytochrome P450, family 1, subfamily a, polypeptide 2 | 2 |
| NM_019165 | Il18 | Interleukin 18 | 2 |
| XM_001077699 /// XM_001077810 /// XM_001079420 /// XM_001079450 /// XM_001079458 /// XM_001079467 /// XM_001079477 /// XM_001079488 /// XM_001079498 /// XM_001079510 /// XM_001079521 /// XM_001079530 /// XM_341940 | Fgfr2 | Fibroblast growth factor receptor 2 | 2 |
| NM_001009920 | Yc2 | Glutathione S-transferase Yc2 subunit | 2 |
| NM_139089 | Cxcl10 | Chemokine (C-X-C motif) ligand 10 | 2 |
| NM_001012162 | Mllt10 | Myeloid/lymphoid or mixed-lineage leukemia (trithorax homolog, Drosophila); translocated to, 10 | 2 |
| NM_012521 | S100g | S100 calcium binding protein G | 2 |
| NM_030845 | Cxcl1 | Chemokine (C-X-C motif) ligand 1 | 2 |
| NM_031588 | Nrg1 | Neuregulin 1 | 2 |
| NM_012656 | Sparc | Secreted acidic cysteine rich glycoprotein | 2 |
| XM_001076038 /// XM_579602 | Ppat | Phosphoribosyl pyrophosphate amidotransferase | 2 |
| NM_133307 | Prkcd | Protein kinase C, delta | 2 |
| XM_001058806 /// XM_343227 | Nipal2 | NIPA-like domain containing 2 | 2 |
| NM_017259 | Btg2 | B-cell translocation gene 2, anti-proliferative | 2 |
| NM_001040019 | RGD1562373 | Similar to 3-ketoacyl-coa thiolase B, peroxisomal precursor (Beta-ketothiolase B) | 2 |
| NM_016988 | Acp2 | Acid phosphatase 2, lysosomal | 2 |
| XM_001078875 /// XM_341377 | Stk24 | Serine/threonine kinase 24 (STE20 homolog, yeast) | 2 |
| XM_001078048 /// XM_001079634 /// XM_001079646 /// XM_341942 | Plekha1 | Pleckstrin homology domain containing, family A (phosphoinositide binding specific) member 1 | 2 |
| NM_001033073 | Defa-rs1 | Defensin alpha-related sequence 1 | 2 |
| NM_017353 | Slc7a5 | Solute carrier family 7 (cationic amino acid transporter, y+ system), member 5 | 2 |
| NM_021835 | Jun | Jun oncogene | 2 |
| NM_013187 | Plcg1 | Phospholipase C, gamma 1 | -2 |
| XM_001064355 /// XM_342068 | Fam160b1 | Family with sequence similarity 160, member B1 | -2 |
| NM_012716 | Slc16a1 | Solute carrier family 16 (monocarboxylic acid transporters), member 1 | -2 |
| NM_001000508 /// NM_001001111 /// NM_001001112 /// NM_001001113 /// NM_001001114 /// NM_206850 | Olr1696 /// Olr1687 /// Olr1697 /// Olr1699/// Olr1700 /// Olr1701 | Olfactory receptor 1696 /// olfactory receptor 1687 /// olfactory receptor 1697 /// olfactory receptor 1699 /// olfactory receptor 1700 /// olfactory receptor 1701 | -2 |
| XM_001065075 /// XM_341241 | Mxd4 | Max dimerization protein 4 | -2 |
| NM_199081 | Slc35b1 | Solute carrier family 35, member B1 | -2 |
| NM_175766 | Cyp2j9 | Cytochrome P450, family 2, subfamily j, polypeptide 9 | -2 |
| NM_030656 | Agxt | Alanine-glyoxylate aminotransferase | -2 |
| NM_053698 | Cited2 | Cbp/p300-interacting transactivator, with Glu/Asp-rich carboxy-terminal domain, 2 | -2 |
| NM_139086 | Sycn | Syncollin | -2 |
| NM_013219 | Cadps | Ca2+-dependent secretion activator | -2 |
| NM_053463 | Nucb1 | Nucleobindin 1 | -2 |
| NM_001014123 /// XM_001058170 | LOC360689 /// LOC498354 /// LOC499531 /// LOC501173 /// LOC501449 /// LOC689117 | Similar to ORF2 consensus sequence encoding endonuclease and reverse transcriptase minus rnaseh /// hypothetical protein LOC498354 /// nucleic acid binding protein /// hypothetical protein LOC501173 /// hypothetical protein LOC501449 /// hypothetical protein LOC689117 | -2 |
| NM_013197 | Alas2 | Aminolevulinic acid synthase 2 | -2 |
| NM_012578 | H1f0 | H1 histone family, member 0 | -2 |
| NM_080583 | Ap2b1 | Adaptor-related protein complex 2, beta 1 subunit | -2 |
| NM_031642 | Klf6 | Kruppel-like factor 6 | -2 |
| NM_201415 /// XM_001061405 /// XM_001073068 /// XM_001074915 /// XM_217582 /// XM_342480 /// XM_574809 /// XR_005968 /// XR_006185 /// XR_007668 | Rpl17 /// RGD1565956 /// RGD1560017 /// RGD1563903 /// LOC499485 /// LOC499522 | Ribosomal protein L17 /// similar to 60S ribosomal protein L17 (L23) /// similar to Ac2-210 /// hypothetical gene supported by X60212 /// similar to 60S ribosomal protein L17 (L23) (Amino acid starvation-induced protein) (ASI) /// similar to 60S ribosomal protein L17 (L23) (Amino acid starvation-induced protein) (ASI) | -2* |
| NM_022267 | Ccnd2 | Cyclin D2 | -2 |
| XM_001076104 /// XM_213943 | Mgst3 | Microsomal glutathione S-transferase 3 | -2* |
| XM_001068826 /// XM_001071911 | Man2a1 | Mannosidase 2, alpha 1 | -2* |
| XM_001055394 /// XM_218002 | Dact2 | Dapper homolog 2, antagonist of beta-catenin (xenopus) | -2 |
| NM_134365 | Atp5f1 | ATP synthase, H+ transporting, mitochondrial F0 complex, subunit b, isoform 1 | -2 |
| NM_001013889 | LOC291863 | Carboxylesterase-like | -2 |
| NM_017159 | Hal | Histidine ammonia lyase | -2 |
| NM_001037979 | Adipor2 | Adiponectin receptor 2 | -2 |
| NM_001013124 | Ung | Uracil-DNA glycosylase | -2 |
| NM_001034111 /// NM_012630 | Prlr | Prolactin receptor | -2 |
| NM_019291 | Ca2 | Carbonic anhydrase 2 | -2 |
| XM_575338 | RGD1562323 | Similar to fatty acid translocase/CD36 | -2 |
| NM_053883 | Dusp6 | Dual specificity phosphatase 6 | -3 |
| NM_138905 | Ppap2b | Phosphatidic acid phosphatase type 2B | -3 |
| NM_134329 | Adh7 | Alcohol dehydrogenase 7 (class IV), mu or sigma polypeptide | -3* |
| NM_134432 | Agt | Angiotensinogen (serpin peptidase inhibitor, clade A, member 8) | -3 |
| NM_012672 | Thrb | Thyroid hormone receptor beta | -3 |
| NM_022866 | Slc13a3 | Solute carrier family 13 (sodium-dependent dicarboxylate transporter), member 3 | -3 |
| XM_001081296 /// XM_220894 | Hoxb2 | Homeo box B2 | -3 |
| NM_052798 | Zfp354a | Zinc finger protein 354A | -3 |
| NM_017070 | Srd5a1 | Steroid 5 alpha-reductase 1 | -3 |
| NM_031776 | Gda | Guanine deaminase | -3 |
| NM_053887 | Map3k1 | Mitogen activated protein kinase kinase kinase 1 | -3 |
| NM_144750 | Aspg | Aspg asparaginase homolog (S. Cerevisiae) | -3 |
| NM_133545 | Ptpn21 | Protein tyrosine phosphatase, non-receptor type 21 | -3 |
| NM_021589 | Ntrk1 | Neurotrophic tyrosine kinase, receptor, type 1 | -3 |
| XR_009418 | LOC688018 | Similar to SH3-domain binding protein 3 | -3 |
| NM_031983 | Smarcd2 | SWI/SNF related, matrix associated, actin dependent regulator of chromatin, subfamily d, member 2 | -3 |
| NM_032071 | Synj2 | Synaptojanin 2 | -3 |
| NM_001025131 /// NM_012695 | Smp2a /// Sult2a2 | Rat senescence marker protein 2A gene, exons 1 and 2 /// sulfotransferase family 2A, dehydroepiandrosterone (DHEA)-preferring, member 2 | -3 |
| NM_012683 | Ugt1a1 | UDP glycosyltransferase 1 family, polypeptide A1 | -3 |
| NM_001014036 | Larp1b | Larp1b La ribonucleoprotein domain family, member 1B | -3 |
| NM_017206 | Slc6a6 | Solute carrier family 6 (neurotransmitter transporter, taurine), member 6 | -3 |
| XM_001071608 /// XM_213849 | Nfix | Nuclear factor I/X | -3 |
| NM_053626 | Dao1 | D-amino acid oxidase 1 | -3 |
| NM_012903 | Anp32a | Acidic (leucine-rich) nuclear phosphoprotein 32 family, member A | -3 |
| NM_080782 | Cdkn1a | Cyclin-dependent kinase inhibitor 1A | -3 |
| XM_001056688 /// XM_001056760 /// XM_223664 | Ehbp1 | EH domain binding protein 1 | -3 |
| NM_022671 | Onecut1 | One cut domain, family member 1 | -3 |
| NM_017359 | Rab10 | RAB10, member RAS oncogene family | -4 |
| NM_030994 | Itga1 | Integrin alpha 1 | -4 |
| NM_019195 | Cd47 | CD47 antigen (Rh-related antigen, integrin-associated signal transducer) | -4 |
| NM_012988 | Nfia | Nuclear factor I/A | -4 |
| NM_031528 | Rara | Retinoic acid receptor, alpha | -4 |
| NM_031073 | Ntf3 | Neurotrophin 3 | -4 |
| XM_001062335 /// XM_001070917 /// XM_001070953 /// XM_341808 | Cyp2b2 | Cytochrome P450, family 2, subfamily b, polypeptide 2 | -4 |
| NM_012935 | Cryab | Crystallin, alpha B | -4 |
| NM_019292 | Ca3 | Carbonic anhydrase 3 | -4 |
| NM_053539 | Idi1 | Isopentenyl-diphosphate delta isomerase | -4 |
| NM_012637 | Ptpn1 | Protein tyrosine phosphatase, non-receptor type 1 | -5 |
| NM_031563 | Ybx1 | Y box protein 1 | -5 |
| XM_001054915 /// XM_343823 | Serpina7 | Serine (or cysteine) peptidase inhibitor, clade A (alpha-1 antipeptidase, antitrypsin), member 7 | -7 |
| NM_019278 | Resp18 | Regulated endocrine-specific protein 18 | -14 |
| NM_147206 | Cyp3a13 | Cytochrome P450, family 3, subfamily a, polypeptide 13 | -68 |
| Shown above are a list of differentially expressed genes with a fold change ≥ 2-fold and a p-value < 0.05 as determined by t-test.  * Statistically significant with a p-value of < 0.05 following Benjamini-Hochberg FDR Correction. | | | |
