# Supplementary material for: Toxicogenomic analysis of exposure to TCDD, PCB126 and PCB153: identification of genomic biomarkers of exposure to AhR ligands
Source: BMC Genomics. 2010 Oct 19;11:583. doi: 10.1186/1471-2164-11-583 (PMC3091730; doi:10.1186/1471-2164-11-583)
Supplement: Additional file 4 — Microarray gene expression following 52 weeks of chronic p.o. exposure to 1000 ng/kg/day PCB126 A list of the 216 genes differentially expressed following 52 weeks of exposure to 1000 ng/kgday PCB126. A gene was considered to be differentially expressed if it displayed a gene expression fold change of 2 or greater. [file 1471-2164-11-583-S4.DOC]

| **Additional file 4: List of 216 genes differentially expressed following 52 weeks of chronic p.o. exposure to 1000ng/kgday PCB126** | | | |  | |
| --- | --- | --- | --- | --- | --- |
| Transcript ID | Gene Symbol | Gene Name | Fold Change |  |  |
| NM_012940 | Cyp1b1 | Cytochrome P450, family 1, subfamily b, polypeptide 1 | 2091** |  |  |
| NM_012540 | Cyp1a1 | Cytochrome P450, family 1, subfamily a, polypeptide 1 | 1551** |  |  |
| NM_031972 | Aldh3a1 | Aldehyde dehydrogenase family 3, member A1 | 1493** |  |  |
| NM_173339 | Ceacam10 | CEA-related cell adhesion molecule 10 | 976** |  |  |
| XM_001081230 /// XM_213440 | Col1a1 | Procollagen, type 1, alpha 1 | 85** |  |  |
| NM_031732 | Sult1c1 | Sulfotransferase family, cytosolic, 1C, member 1 | 61** |  |  |
| NM_031810 | Defb1 | Defensin beta 1 | 61** |  |  |
| NM_012881 | Spp1 | Secreted phosphoprotein 1 | 31** |  |  |
| NM_031530 | Ccl2 | Chemokine (C-C motif) ligand 2 | 31** |  |  |
| NM_053963 | Mmp12 | Matrix metallopeptidase 12 | 27** |  |  |
| NM_130407 | Ugt1a7 | UDP glycosyltransferase 1 family, polypeptide A7 | 40** |  |  |
| NM_012577 /// NM_138974 | Gstp1 /// Gstp2 | Glutathione-S-transferase, pi 1 /// glutathione S-transferase, pi 2 | 31** |  |  |
| NM_130741 | Lcn2 | Lipocalin 2 | 21** |  |  |
| NM_001014100 | LOC316326 | Similar to lung inducible neuralized-related C3HC4 RING finger protein | 22** |  |  |
| NM_001024964 | Exoc3 | Exocyst complex component 3 | 21** |  |  |
| XM_001067689 /// XM_236992 | Tnfrsf21 | Tumor necrosis factor receptor superfamily, member 21 | 14** |  |  |
| NM_012786 | Cox8h | Cytochrom c oxidase subunit VIII-H (heart/muscle) | 24** |  |  |
| NM_012598 | Lpl | Lipoprotein lipase | 10** |  |  |
| NM_001039691 /// NM_057105 | Ugt1a6 | UDP glycosyltransferase 1 family, polypeptide A6 | 16** |  |  |
| NM_144755 | Trib3 | Tribbles homolog 3 (Drosophila) | 13** |  |  |
| NM_031620 | Phgdh | 3-phosphoglycerate dehydrogenase | 8** |  |  |
| NM_017127 | Chka | Choline kinase alpha | 6** |  |  |
| NM_053401 | Ngfrap1 | Nerve growth factor receptor (TNFRSF16) associated protein 1 | 6** |  |  |
| NM_017000 | Nqo1 | NAD(P)H dehydrogenase, quinone 1 | 9** |  |  |
| XM_001068689 /// XM_001068737 /// XM_001068787 /// XM_220333 | Cyfip2 | Cytoplasmic FMR1 interacting protein 2 | 5** |  |  |
| NM_177425 | Csrp2 | Cysteine and glycine-rich protein 2 | 7** |  |  |
| NM_024129 | Dcn | Decorin | 6 |  |  |
| NM_198738 | Psat1 | Phosphoserine aminotransferase 1 | 5** |  |  |
| NM_017208 | Lbp | Lipopolysaccharide binding protein | 6** |  |  |
| NM_031588 | Nrg1 | Neuregulin 1 | 4** |  |  |
| NM_024127 | Gadd45a | Growth arrest and DNA-damage-inducible 45 alpha | 4** |  |  |
| NM_031569 /// NM_057098 /// XM_001055907 /// XM_345486 | Prpf6 /// Oprl1 /// Tcea2 | Prpf6 PRP6 pre-mrna processing factor 6 homolog (S. Cerevisiae)/// Opioid receptor-like 1 /// Transcription elongation factor A (SII), 2 | 4** |  |  |
| NM_012656 | Sparc | Secreted acidic cysteine rich glycoprotein | 4** |  |  |
| NM_019153 | Fbln5 | Fibulin 5 | 4** |  |  |
| NM_032085 | Col3a1 | Procollagen, type III, alpha 1 | 5** |  |  |
| NM_017006 | G6pdx | Glucose-6-phosphate dehydrogenase X-linked | 5 |  |  |
| XM_001058806 /// XM_343227 | Nipal2 | NIPA-like domain containing 2 | 5** |  |  |
| NM_138541 | Tacstd1 | Tumor-associated calcium signal transducer 1 | 4** |  |  |
| NM_080581 | Abcc3 | ATP-binding cassette, sub-family C (CFTR/MRP), member 3 | 4** |  |  |
| NM_001004235 | Mrpl37 | Mitochondrial ribosomal protein L37 | 4** |  |  |
| NM_012600 | Me1 | Malic enzyme 1 | 4** |  |  |
| NM_012551 | Egr1 | Early growth response 1 | 4** |  |  |
| NM_012752 | Cd24 | CD24 antigen | 4** |  |  |
| NM_172224 | Impa2 | Inositol (myo)-1(or 4)-monophosphatase 2 | 4** |  |  |
| NM_012967 | Icam1 | Intercellular adhesion molecule 1 | 4 |  |  |
| NM_031832 | Lgals3 | Lectin, galactose binding, soluble 3 | 4** |  |  |
| NM_057104 | Enpp2 | Ectonucleotide pyrophosphatase/phosphodiesterase 2 | 5** |  |  |
| NM_012862 | Mgp | Matrix Gla protein | 3** |  |  |
| NM_021835 | Jun | Jun oncogene | 4** |  |  |
| NM_013069 | Cd74 | CD74 antigen (invariant polypeptide of major histocompatibility complex, class II antigen-associated) | 4 |  |  |
| XM_001077699 /// XM_001077810 /// XM_001079420 /// XM_001079450 /// XM_001079458 /// XM_001079467 /// XM_001079477 /// XM_001079488 /// XM_001079498 /// XM_001079510 /// XM_001079521 /// XM_001079530 /// XM_341940 | Fgfr2 | Fibroblast growth factor receptor 2 | 3** |  |  |
| NM_017259 | Btg2 | B-cell translocation gene 2, anti-proliferative | 4** |  |  |
| NM_013144 | Igfbp1 | Insulin-like growth factor binding protein 1 | 4 |  |  |
| NM_012753 | Cyp17a1 | Cytochrome P450, family 17, subfamily a, polypeptide 1 | 5 |  |  |
| NM_022542 | Rhob | Ras homolog gene family, member B | 3** |  |  |
| NM_001040019 | RGD1562373 | Similar to 3-ketoacyl-coa thiolase B, peroxisomal precursor (Beta-ketothiolase B) | 4 |  |  |
| NM_013132 | Anxa5 | Annexin A5 | 3** |  |  |
| NM_145878 | Fabp5 | Fatty acid binding protein 5, epidermal | 3 |  |  |
| NM_001008847 | RT1-Da | RT1 class II, locus Da | 3 |  |  |
| NM_013156 | Ctsl | Cathepsin L | 3** |  |  |
| NM_133307 | Prkcd | Protein kinase C, delta | 3 |  |  |
| NM_001009920 | Yc2 | Glutathione S-transferase Yc2 subunit | 3** |  |  |
| NM_017016 | Hdc | Histidine decarboxylase | 3** |  |  |
| NM_212505 | Ier3 | Immediate early response 3 | 3 |  |  |
| NM_053821 | Ralb | V-ral simian leukemia viral oncogene homolog B | 3** |  |  |
| NM_022525 | Gpx3 | Glutathione peroxidase 3 | 3** |  |  |
| NM_022604 | Esm1 | Endothelial cell-specific molecule 1 | 3 |  |  |
| NM_019905 | Anxa2 | Annexin A2 | 3** |  |  |
| NM_012946 | Sparcl1 | SPARC-like 1 (mast9, hevin) | 2** |  |  |
| NM_001012125 | Loxl1 | Lysyl oxidase-like 1 | 4 |  |  |
| NM_053874 | Cap2 | CAP, adenylate cyclase-associated protein, 2 (yeast) | 3** |  |  |
| NM_012541 | Cyp1a2 | Cytochrome P450, family 1, subfamily a, polypeptide 2 | 2** |  |  |
| NM_019283 | Slc3a2 | Solute carrier family 3 (activators of dibasic and neutral amino acid transport), member 2 | 3** |  |  |
| NM_001004084 | RT1-Bb | RT1 class II, locus Bb | 3 |  |  |
| XM_001062488 /// XM_001070713 | LOC682651 /// LOC689415 | Similar to Metallothionein-2 (MT-2) (Metallothionein-II) (MT-II) /// similar to Metallothionein-2 (MT-2) (Metallothionein-II) (MT-II) | 3 |  |  |
| NM_030987 | Gnb1 | Guanine nucleotide binding protein, beta 1 | 3** |  |  |
| NM_013215 | Akr7a3 | Aldo-keto reductase family 7, member A3 (aflatoxin aldehyde reductase) | 2 |  |  |
| NM_016988 | Acp2 | Acid phosphatase 2, lysosomal | 2** |  |  |
| NM_001034090 /// NM_012844 | Ephx1 | Epoxide hydrolase 1, microsomal | 2** |  |  |
| NM_012771 | Lyz | Lysozyme | 2 |  |  |
| NM_012515 | Bzrp | Benzodiazepine receptor, peripheral | 2** |  |  |
| NM_013123 | Il1r1 | Interleukin 1 receptor, type I | 4** |  |  |
| NM_013089 | Gys2 | Glycogen synthase 2 | 2** |  |  |
| NM_017014 | Gstm1 | Glutathione S-transferase, mu 1 | 2** |  |  |
| NM_001039204 /// NM_001039205 | LOC290071 /// LOC290098 /// LOC290150 /// LOC364343 /// RGD1359684 | Similar to RIKEN cdna A430107P09 gene /// similar to T cell receptor V-alpha J-alpha /// similar to RIKEN cdna A430107P09 gene /// T cell receptor alpha chain V region /// similar to T-cell receptor alpha chain precursor V and C regions (TRA29) | -2** |  |  |
| NM_021653 | Dio1 | Deiodinase, iodothyronine, type I | -2** |  |  |
| NM_013026 | Sdc1 | Syndecan 1 | -2** |  |  |
| XM_575828 | RGD1562988 | Similar to EHM2 | -2** |  |  |
| NM_001007004 | Tuba4 | Tubulin, alpha 4 | -2** |  |  |
| NM_138884 | Akr1d1 | Aldo-keto reductase family 1, member D1 | -2 |  |  |
| NM_133558 | Cml1 | Camello-like 1 | -2 |  |  |
| NM_001009632 | G0s2 | G0/G1 switch gene 2 | -3 |  |  |
| NM_177928 | Pbef1 | Pre-B-cell colony enhancing factor 1 | -3 |  |  |
| NM_012493 | Afp | Alpha-fetoprotein | -2** |  |  |
| NM_012969 | Irs1 | Insulin receptor substrate 1 | -2** |  |  |
| NM_053537 | Slc22a7 | Solute carrier family 22 (organic anion transporter), member 7 | -2** |  |  |
| NM_001012213 | Sfxn1 | Sideroflexin 1 | -3** |  |  |
| NM_017193 | Aadat | Aminoadipate aminotransferase | -2** |  |  |
| NM_017158 | Cyp2c7 | Cytochrome P450, family 2, subfamily c, polypeptide 7 | -2 |  |  |
| XM_001081578 /// XM_221047 | Polg2 | Polymerase (DNA directed), gamma 2, accessory subunit | -2 |  |  |
| XM_001054250 | LOC679161 | Similar to transmembrane protein 64 | -3** |  |  |
| NM_019185 | Gata6 | GATA binding protein 6 | -4** |  |  |
| NM_080767 | Psmb8 | Proteosome (prosome, macropain) subunit, beta type 8 | -2** |  |  |
| NM_031741 | Slc2a5 | Solute carrier family 2, member 5 | -4** |  |  |
| NM_013078 | Otc | Ornithine transcarbamylase | -3** |  |  |
| NM_001014166 | Il33 | Interleukin 33 | -3** |  |  |
| NM_001007235 | Itpr1 | Inositol 1,4,5-triphosphate receptor 1 | -3** |  |  |
| NM_012545 | Ddc | Dopa decarboxylase | -3** |  |  |
| NM_001005383 | Acy1 | Aminoacylase 1 | -2** |  |  |
| NM_019140 | Ptprd | Protein tyrosine phosphatase, receptor type, D | -3 |  |  |
| NM_133418 | Slc25a10 | Solute carrier family 25 (mitochondrial carrier; dicarboxylate transporter), member 10 | -3 |  |  |
| NM_001013853 | LOC287167 | Globin, alpha | -2 |  |  |
| NM_053539 | Idi1 | Isopentenyl-diphosphate delta isomerase | -2** |  |  |
| NM_173322 | Pnrc1 | Proline rich 2 | -3** |  |  |
| NM_017094 | Ghr | Growth hormone receptor | -3** |  |  |
| NM_053493 | Phyh2 | Phytanoyl-coa 2-hydroxylase 2 | -3** |  |  |
| NM_031753 | Alcam | Activated leukocyte cell adhesion molecule | -2** |  |  |
| NM_031975 | Ptms | Parathymosin | -2** |  |  |
| NM_012744 | Pc | Pyruvate carboxylase | -3** |  |  |
| NM_019249 | Ptprf | Protein tyrosine phosphatase, receptor type, F | -3** |  |  |
| NM_021593 | Kmo | Kynurenine 3-monooxygenase (kynurenine 3-hydroxylase) | -2** |  |  |
| NM_022860 | B4galnt1 | Beta-1,4-N-acetyl-galactosaminyl transferase 1 | -2** |  |  |
| NM_173305 | Hsd17b9 | Hydroxysteroid (17-beta) dehydrogenase 9 | -2** |  |  |
| NM_001014206 | RGD1309534 | Similar to RIKEN cdna 4931406C07 | -2** |  |  |
| NM_024484 | Alas1 | Aminolevulinic acid synthase 1 | -3 |  |  |
| XR_008772 | RGD1565350 | Similar to Shb protein | -3** |  |  |
| NM_001012061 | Cnksr3 | Cnksr family member 3 | -3** |  |  |
| XM_001063315 | Itga2b | Itga2b integrin, alpha 2b | -3** |  |  |
| NM_019303 | Cyp2f2 | Cytochrome P450, family 2, subfamily f, polypeptide 2 | -2** |  |  |
| NM_012899 | Alad | Aminolevulinate, delta-, dehydratase | -3** |  |  |
| NM_133623 | Slc6a13 | Solute carrier family 6 (neurotransmitter transporter, GABA), member 13 | -3** |  |  |
| NM_012842 | Egf | Epidermal growth factor | -3 |  |  |
| XM_001067936 /// XM_233065 | RGD1561090 | Similar to protein tyrosine phosphatase, receptor type, D | -2 |  |  |
| NM_144730 | Gata4 | GATA binding protein 4 | -2** |  |  |
| NM_138912 | Ppp1r3b | Protein phosphatase 1, regulatory (inhibitor) subunit 3B | -3** |  |  |
| NM_031648 | Fxyd1 | FXYD domain-containing ion transport regulator 1 | -2** |  |  |
| XM_575338 | RGD1562323 | Similar to fatty acid translocase/CD36 | -3** |  |  |
| XM_001066628 /// XM_342763 | Fkbp4 | FK506 binding protein 4 | -3** |  |  |
| NM_022667 | Slco2a1 | Solute carrier organic anion transporter family, member 2a1 | -3** |  |  |
| NM_001025131 /// NM_012695 | Smp2a /// Sult2a2 | Rat senescence marker protein 2A gene, exons 1 and 2 /// sulfotransferase family 2A, dehydroepiandrosterone (DHEA)-preferring, member 2 | -4 |  |  |
| NM_139086 | Sycn | Syncollin | -3** |  |  |
| NM_019291 | Ca2 | Carbonic anhydrase 2 | -6** |  |  |
| NM_013197 | Alas2 | Aminolevulinic acid synthase 2 | -3 |  |  |
| NM_031855 | Khk | Ketohexokinase | -2** |  |  |
| XM_214551 | Cidea | Cell death-inducing DNA fragmentation factor, alpha subunit-like effector A | -4 |  |  |
| NM_133525 | RGD620382 | Nucleoside 2-deoxyribosyltransferase domain containing protein RGD620382 | -3** |  |  |
| NM_080782 | Cdkn1a | Cyclin-dependent kinase inhibitor 1A | -3 |  |  |
| NM_013219 | Cadps | Ca2+-dependent secretion activator | -3 |  |  |
| XM_001054597 /// XM_213944 | Ppox | Protoporphyrinogen oxidase | -3** |  |  |
| NM_016998 | Cpa1 | Carboxypeptidase A1 | -3 |  |  |
| NM_057133 | Nr0b2 | Nuclear receptor subfamily 0, group B, member 2 | -3** |  |  |
| NM_031025 | Dlat | Dihydrolipoamide S-acetyltransferase (E2 component of pyruvate dehydrogenase complex) | -3** |  |  |
| XM_001076124 /// XM_001076147 /// XM_001076171 /// XM_341825 | Prodh2 | Proline dehydrogenase (oxidase) 2 | -3** |  |  |
| NM_022501 | Crip2 | Cysteine-rich protein 2 | -3** |  |  |
| NM_031684 | Slc29a1 | Solute carrier family 29 (nucleoside transporters), member 1 | -3** |  |  |
| NM_031649 | Klrg1 | Killer cell lectin-like receptor subfamily G, member 1 | -3** |  |  |
| NM_172320 | Afm | Afamin | -3** |  |  |
| NM_001000980 | Olr1366 | Olfactory receptor 1366 | -5 |  |  |
| NM_019370 | Enpp3 | Ectonucleotide pyrophosphatase/phosphodiesterase 3 | -5** |  |  |
| NM_131906 | Slco1a4 | Solute carrier organic anion transporter family, member 1a4 | -3 |  |  |
| NM_012672 | Thrb | Thyroid hormone receptor beta | -3** |  |  |
| NM_053923 | Pik3c2g | Phosphatidylinositol 3-kinase, C2 domain containing, gamma polypeptide | -3** |  |  |
| NM_019373 | Apom | Apolipoprotein M | -3** |  |  |
| NM_133545 | Ptpn21 | Protein tyrosine phosphatase, non-receptor type 21 | -3** |  |  |
| NM_012651 | Slc4a1 | Solute carrier family 4, member 1 | -3 |  |  |
| NM_031561 | Cd36 | Cd36 antigen | -4** |  |  |
| NM_053881 | Ptprn | Protein tyrosine phosphatase, receptor type, N | -4** |  |  |
| NM_012683 | Ugt1a1 | UDP glycosyltransferase 1 family, polypeptide A1 | -3** |  |  |
| XM_001055394 /// XM_218002 | Dact2 | Dapper homolog 2, antagonist of beta-catenin (xenopus) | -3** |  |  |
| XM_001081296 /// XM_220894 | Hoxb2 | Homeo box B2 | -7 |  |  |
| NM_022866 | Slc13a3 | Solute carrier family 13 (sodium-dependent dicarboxylate transporter), member 3 | -4** |  |  |
| NM_175766 | Cyp2j9 | Cytochrome P450, family 2, subfamily j, polypeptide 9 | -4** |  |  |
| XM_001070075 /// XM_342570 | Sgk2 | Serum/glucocorticoid regulated kinase 2 | -4** |  |  |
| NM_031039 | Gpt1 | Glutamic pyruvic transaminase 1, soluble | -4 |  |  |
| XM_001076104 /// XM_213943 | Mgst3 | Microsomal glutathione S-transferase 3 | -4** |  |  |
| NM_134329 | Adh7 | Alcohol dehydrogenase 7 (class IV), mu or sigma polypeptide | -4** |  |  |
| NM_031048 | Lifr | Leukemia inhibitory factor receptor | -5** |  |  |
| NM_017206 | Slc6a6 | Solute carrier family 6 (neurotransmitter transporter, taurine), member 6 | -3** |  |  |
| NM_012988 | Nfia | Nuclear factor I/A | -3** |  |  |
| NM_017274 | Gpam | Glycerol-3-phosphate acyltransferase, mitochondrial | -6** |  |  |
| NM_017332 | Fasn | Fatty acid synthase | -5 |  |  |
| NM_012737 | Apoa4 | Apolipoprotein A-IV | -4** |  |  |
| NM_032071 | Synj2 | Synaptojanin 2 | -4** |  |  |
| NM_001013124 | Ung | Uracil-DNA glycosylase | -4** |  |  |
| XM_001058099 /// XM_240417 | Mtmr7 | Myotubularin related protein 7 | -5** |  |  |
| NM_022180 | Hnf4a | Hepatocyte nuclear factor 4, alpha | -3** |  |  |
| NM_017235 | Hsd17b7 | Hydroxysteroid (17-beta) dehydrogenase 7 | -4** |  |  |
| XM_001072618 /// XM_001072656 /// XM_573819 | Tgfb1i4 | Transforming growth factor beta 1 induced transcript 4 | -6** |  |  |
| NM_001039031 | Dak | Dihydroxyacetone kinase 2 homolog (S. Cerevisiae) | -5** |  |  |
| NM_019292 | Ca3 | Carbonic anhydrase 3 | -5 |  |  |
| XM_001080576 /// XM_234277 | RGD1305721 | Similar to RIKEN cdna 2810055F11 | -4** |  |  |
| NM_022251 | Enpep | Glutamyl aminopeptidase | -5** |  |  |
| NM_138904 | Gls2 | Glutaminase 2 (liver, mitochondrial) | -6** |  |  |
| NM_053698 | Cited2 | Cbp/p300-interacting transactivator, with Glu/Asp-rich carboxy-terminal domain, 2 | -4** |  |  |
| NM_031073 | Ntf3 | Neurotrophin 3 | -5 |  |  |
| NM_001034111 /// NM_012630 | Prlr | Prolactin receptor | -5** |  |  |
| XM_001071608 /// XM_213849 | Nfix | Nuclear factor I/X | -5** |  |  |
| NM_052798 | Zfp354a | Zinc finger protein 354A | -8** |  |  |
| NM_133295 | Ces3 | Carboxylesterase 3 | -9** |  |  |
| NM_145091 | Pdp2 | Pyruvate dehydrogenase phosphatase isoenzyme 2 | -9** |  |  |
| NM_001013137 | Cxcl14 | Chemokine (C-X-C motif) ligand 14 | -10** |  |  |
| XM_001056688 /// XM_001056760 /// XM_223664 | Ehbp1 | EH domain binding protein 1 | -10 |  |  |
| NM_001013083 | Cpa2 | Carboxypeptidase A2 (pancreatic) | -9** |  |  |
| NM_001037979 | Adipor2 | Adiponectin receptor 2 | -6** |  |  |
| NM_021589 | Ntrk1 | Neurotrophic tyrosine kinase, receptor, type 1 | -10** |  |  |
| NM_144748 | LOC246263 | Kidney-specific protein (KS) | -12** |  |  |
| NM_012565 | Gck | Glucokinase | -13** |  |  |
| NM_017070 | Srd5a1 | Steroid 5 alpha-reductase 1 | -14** |  |  |
| XM_001062335 /// XM_001070917 /// XM_001070953 /// XM_341808 | Cyp2b2 | Cytochrome P450, family 2, subfamily b, polypeptide 2 | -9 |  |  |
| NM_017159 | Hal | Histidine ammonia lyase | -17** |  |  |
| XM_001054915 /// XM_343823 | Serpina7 | Serine (or cysteine) peptidase inhibitor, clade A (alpha-1 antipeptidase, antitrypsin), member 7 | -21** |  |  |
| NM_019278 | Resp18 | Regulated endocrine-specific protein 18 | -15** |  |  |
| XM_001067025 /// XM_001072862 /// XM_001081261 /// XM_001081265 /// XM_001081267 /// XM_213437 /// XM_224699 | Spop /// Tspan14 | Speckle-type POZ protein /// Transcribed locus /// Tetraspanin 14 | -18** |  |  |
| NM_144750 | Aspg | Asparaginase homolog (S. Cerevisiae) | -41** |  |  |
| NM_053626 | Dao1 | D-amino acid oxidase 1 | -58** |  |  |
| NM_147206 | Cyp3a13 | Cytochrome P450, family 3, subfamily a, polypeptide 13 | -610** |  |  |
| List of differentially expressed genes with a fold change ≥ 2-fold and a p-value < 0.05 as determined by t-test.  **Statistically significant with a p-value of < 0.05 following Benjamini-Hochberg Correction | | | |  | |
