# Supplementary material for: Toxicogenomic analysis of exposure to TCDD, PCB126 and PCB153: identification of genomic biomarkers of exposure to AhR ligands
Source: BMC Genomics. 2010 Oct 19;11:583. doi: 10.1186/1471-2164-11-583 (PMC3091730; doi:10.1186/1471-2164-11-583)
Supplement: Additional file 5 — Microarray gene expression following 13 weeks of subchronic p.o. exposure to 100 ng/kg/day TCDD A list of the 103 genes differentially expressed following 13 weeks of subchronic exposure to 100 ng/kg/day TCDD. A gene was considered to be differentially expressed if it displayed a gene expression fold change of 2 or greater. [file 1471-2164-11-583-S5.DOC]

| **Additional file 5: List of 103 genes differentially expressed following 13 weeks of subchronic p.o. exposure to 100ng/kg/day TCDD** | | | |
| --- | --- | --- | --- |
| Transcript ID | Gene Symbol | Gene Name | Fold Change |
| NM_012540 | Cyp1a1 | Cytochrome P450, family 1, subfamily a, polypeptide 1 | 1026* |
| NM_012940 | Cyp1b1 | Cytochrome P450, family 1, subfamily b, polypeptide 1 | 743* |
| NM_173339 | Ceacam10 | CEA-related cell adhesion molecule 10 | 620* |
| NM_012786 | Cox8h | Cytochrom c oxidase subunit VIII-H (heart/muscle) | 312* |
| NM_130407 | Ugt1a7 | UDP glycosyltransferase 1 family, polypeptide A7 | 85* |
| NM_031972 | Aldh3a1 | Aldehyde dehydrogenase family 3, member A1 | 57* |
| NM_001039691 /// NM_057105 | Ugt1a6 | UDP glycosyltransferase 1 family, polypeptide A6 | 56* |
| NM_001024964 | Exoc3 | Exocyst complex component 3 | 32* |
| NM_017000 | Nqo1 | NAD(P)H dehydrogenase, quinone 1 | 20 |
| NM_012543 | Dbp | D site albumin promoter binding protein | 9 |
| NM_130741 | Lcn2 | Lipocalin 2 | 8 |
| NM_144755 | Trib3 | Tribbles homolog 3 (Drosophila) | 8* |
| NM_053874 | Cap2 | CAP, adenylate cyclase-associated protein, 2 (yeast) | 7 |
| NM_057104 | Enpp2 | Ectonucleotide pyrophosphatase/phosphodiesterase 2 | 6* |
| NM_001014100 | Neurl3 | Neuralized homolog 3 (Drosophila) | 6 |
| NM_017006 | G6pdx | Glucose-6-phosphate dehydrogenase X-linked | 5 |
| NM_145775 | Nr1d1 | Nuclear receptor subfamily 1, group D, member 1 | 5 |
| NM_198738 | Psat1 | Phosphoserine aminotransferase 1 | 5 |
| NM_012541 | Cyp1a2 | Cytochrome P450, family 1, subfamily a, polypeptide 2 | 5 |
| NM_031841 | Scd2 | Stearoyl-Coenzyme A desaturase 2 | 5 |
| XM_001062488 /// XM_001070713 | LOC682651 /// LOC689415 | Similar to Metallothionein-2 (MT-2) (Metallothionein-II) (MT-II) /// similar to Metallothionein-2 (MT-2) (Metallothionein-II) (MT-II) | 4 |
| NM_016988 | Acp2 | Acid phosphatase 2, lysosomal | 3 |
| NM_012541 | Cyp1a2 | Cytochrome P450, family 1, subfamily a, polypeptide 2 | 3* |
| XM_001058806 /// XM_343227 | Nipal2 | NIPA-like domain containing 2 | 3 |
| NM_031620 | Phgdh | 3-phosphoglycerate dehydrogenase | 3 |
| NM_031530 | Ccl2 | Chemokine (C-C motif) ligand 2 | 3 |
| NM_138840 | Tgoln2 | Trans-golgi network protein 2 | 3 |
| NM_012521 | S100g | S100 calcium binding protein G | 3 |
| NM_001010921 | LOC494499 | LOC494499 protein | 3 |
| NM_012600 | Me1 | Malic enzyme 1 | 3 |
| NM_031588 | Nrg1 | Neuregulin 1 | 3* |
| NM_172224 | Impa2 | Inositol (myo)-1(or 4)-monophosphatase 2 | 3 |
| NM_001024276 | Mettl7b | Methyltransferase like 7B | 3 |
| NM_031810 | Defb1 | Defensin beta 1 | 3 |
| NM_145878 | Fabp5 | Fatty acid binding protein 5, epidermal | 2 |
| XM_001055696 /// XM_001072336 | LOC680097 /// LOC684887 | Similar to germinal histone H4 gene /// similar to germinal histone H4 gene | 2 |
| NM_032085 | Col3a1 | Procollagen, type III, alpha 1 | 2 |
| NM_012515 | Bzrp | Benzodiazepine receptor, peripheral | 2 |
| NM_019283 | Slc3a2 | Solute carrier family 3 (activators of dibasic and neutral amino acid transport), member 2 | 2 |
| NM_031789 | Nfe2l2 | Nuclear factor, erythroid derived 2, like 2 | 2 |
| NM_138826 | Mt1a | Metallothionein 1a | 2 |
| NM_175707 | Ppil3 | Peptidylprolyl isomerase (cyclophilin)-like 3 | 2 |
| NM_001011901 | Hsph1 | Heat shock 105kda/110kda protein 1 | 2 |
| NM_017127 | Chka | Choline kinase alpha | 2 |
| XM_001054505 /// XM_340973 | Ttc3 | Tetratricopeptide repeat domain 3 | -2 |
| NM_017193 | Aadat | Aminoadipate aminotransferase | -2 |
| NM_138884 | Akr1d1 | Aldo-keto reductase family 1, member D1 | -2 |
| NM_012603 | Myc | Myelocytomatosis viral oncogene homolog (avian) | -2 |
| NM_031720 | Dio2 | Deiodinase, iodothyronine, type II | -2 |
| XM_001081576 /// XM_573211 | Ern1 | Endoplasmic reticulum to nucleus signaling 1 | -2 |
| NM_012588 | Igfbp3 | Insulin-like growth factor binding protein 3 | -2 |
| NM_031561 | Cd36 | Cd36 antigen | -2 |
| NM_022860 | B4galnt1 | Beta-1,4-N-acetyl-galactosaminyl transferase 1 | -2 |
| NM_057133 | Nr0b2 | Nuclear receptor subfamily 0, group B, member 2 | -2 |
| XM_001064051 /// XM_575876 | RGD1564074 | Similar to novel protein | -2 |
| NM_031707 | Homer1 | Homer homolog 1 (Drosophila) | -2 |
| NM_001014161 | Idh2 | Isocitrate dehydrogenase 2 (NADP+), mitochondrial | -2 |
| NM_012792 | Fmo1 | Flavin containing monooxygenase 1 | -2 |
| NM_031135 | Klf10 | Kruppel-like factor 10 | -2 |
| NM_053923 | Pik3c2g | Phosphatidylinositol 3-kinase, C2 domain containing, gamma polypeptide | -2 |
| NM_024484 | Alas1 | Aminolevulinic acid synthase 1 | -2 |
| NM_052798 | Zfp354a | Zinc finger protein 354A | -2 |
| NM_001013083 | Cpa2 | Carboxypeptidase A2 (pancreatic) | -2 |
| NM_022508 | Mthfd1 | Methylenetetrahydrofolate dehydrogenase (NADP+ dependent), methenyltetrahydrofolate cyclohydrolase, formyltetrahydrofolate synthase | -2* |
| XM_001072618 /// XM_001072656 /// XM_573819 | Tgfb1i4 /// LOC498545 | Transforming growth factor beta 1 induced transcript 4 /// similar to transforming growth factor beta 1 induced transcript 4 isoform 1 | -2 |
| NM_001014123 /// XM_001058170 | LOC360689 /// LOC498354 /// LOC499531 /// LOC501173 /// LOC501449 /// LOC689117 | Similar to ORF2 consensus sequence encoding endonuclease and reverse transcriptase minus rnaseh /// hypothetical protein LOC498354 /// nucleic acid binding protein /// hypothetical protein LOC501173 /// hypothetical protein LOC501449 /// hypothetical protein LOC689117 | -2 |
| NM_017159 | Hal | Histidine ammonia lyase | -2 |
| NM_019140 | Ptprd | Protein tyrosine phosphatase, receptor type, D | -2 |
| XM_001080576 /// XM_234277 | RGD1305721 | Similar to RIKEN cdna 2810055F11 | -3 |
| XM_001064851 /// XM_001065579 /// XM_001065640 /// XM_213898 | Ivns1abp | Influenza virus NS1A binding protein | -3* |
| NM_145091 | Pdp2 | Pyruvate dehydrogenase phosphatase isoenzyme 2 | -3 |
| NM_016998 | Cpa1 | Carboxypeptidase A1 | -3 |
| XM_001076104 /// XM_213943 | Mgst3 | Microsomal glutathione S-transferase 3 | -3 |
| NM_001012213 | Sfxn1 | Sideroflexin 1 | -3* |
| XM_001058099 /// XM_240417 | Mtmr7 | Myotubularin related protein 7 | -3 |
| NM_138863 | Ltb4dh | Leukotriene B4 12-hydroxydehydrogenase | -3 |
| NM_138512 | Cyp2c70 | Cytochrome P450, family 2, subfamily c, polypeptide 70 | -3 |
| NM_031684 | Slc29a1 | Solute carrier family 29 (nucleoside transporters), member 1 | -3 |
| NM_053881 | Ptprn | Protein tyrosine phosphatase, receptor type, N | -3 |
| XM_001081296 /// XM_220894 | Hoxb2 | Homeo box B2 | -3 |
| NM_175766 | Cyp2j9 | Cytochrome P450, family 2, subfamily j, polypeptide 9 | -3 |
| XM_001059586 /// XM_001059704 /// XM_001059759 /// XM_001059813 /// XM_001059879 /// XM_001059950 /// XM_001060012 /// XM_001064131 /// XM_342854 | Nfib | Nuclear factor I/B | -3 |
| NM_001014166 | Il33 | Interleukin 33 | -3 |
| NM_138904 | Gls2 | Glutaminase 2 (liver, mitochondrial) | -3 |
| NM_012683 | Ugt1a1 | UDP glycosyltransferase 1 family, polypeptide A1 | -3 |
| NM_013219 | Cadps | Ca2+-dependent secretion activator | -3 |
| NM_131906 | Slco1a4 | Solute carrier organic anion transporter family, member 1a4 | -3 |
| NM_053493 | Phyh2 | Phytanoyl-coa 2-hydroxylase 2 | -4 |
| NM_031073 | Ntf3 | Neurotrophin 3 | -4 |
| NM_019370 | Enpp3 | Ectonucleotide pyrophosphatase/phosphodiesterase 3 | -4* |
| NM_001014264 | LOC366772 | Similar to immunoglobulin heavy chain | -4 |
| NM_001017496 | Cxcl13 | Chemokine (C-X-C motif) ligand 13 | -4 |
| NM_017206 | Slc6a6 | Solute carrier family 6 (neurotransmitter transporter, taurine), member 6 | -5 |
| NM_017070 | Srd5a1 | Steroid 5 alpha-reductase 1 | -6 |
| NM_019278 | Resp18 | Regulated endocrine-specific protein 18 | -6 |
| NM_022866 | Slc13a3 | Solute carrier family 13 (sodium-dependent dicarboxylate transporter), member 3 | -7 |
| NM_019291 | Ca2 | Carbonic anhydrase 2 | -8* |
| NM_133295 | Ces3 | Carboxylesterase 3 | -9 |
| NM_144750 | Aspg | Asparaginase homolog (S. Cerevisiae) | -10 |
| NM_053626 | Dao1 | D-amino acid oxidase 1 | -14 |
| XM_001054915 /// XM_343823 | Serpina7 | Serine (or cysteine) peptidase inhibitor, clade A (alpha-1 antipeptidase, antitrypsin), member 7 | -15 |
| NM_144748 | Acsm2 | Acyl-coa synthetase medium-chain family member 2 | -26 |
| NM_147206 | Cyp3a13 | Cytochrome P450, family 3, subfamily a, polypeptide 13 | -139* |
| Shown above are a list of differentially expressed genes with a fold change ≥ 2-fold and a p-value < 0.05 as determined by t-test.  * Statistically significant with a p-value of < 0.05 following Benjamini-Hochberg FDR Correction. | | | |
