# Supplementary material for: Toxicogenomic analysis of exposure to TCDD, PCB126 and PCB153: identification of genomic biomarkers of exposure to AhR ligands
Source: BMC Genomics. 2010 Oct 19;11:583. doi: 10.1186/1471-2164-11-583 (PMC3091730; doi:10.1186/1471-2164-11-583)
Supplement: Additional file 6 — Microarray gene expression following 52 weeks of chronic p.o. exposure to 100 ng/kg/day TCDD A list of the 299 genes differentially expressed genes following 52 weeks of chronic exposure to 100 ng/kg/day TCDD. A gene was considered to be differentially expressed if it displayed a gene expression fold change of 2 or greater. [file 1471-2164-11-583-S6.DOC]

| **Additional file 6: List of 299 genes differentially expressed genes following 52 weeks of chronic p.o. exposure to 100ng/kg/day TCDD** | | | |
| --- | --- | --- | --- |
| Transcript ID | Gene Symbol | Gene Name | Fold Change |
| NM_012940 | Cyp1b1 | Cytochrome P450, family 1, subfamily b, polypeptide 1 | 1288* |
| NM_012540 | Cyp1a1 | Cytochrome P450, family 1, subfamily a, polypeptide 1 | 1277* |
| NM_031972 | Aldh3a1 | Aldehyde dehydrogenase family 3, member A1 | 1156* |
| NM_173339 | Ceacam10 | CEA-related cell adhesion molecule 10 | 973* |
| XM_001081230 /// XM_213440 | Col1a1 | Procollagen, type 1, alpha 1 | 67 |
| NM_130407 | Ugt1a7 | UDP glycosyltransferase 1 family, polypeptide A7 | 33* |
| NM_031810 | Defb1 | Defensin beta 1 | 29 |
| NM_053963 | Mmp12 | Matrix metallopeptidase 12 | 29 |
| NM_001014100 | Neurl3 | Neuralized homolog 3 (Drosophila) | 29 |
| NM_031732 | Sult1c1 | Sulfotransferase family, cytosolic, 1C, member 1 | 27* |
| NM_012786 | Cox8h | Cytochrom c oxidase subunit VIII-H (heart/muscle) | 27* |
| NM_001024964 | Exoc3 | Exocyst complex component 3 | 23* |
| NM_012577 /// NM_138974 | Gstp1 /// Gstp2 | Glutathione-S-transferase, pi 1 /// glutathione S-transferase, pi 2 | 20 |
| NM_130741 | Lcn2 | Lipocalin 2 | 19 |
| NM_144755 | Trib3 | Tribbles homolog 3 (Drosophila) | 16 |
| NM_031530 | Ccl2 | Chemokine (C-C motif) ligand 2 | 16 |
| NM_031004 | Acta2 | Smooth muscle alpha-actin | 12 |
| NM_001039691 /// NM_057105 | Ugt1a6 | UDP glycosyltransferase 1 family, polypeptide A6 | 12* |
| NM_001014264 | LOC366772 | Similar to immunoglobulin heavy chain | 10 |
| NM_019203 | Tsx | Testis specific X-linked gene | 9 |
| NM_031620 | Phgdh | 3-phosphoglycerate dehydrogenase | 9 |
| NM_012531 | Comt | Catechol-O-methyltransferase | 9 |
| NM_177425 | Csrp2 | Cysteine and glycine-rich protein 2 | 9 |
| NM_053401 | Ngfrap1 | Nerve growth factor receptor (TNFRSF16) associated protein 1 | 8 |
| NM_012598 | Lpl | Lipoprotein lipase | 8 |
| NM_017000 | Nqo1 | NAD(P)H dehydrogenase, quinone 1 | 8* |
| NM_001007729 | Cxcl4 | Chemokine (C-X-C motif) ligand 4 | 8 |
| NM_012752 | Cd24 | CD24 antigen | 7 |
| NM_012881 | Spp1 | Secreted phosphoprotein 1 | 7 |
| XM_001067689 /// XM_236992 | Tnfrsf21 | Tumor necrosis factor receptor superfamily, member 21 | 7 |
| NM_017208 | Lbp | Lipopolysaccharide binding protein | 7 |
| NM_172224 | Impa2 | Inositol (myo)-1(or 4)-monophosphatase 2 | 6 |
| XM_001068689 /// XM_001068737 /// XM_001068787 /// XM_220333 | Cyfip2 | Cytoplasmic FMR1 interacting protein 2 | 6 |
| NM_053874 | Cap2 | CAP, adenylate cyclase-associated protein, 2 (yeast) | 6* |
| XM_001058806 /// XM_343227 | Nipal2 | NIPA-like domain containing 2 | 6 |
| NM_001040019 | RGD1562373 | Similar to 3-ketoacyl-coa thiolase B, peroxisomal precursor (Beta-ketothiolase B) | 5 |
| NM_138836 | Prss8 | Protease, serine, 8 (prostasin) | 5* |
| NM_012551 | Egr1 | Early growth response 1 | 5 |
| NM_012753 | Cyp17a1 | Cytochrome P450, family 17, subfamily a, polypeptide 1 | 5 |
| NM_017127 | Chka | Choline kinase alpha | 5* |
| NM_057104 | Enpp2 | Ectonucleotide pyrophosphatase/phosphodiesterase 2 | 4 |
| NM_013144 | Igfbp1 | Insulin-like growth factor binding protein 1 | 4 |
| NM_031841 | Scd2 | Stearoyl-Coenzyme A desaturase 2 | 4 |
| NM_012967 | Icam1 | Intercellular adhesion molecule 1 | 4 |
| NM_030845 | Cxcl1 | Chemokine (C-X-C motif) ligand 1 | 4 |
| NM_031549 | Tagln | Transgelin | 4 |
| NM_138541 | Tacstd1 | Tumor-associated calcium signal transducer 1 | 4 |
| NM_031832 | Lgals3 | Lectin, galactose binding, soluble 3 | 4 |
| NM_017166 | Stmn1 | Stathmin 1 | 4 |
| NM_001004235 | Mrpl37 | Mitochondrial ribosomal protein L37 | 4 |
| NM_013069 | Cd74 | CD74 antigen (invariant polypeptide of major histocompatibility complex, class II antigen-associated) | 4 |
| NM_017006 | G6pdx | Glucose-6-phosphate dehydrogenase X-linked | 4 |
| NM_017259 | Btg2 | B-cell translocation gene 2, anti-proliferative | 4 |
| NM_024127 | Gadd45a | Growth arrest and DNA-damage-inducible 45 alpha | 4 |
| NM_013132 | Anxa5 | Annexin A5 | 4 |
| NM_053821 | Ralb | V-ral simian leukemia viral oncogene homolog B | 4 |
| NM_145878 | Fabp5 | Fatty acid binding protein 5, epidermal | 4 |
| NM_001008847 | RT1-Da | RT1 class II, locus Da | 4 |
| NM_013156 | Ctsl | Cathepsin L | 4 |
| NM_032085 | Col3a1 | Procollagen, type III, alpha 1 | 4 |
| NM_001014123 /// NM_001044260 /// XM_001058170 | LOC360689 /// LOC498354 /// LOC498973 /// LOC499531 /// LOC501173 /// LOC689117 | Similar to ORF2 consensus sequence encoding endonuclease and reverse transcriptase minus rnaseh /// hypothetical protein LOC498354 /// nucleic acid binding protein /// nucleic acid binding protein /// hypothetical protein LOC501173 /// hypothetical protein LOC689117 | 4 |
| NM_138827 | Slc2a1 | Solute carrier family 2 (facilitated glucose transporter), member 1 | 4 |
| NM_053819 | Timp1 | Tissue inhibitor of metalloproteinase 1 | 4 |
| NM_001012125 | Loxl1 | Lysyl oxidase-like 1 | 4 |
| NM_012515 | Bzrp | Benzodiazepine receptor, peripheral | 3 |
| NM_198738 | Psat1 | Phosphoserine aminotransferase 1 | 3 |
| XM_001053002 /// XM_001053003 | RGD1359202 /// LOC299458 /// LOC366747 /// LOC678701 /// LOC678757 | Similar to immunoglobulin heavy chain 6 (Igh-6) /// similar to Ig H-chain V-region precursor /// similar to Ig heavy chain V region MC101 precursor /// hypothetical protein LOC678701 /// hypothetical protein LOC678757 | 3 |
| NM_021835 | Jun | Jun oncogene | 3 |
| NM_013002 | Pcp4 | Purkinje cell protein 4 | 3 |
| NM_017353 | Slc7a5 | Solute carrier family 7 (cationic amino acid transporter, y+ system), member 5 | 3 |
| NM_019904 | Lgals1 | Lectin, galactose binding, soluble 1 | 3 |
| NM_031140 | Vim | Vimentin | 3 |
| NM_001010964 | Klrb1a | Killer cell lectin-like receptor subfamily B, member 1A | 3 |
| NM_001004084 | RT1-Bb | RT1 class II, locus Bb | 3 |
| NM_021587 | Ltbp1 | Latent transforming growth factor beta binding protein 1 | 3 |
| NM_012935 | Cryab | Crystallin, alpha B | 3 |
| NM_017016 | Hdc | Histidine decarboxylase | 3 |
| NM_031569 /// NM_057098 /// XM_001055907 /// XM_345486 | Prpf6 /// Oprl1 /// Tcea2 | PRP6 pre-mrna processing factor 6 homolog (S. Cerevisiae)/// Opioid receptor-like 1 /// Transcription elongation factor A (SII), 2 | 3 |
| NM_139089 | Cxcl10 | Chemokine (C-X-C motif) ligand 10 | 3 |
| NM_001033073 | Defa-rs1 | Defensin alpha-related sequence 1 | 3 |
| NM_022542 | Rhob | Ras homolog gene family, member B | 3 |
| NM_016988 | Acp2 | Acid phosphatase 2, lysosomal | 3 |
| NM_012904 | Anxa1 | Annexin A1 | 3 |
| NM_206847 | Pfkp | Phosphofructokinase, platelet | 3 |
| NM_012600 | Me1 | Malic enzyme 1 | 3 |
| NM_012862 | Mgp | Matrix Gla protein | 3 |
| NM_031588 | Nrg1 | Neuregulin 1 | 3 |
| NM_022604 | Esm1 | Endothelial cell-specific molecule 1 | 3 |
| NM_012523 | Cd53 | CD53 antigen | 3 |
| NM_022298 | Tuba1 | Tubulin, alpha 1 | 3 |
| NM_019905 | Anxa2 | Annexin A2 | 3 |
| NM_053515 | Slc25a4 | Solute carrier family 25 (mitochondrial carrier; adenine nucleotide translocator), member 4 | 3 |
| NM_031987 | Crot | Carnitine O-octanoyltransferase | 3 |
| NM_053843 | Fcgr3 | Fc receptor, igg, low affinity III | 3 |
| XM_001060954 /// XM_001061024 /// XM_001061084 | Marcks /// LOC294446 /// LOC681252 | Myristoylated alanine rich protein kinase C substrate /// similar to Myristoylated alanine-rich C-kinase substrate (MARCKS) (ACAMP-81) /// similar to Myristoylated alanine-rich C-kinase substrate (MARCKS) (Protein kinase C substrate 80 kda protein) | 3 |
| NM_012722 | Eln | Elastin | 2 |
| XM_001055696 /// XM_001072336 | LOC680097 /// LOC684887 | Similar to germinal histone H4 gene /// similar to germinal histone H4 gene | 2 |
| NM_017099 | Kcnj8 | Potassium inwardly-rectifying channel, subfamily J, member 8 | 2 |
| NM_019283 | Slc3a2 | Solute carrier family 3 (activators of dibasic and neutral amino acid transport), member 2 | 2 |
| NM_001009628 /// NM_012696 | Kng1 /// MGC108747 | Kininogen 1 /// similar to alpha-1 major acute phase protein prepeptide | 2 |
| NM_001009920 | Yc2 | Glutathione S-transferase Yc2 subunit | 2 |
| NM_012541 | Cyp1a2 | Cytochrome P450, family 1, subfamily a, polypeptide 2 | 2 |
| NM_013215 | Akr7a3 | Aldo-keto reductase family 7, member A3 (aflatoxin aldehyde reductase) | 2 |
| NM_031114 | S100a10 | S100 calcium binding protein A10 (calpactin) | 2 |
| NM_019165 | Il18 | Interleukin 18 | 2 |
| NM_017066 | Ptn | Pleiotrophin | 2 |
| NM_198740 | Hla-dmb | Major histocompatibility complex, class II, DM beta | 2 |
| NM_012946 | Sparcl1 | SPARC-like 1 (mast9, hevin) | 2 |
| XM_001061982 /// XM_343564 | Col5a2 | Procollagen, type V, alpha 2 | 2 |
| NM_021261 | Tmsb10 | Thymosin, beta 10 | 2 |
| NM_024160 | Cyba | Cytochrome b-245, alpha polypeptide | 2 |
| NM_053360 | Sh3kbp1 | SH3-domain kinase binding protein 1 | 2 |
| NM_001008751 | Krt1-14 | Keratin complex 1, acidic, gene 14 | 2 |
| NM_017059 | Bax | Bcl2-associated X protein | 2 |
| NM_022525 | Gpx3 | Glutathione peroxidase 3 | 2 |
| NM_001002851 | Nenf | Neuron derived neurotrophic factor | 2 |
| XM_001054008 /// XM_215524 | Tpd52 | Tumor protein D52 | 2 |
| XM_001078048 /// XM_001079634 /// XM_001079646 /// XM_341942 | Plekha1 | Pleckstrin homology domain containing, family A (phosphoinositide binding specific) member 1 | 2 |
| NM_012713 | Prkcb1 | Protein kinase C, beta 1 | 2 |
| NM_022948 | Sfxn3 | Sideroflexin 3 | 2 |
| NM_199370 | Krt2-8 | Keratin complex 2, basic, gene 8 | 2 |
| XM_001068218 /// XM_574074 | RGD1564865 | Similar to 20-alpha-hydroxysteroid dehydrogenase | 2 |
| NM_017014 | Gstm1 | Glutathione S-transferase, mu 1 | 2 |
| NM_031740 | B4galt6 | UDP-Gal:betaglcnac beta 1,4-galactosyltransferase, polypeptide 6 | 2 |
| NM_130411 | Coro1a | Coronin, actin binding protein 1A | 2 |
| NM_133307 | Prkcd | Protein kinase C, delta | 2 |
| NM_019285 | Adcy4 | Adenylate cyclase 4 | 2 |
| NM_134449 | Prkcdbp | Protein kinase C, delta binding protein | 2 |
| NM_053976 | Krt1-18 | Keratin complex 1, acidic, gene 18 | 2 |
| NM_012738 | Apoa1 | Apolipoprotein A-I | 2* |
| NM_031789 | Nfe2l2 | Nuclear factor, erythroid derived 2, like 2 | 2 |
| XM_001067182 /// XM_215905 | Myl9 | Myosin, light polypeptide 9, regulatory | 2 |
| NM_053541 | Lrp3 | Low density lipoprotein receptor-related protein 3 | -2 |
| NM_012899 | Alad | Aminolevulinate, delta-, dehydratase | -2 |
| NM_030656 | Agxt | Alanine-glyoxylate aminotransferase | -2 |
| NM_130826 /// XM_343032 | Hadha | Hydroxyacyl-Coenzyme A dehydrogenase/3-ketoacyl-Coenzyme A thiolase/enoyl-Coenzyme A hydratase (trifunctional protein), alpha subunit | -2 |
| XM_001053949 /// XM_001054009 /// XM_001054056 /// XM_001054116 /// XM_001054179 /// XM_241375 | Narg1 | NMDA receptor-regulated gene 1 | -2 |
| NM_019249 | Ptprf | Protein tyrosine phosphatase, receptor type, F | -2 |
| NM_031507 | Egfr | Epidermal growth factor receptor | -2 |
| NM_134432 | Agt | Angiotensinogen (serpin peptidase inhibitor, clade A, member 8) | -2 |
| NM_031983 | Smarcd2 | SWI/SNF related, matrix associated, actin dependent regulator of chromatin, subfamily d, member 2 | -2 |
| XM_001056237 /// XM_341636 | Ces5 | Carboxylesterase 5 | -2 |
| NM_053598 | Nudt4 | Nudix (nucleoside diphosphate linked moiety X)-type motif 4 | -2 |
| NM_181371 | Gstk1 | Glutathione S-transferase kappa 1 | -2 |
| NM_012742 | Foxa1 | Forkhead box A1 | -2 |
| XM_001062585 /// XM_214583 | Hspa9a | Heat shock 70kda protein 9A | -2 |
| NM_183334 | Ldhal6b | Lactate dehydrogenase A-like 6B | -2 |
| NM_019303 | Cyp2f2 | Cytochrome P450, family 2, subfamily f, polypeptide 2 | -2 |
| NM_053770 | Argbp2 | Arg/Abl-interacting protein argbp2 | -2 |
| NM_017158 | Cyp2c7 | Cytochrome P450, family 2, subfamily c, polypeptide 7 | -2 |
| NM_053979 | Arl5a | ADP-ribosylation factor-like 5A | -2 |
| NM_053886 | Lman1 | Lectin, mannose-binding, 1 | -2 |
| NM_013029 | St8sia3 | ST8 alpha-N-acetyl-neuraminide alpha-2,8-sialyltransferase 3 | -2 |
| NM_031003 | Abat | 4-aminobutyrate aminotransferase | -2 |
| NM_013102 | Fkbp1a | FK506 binding protein 1a | -2 |
| NM_001012213 | Sfxn1 | Sideroflexin 1 | -2 |
| NM_022268 | Pygl | Liver glycogen phosphorylase | -2 |
| NM_019182 | Rnf4 | Ring finger protein 4 | -2 |
| NM_134365 | Atp5f1 | ATP synthase, H+ transporting, mitochondrial F0 complex, subunit b, isoform 1 | -2 |
| NM_012596 | Lepr | Leptin receptor | -2 |
| NM_022508 | Mthfd1 | Methylenetetrahydrofolate dehydrogenase (NADP+ dependent), methenyltetrahydrofolate cyclohydrolase, formyltetrahydrofolate synthase | -2 |
| NM_001007620 | Pdhb | Pyruvate dehydrogenase (lipoamide) beta | -2 |
| NM_030586 | Cyb5b | Cytochrome b5 type B | -2 |
| NM_019140 | Ptprd | Protein tyrosine phosphatase, receptor type, D | -2 |
| NM_013026 | Sdc1 | Syndecan 1 | -2* |
| NM_133558 | Cml1 | Camello-like 1 | -2 |
| NM_024391 | Hsd17b2 | Hydroxysteroid (17-beta) dehydrogenase 2 | -2 |
| NM_012578 | H1f0 | H1 histone family, member 0 | -2 |
| NM_023103 | Mug1 | Murinoglobulin 1 homolog (mouse) | -2 |
| XM_001072774 /// XM_340999 | Tfrc | Transferrin receptor | -2 |
| NM_053765 | Gne | Glucosamine | -2 |
| NM_173305 | Hsd17b9 | Hydroxysteroid (17-beta) dehydrogenase 9 | -2 |
| NM_031684 | Slc29a1 | Solute carrier family 29 (nucleoside transporters), member 1 | -2* |
| NM_017229 | Pde3b | Phosphodiesterase 3B | -2 |
| NM_001039610 | Ube2cbp | Ubiquitin-conjugating enzyme E2C binding protein | -2 |
| NM_031057 | Aldh6a1 | Aldehyde dehydrogenase family 6, subfamily A1 | -2 |
| NM_017193 | Aadat | Aminoadipate aminotransferase | -2 |
| NM_024386 | Hmgcl | 3-hydroxy-3-methylglutaryl-Coenzyme A lyase | -2 |
| NM_012886 | Timp3 | Tissue inhibitor of metalloproteinase 3 (Sorsby fundus dystrophy, pseudoinflammatory) | -2 |
| NM_017223 | Slc20a2 | Solute carrier family 20, member 2 | -2 |
| NM_080782 | Cdkn1a | Cyclin-dependent kinase inhibitor 1A | -2 |
| NM_017359 | Rab10 | RAB10, member RAS oncogene family | -2 |
| NM_031648 | Fxyd1 | FXYD domain-containing ion transport regulator 1 | -2 |
| XM_001059586 /// XM_001059704 /// XM_001059759 /// XM_001059813 /// XM_001059879 /// XM_001059950 /// XM_001060012 /// XM_001064131 /// XM_342854 | Nfib | Nuclear factor I/B | -2 |
| NM_144730 | Gata4 | GATA binding protein 4 | -2 |
| XM_001053322 /// XM_001058097 | Ube2cbp | Ubiquitin-conjugating enzyme E2C binding protein | -2 |
| NM_001014206 | RGD1309534 | Similar to RIKEN cdna 4931406C07 | -2 |
| NM_133315 | Slc40a1 | Solute carrier family 39 (iron-regulated transporter), member 1 | -2 |
| XR_008772 | RGD1565350 | Similar to Shb protein | -2 |
| NM_012716 | Slc16a1 | Solute carrier family 16 (monocarboxylic acid transporters), member 1 | -2 |
| NM_080886 | Sc4mol | Sterol-C4-methyl oxidase-like | -2 |
| NM_012780 | Arnt | Aryl hydrocarbon receptor nuclear translocator | -2 |
| NM_057133 | Nr0b2 | Nuclear receptor subfamily 0, group B, member 2 | -2 |
| NM_053596 | Ece1 | Endothelin converting enzyme 1 | -2 |
| NM_031517 | Met | Met proto-oncogene | -3 |
| NM_012737 | Apoa4 | Apolipoprotein A-IV | -3 |
| NM_001039549 | Ugt1a5 | UDP glycosyltransferase 1 family, polypeptide A5 | -3 |
| XM_001055943 /// XM_237286 | Tns | Tensin | -3 |
| NM_172335 | Gm2a | GM2 ganglioside activator protein | -3 |
| XM_001057445 /// XM_341542 | Cul2 | Cullin 2 | -3 |
| NM_145086 | Serbp1 | Serpine1 mrna binding protein 1 | -3 |
| NM_012969 | Irs1 | Insulin receptor substrate 1 | -3 |
| NM_139086 | Sycn | Syncollin | -3 |
| NM_022501 | Crip2 | Cysteine-rich protein 2 | -3 |
| NM_030872 | Pdk2 | Pyruvate dehydrogenase kinase, isoenzyme 2 | -3 |
| NM_138833 | Snrk | SNF related kinase | -3 |
| NM_133419 | Dkc1 | Dyskeratosis congenita 1, dyskerin | -3 |
| NM_031132 | Tgfbr2 | Transforming growth factor, beta receptor II | -3 |
| NM_057119 | Sfrs10 | Splicing factor, arginine/serine-rich 10 (transformer 2 homolog, Drosophila) | -3* |
| NM_001025271 | Sfpq | Splicing factor proline/glutamine rich (polypyrimidine tract binding protein associated) | -3 |
| NM_012545 | Ddc | Dopa decarboxylase | -3* |
| NM_016998 | Cpa1 | Carboxypeptidase A1 | -3 |
| NM_031589 | Slc37a4 | Solute carrier family 37 (glycerol-6-phosphate transporter), member 4 | -3 |
| NM_033095 | Crygd | Crystallin, gamma D | -3 |
| XM_001080576 /// XM_234277 | RGD1305721 | Similar to RIKEN cdna 2810055F11 | -3 |
| NM_001007235 | Itpr1 | Inositol 1,4,5-triphosphate receptor 1 | -3 |
| NM_017103 | Tceb3 | Transcription elongation factor B (SIII), polypeptide 3 | -3 |
| NM_031025 | Dlat | Dihydrolipoamide S-acetyltransferase (E2 component of pyruvate dehydrogenase complex) | -3 |
| NM_131906 | Slco1a4 | Solute carrier organic anion transporter family, member 1a4 | -3 |
| NM_001013124 | Ung | Uracil-DNA glycosylase | -3 |
| NM_012672 | Thrb | Thyroid hormone receptor beta | -3 |
| NM_013219 | Cadps | Ca2+-dependent secretion activator | -3 |
| NM_053923 | Pik3c2g | Phosphatidylinositol 3-kinase, C2 domain containing, gamma polypeptide | -3 |
| NM_022193 | Acaca | Acetyl-coenzyme A carboxylase alpha | -3 |
| XM_001055394 /// XM_218002 | Dact2 | Dapper homolog 2, antagonist of beta-catenin (xenopus) | -3 |
| NM_138911 | Stip1 | Stress-induced phosphoprotein 1 | -3 |
| NM_001014166 | Il33 | Interleukin 33 | -3 |
| NM_012683 | Ugt1a1 | UDP glycosyltransferase 1 family, polypeptide A1 | -3 |
| NM_019291 | Ca2 | Carbonic anhydrase 2 | -3 |
| NM_013078 | Otc | Ornithine transcarbamylase | -3 |
| NM_139192 | Scd1 | Stearoyl-Coenzyme A desaturase 1 | -3 |
| NM_017094 | Ghr | Growth hormone receptor | -3 |
| NM_172320 | Afm | Afamin | -3 |
| NM_024484 | Alas1 | Aminolevulinic acid synthase 1 | -3 |
| NM_053493 | Phyh2 | Phytanoyl-coa 2-hydroxylase 2 | -3 |
| NM_013198 | Maob | Monoamine oxidase B | -3 |
| NM_024486 | Acvr1 | Activin A receptor, type 1 | -3 |
| NM_177928 | Pbef1 | Pre-B-cell colony enhancing factor 1 | -3 |
| NM_017111 | Slc21a1 | Solute carrier family 21, member 1 | -3 |
| NM_001033860 /// NM_001033861 /// NM_001033862 /// NM_031755 | Ceacam1 | CEA-related cell adhesion molecule 1 | -3 |
| NM_001039031 | Dak | Dihydroxyacetone kinase 2 homolog (S. Cerevisiae) | -3 |
| NM_001014036 | Larp1b | La ribonucleoprotein domain family, member 1B | -3 |
| NM_001034028 /// NM_138867 | Hyou1 | Hypoxia up-regulated 1 | -3 |
| NM_019185 | Gata6 | GATA binding protein 6 | -3 |
| NM_012842 | Egf | Epidermal growth factor | -3 |
| NM_053881 | Ptprn | Protein tyrosine phosphatase, receptor type, N | -3 |
| NM_145091 | Pdp2 | Pyruvate dehydrogenase phosphatase isoenzyme 2 | -3 |
| NM_175766 | Cyp2j9 | Cytochrome P450, family 2, subfamily j, polypeptide 9 | -3 |
| NM_019143 | Fn1 | Fibronectin 1 | -4 |
| NM_019370 | Enpp3 | Ectonucleotide pyrophosphatase/phosphodiesterase 3 | -4 |
| NM_001037975 /// NM_023103 /// XM_001060284 /// XM_213006 | A1i3 /// Cpamd8 /// Mug1/// | Alpha-1-inhibitor III /// C3 and PZP-like, alpha-2-macroglobulin domain containing 8 /// Murinoglobulin 1 homolog (mouse)/// | -4 |
| XM_575338 | RGD1562323 | Similar to fatty acid translocase/CD36 | -4 |
| NM_031741 | Slc2a5 | Solute carrier family 2, member 5 | -4 |
| XM_001076104 /// XM_213943 | Mgst3 | Microsomal glutathione S-transferase 3 | -4 |
| XM_001067936 /// XM_233065 | RGD1561090 | Similar to protein tyrosine phosphatase, receptor type, D | -4 |
| NM_012988 | Nfia | Nuclear factor I/A | -4 |
| NM_053328 | Bhlhb2 | Basic helix-loop-helix domain containing, class B2 | -4 |
| NM_022180 | Hnf4a | Hepatocyte nuclear factor 4, alpha | -4 |
| NM_133545 | Ptpn21 | Protein tyrosine phosphatase, non-receptor type 21 | -4 |
| NM_012651 | Slc4a1 | Solute carrier family 4, member 1 | -4 |
| XM_001054250 | LOC679161 | Similar to transmembrane protein 64 | -4 |
| NM_031073 | Ntf3 | Neurotrophin 3 | -4 |
| NM_138904 | Gls2 | Glutaminase 2 (liver, mitochondrial) | -4 |
| XM_001072618 /// XM_001072656 /// XM_573819 | Tgfb1i4 /// LOC498545 | Transforming growth factor beta 1 induced transcript 4 /// similar to transforming growth factor beta 1 induced transcript 4 isoform 1 | -4 |
| NM_017332 | Fasn | Fatty acid synthase | -4* |
| NM_001013137 | Cxcl14 | Chemokine (C-X-C motif) ligand 14 | -4 |
| NM_138912 | Ppp1r3b | Protein phosphatase 1, regulatory (inhibitor) subunit 3B | -5 |
| NM_019292 | Ca3 | Carbonic anhydrase 3 | -5 |
| NM_138884 | Akr1d1 | Aldo-keto reductase family 1, member D1 | -5 |
| XM_001058099 /// XM_240417 | Mtmr7 | Myotubularin related protein 7 | -5 |
| NM_134329 | Adh7 | Alcohol dehydrogenase 7 (class IV), mu or sigma polypeptide | -5* |
| NM_031048 | Lifr | Leukemia inhibitory factor receptor | -5 |
| NM_001034111 /// NM_012630 | Prlr | Prolactin receptor | -5 |
| NM_022866 | Slc13a3 | Solute carrier family 13 (sodium-dependent dicarboxylate transporter), member 3 | -5 |
| NM_052798 | Zfp354a | Zinc finger protein 354A | -5 |
| NM_001025131 /// NM_012695 | Smp2a /// Sult2a2 | Rat senescence marker protein 2A gene, exons 1 and 2 /// sulfotransferase family 2A, dehydroepiandrosterone (DHEA)-preferring, member 2 | -5 |
| NM_017274 | Gpam | Glycerol-3-phosphate acyltransferase, mitochondrial | -6 |
| NM_022667 | Slco2a1 | Solute carrier organic anion transporter family, member 2a1 | -6 |
| NM_031120 | Ssr3 | Signal sequence receptor, gamma | -6 |
| NM_053962 | Sds | Serine dehydratase | -6 |
| NM_001013083 | Cpa2 | Carboxypeptidase A2 (pancreatic) | -6 |
| NM_031561 | Cd36 | Cd36 antigen | -6 |
| NM_012543 | Dbp | D site albumin promoter binding protein | -6 |
| NM_133295 | Ces3 | Carboxylesterase 3 | -6 |
| NM_016987 | Acly | ATP citrate lyase | -7 |
| NM_019287 | Apob | Apolipoprotein B | -9 |
| NM_021589 | Ntrk1 | Neurotrophic tyrosine kinase, receptor, type 1 | -10 |
| NM_017159 | Hal | Histidine ammonia lyase | -10 |
| NM_001000980 | Olr1366 | Olfactory receptor 1366 | -10 |
| NM_031135 | Klf10 | Kruppel-like factor 10 | -10 |
| NM_017070 | Srd5a1 | Steroid 5 alpha-reductase 1 | -11 |
| NM_022251 | Enpep | Glutamyl aminopeptidase | -12 |
| NM_144748 | Acsm2 | Acsm2 acyl-coa synthetase medium-chain family member 2 | -14 |
| XM_001054915 /// XM_343823 | Serpina7 | Serine (or cysteine) peptidase inhibitor, clade A (alpha-1 antipeptidase, antitrypsin), member 7 | -16 |
| NM_144750 | Aspg | Asparaginase homolog (S. Cerevisiae) | -28 |
| NM_053626 | Dao1 | D-amino acid oxidase 1 | -30 |
| NM_019278 | Resp18 | Regulated endocrine-specific protein 18 | -34 |
| XM_001067025 /// XM_001072862 /// XM_001081261 /// XM_001081265 /// XM_001081267 /// XM_213437 /// XM_224699 | Spop /// Tspan14 | Speckle-type POZ protein /// Transcribed locus /// Tetraspanin 14 | -38* |
| NM_147206 | Cyp3a13 | Cytochrome P450, family 3, subfamily a, polypeptide 13 | -606* |
| Shown above are a list of differentially expressed genes with a fold change ≥ 2-fold and a p-value < 0.05 as determined by t-test.  * Statistically significant with a p-value of < 0.05 following Benjamini-Hochberg FDR Correction. | | | |
