# Supplementary material for: Toxicogenomic analysis of exposure to TCDD, PCB126 and PCB153: identification of genomic biomarkers of exposure to AhR ligands
Source: BMC Genomics. 2010 Oct 19;11:583. doi: 10.1186/1471-2164-11-583 (PMC3091730; doi:10.1186/1471-2164-11-583)
Supplement: Additional file 7 — Microarray gene expression following 13 weeks of subchronic p.o. exposure to 1000 ng/kg/day PCB126 A list of the 371 genes differentially expressed genes following 13 weeks of subchronic exposure to 1000 ng/kg/day PCB126. A gene was considered to be differentially expressed if it displayed a gene expression fold change of 2 or greater. [file 1471-2164-11-583-S7.DOC]

| **Additional file 7: List of 371 genes differentially expressed genes following 13 weeks of subchronic p.o. exposure to 1000ng/kg/day PCB126** | | | |
| --- | --- | --- | --- |
| Transcript ID | Gene Symbol | Gene Name | Fold Change |
| NM_012540 | Cyp1a1 | Cytochrome P450, family 1, subfamily a, polypeptide 1 | 1140* |
| NM_012940 | Cyp1b1 | Cytochrome P450, family 1, subfamily b, polypeptide 1 | 519* |
| NM_173339 | Ceacam10 | CEA-related cell adhesion molecule 10 | 369* |
| NM_177426 | Gstm2 | Glutathione S-transferase, mu 2 | 174* |
| NM_012786 | Cox8h | Cytochrom c oxidase subunit VIII-H (heart/muscle) | 66* |
| NM_022245 | Cyb5 | Cytochrome b-5 | 59* |
| NM_019904 | Lgals1 | Lectin, galactose binding, soluble 1 | 24* |
| NM_012520 | Cat | Catalase | 23* |
| NM_017000 | Nqo1 | NAD(P)H dehydrogenase, quinone 1 | 21* |
| NM_053788 | Stx1a | Syntaxin 1A (brain) | 17* |
| XM_001081230 /// XM_213440 | Col1a1 | Procollagen, type 1, alpha 1 | 16 |
| NM_130407 | Ugt1a7 | UDP glycosyltransferase 1 family, polypeptide A7 | 14* |
| NM_001039691 /// NM_057105 | Ugt1a6 | UDP glycosyltransferase 1 family, polypeptide A6 | 11* |
| XM_001077432 /// XM_235518 | Ndufa6 | NADH dehydrogenase (ubiquinone) 1 alpha subcomplex, 6 (B14) | 11* |
| NM_031569 /// NM_057098 /// XM_001055907 /// XM_345486 | Prpf6 /// Oprl1 /// Tcea2 | PRP6 pre-mrna processing factor 6 homolog (S. Cerevisiae) /// Opioid receptor-like 1 /// Transcription elongation factor A (SII), 2 | 10 |
| NM_019360 | Cox6c | Cytochrome c oxidase, subunit vic | 9 |
| NM_024127 | Gadd45a | Growth arrest and DNA-damage-inducible 45 alpha | 9* |
| NM_198738 | Psat1 | Phosphoserine aminotransferase 1 | 8* |
| NM_012839 | Cycs | Cytochrome c, somatic | 8* |
| NM_012541 | Cyp1a2 | Cytochrome P450, family 1, subfamily a, polypeptide 2 | 7* |
| NM_057104 | Enpp2 | Ectonucleotide pyrophosphatase/phosphodiesterase 2 | 6* |
| XM_001054662 /// XM_216097 | Rpa3 | Replication protein A3 | 6* |
| NM_031572 | Cyp2c40 | Cytochrome P450, family 2, subfamily c, polypeptide 40 | 6* |
| NM_001024964 | Exoc3 | Exocyst complex component 3 | 6* |
| NM_017101 | Ppia | Peptidylprolyl isomerase A | 5* |
| NM_031972 | Aldh3a1 | Aldehyde dehydrogenase family 3, member A1 | 5* |
| NM_001010921 | LOC494499 | LOC494499 protein | 5* |
| NM_031130 | Nr2f1 | Nuclear receptor subfamily 2, group F, member 1 | 5 |
| NM_053698 | Cited2 | Cbp/p300-interacting transactivator, with Glu/Asp-rich carboxy-terminal domain, 2 | 5* |
| NM_021261 | Tmsb10 | Thymosin, beta 10 | 5* |
| NM_012941 | Cyp51 | Cytochrome P450, subfamily 51 | 4 |
| NM_017158 | Cyp2c7 | Cytochrome P450, family 2, subfamily c, polypeptide 7 | 4* |
| NM_017359 | Rab10 | RAB10, member RAS oncogene family | 4 |
| NM_001039610 | Ube2cbp | Ubiquitin-conjugating enzyme E2C binding protein | 4* |
| XM_001056178 /// XM_001072602 | LOC679398 /// LOC689938 | Similar to NADH dehydrogenase (ubiquinone) 1, subcomplex unknown, 1 /// similar to NADH dehydrogenase (ubiquinone) 1, subcomplex unknown, 1 | 4 |
| NM_175761 | Hspca | Heat shock protein 1, alpha | 4* |
| NM_012600 | Me1 | Malic enzyme 1 | 4* |
| NM_138547 | LOC191574 | 3-alpha-hydroxysteroid dehydrogenase | 4 |
| NM_001013933 | Ube2a | Ubiquitin-conjugating enzyme E2A, RAD6 homolog (S. Cerevisiae) | 4* |
| NM_012521 | S100g | S100 calcium binding protein G | 4 |
| NM_013105 /// NM_173144 | Cyp3a3 /// Cyp3a1 | Cytochrome P450, subfamily 3A, polypeptide 3 /// cytochrome P450, family 3, subfamily a, polypeptide 1 | 4* |
| NM_033095 | Crygd | Crystallin, gamma D | 3* |
| NM_001007264 | Ugt2b5 | UDP-glucuronosyltransferase 2 family, member 5 | 3* |
| NM_171994 | Cdc42 | Cell division cycle 42 homolog (S. Cerevisiae) | 3* |
| NM_031839 | Cyp2c23 | Cytochrome P450, family 2, subfamily c, polypeptide 23 | 3 |
| NM_017172 | Zfp36l1 | Zinc finger protein 36, C3H type-like 1 | 3* |
| NM_019630 | Gip | Gastric inhibitory polypeptide | 3 |
| NM_013123 | Il1r1 | Interleukin 1 receptor, type I | 3 |
| NM_175760 | Cyp4a14 | Cytochrome P450, family 4, subfamily a, polypeptide 14 | 3 |
| NM_032079 | Dnaja2 | Dnaj (Hsp40) homolog, subfamily A, member 2 | 3 |
| NM_022513 | Sult1b1 | Sulfotransferase family 1B, member 1 | 3* |
| NM_001014255 | Aph1a | Anterior pharynx defective 1a homolog (C. Elegans) | 3 |
| NM_022229 | Hspd1 | Heat shock protein 1 (chaperonin) | 3* |
| NM_022381 | Pcna | Proliferating cell nuclear antigen | 3 |
| NM_031533 | Ugt2b | UDP glycosyltransferase 2 family, polypeptide B | 3* |
| NM_012819 | Acadl | Acetyl-Coenzyme A dehydrogenase, long-chain | 3 |
| NM_031834 | Sult1a1 | Sulfotransferase family 1A, phenol-preferring, member 1 | 3* |
| NM_031058 | Msh2 | Muts homolog 2 (E. Coli) | 3* |
| XM_001079490 /// XM_215044 | Ndufab1 | NADH dehydrogenase (ubiquinone) 1, alpha/beta subcomplex, 1 | 3* |
| NM_001013932 | LOC298250 | Similar to hypothetical protein FLJ10986 | 3 |
| XM_001069131 /// XM_216929 | Ndufb9 | NADH dehydrogenase (ubiquinone) 1 beta subcomplex, 9 | 3* |
| NM_001004271 | Ugt2b4 | UDP glycosyltransferase 2 family, polypeptide B4 | 3 |
| NM_130428 | Sdha | Succinate dehydrogenase complex, subunit A, flavoprotein (Fp) | 3 |
| NM_172008 | Canx | Calnexin | 3* |
| NM_001000508 | Olr1687 | Olfactory receptor 1687 | 3 |
| NM_138826 | Mt1a | Metallothionein 1a | 3* |
| NM_198742 | Etfdh | Electron-transferring-flavoprotein dehydrogenase | 3* |
| XM_001054659 /// XM_001054730 /// XM_231184 | Dennd1a | DENN/MADD domain containing 1A | 3 |
| NM_017050 | Sod1 | Superoxide dismutase 1 | 3 |
| NM_001006957 | Wbscr1 | Williams-Beuren syndrome chromosome region 1 homolog (human) | 3* |
| NM_001011995 /// NM_001044270 /// NM_022298 | Tuba1 /// Tuba6 | Tubulin, alpha 1 /// tubulin, alpha 6 /// | 3 |
| NM_173295 | Udpgtr2 | Liver UDP-glucuronosyltransferase, phenobarbital-inducible form | 3* |
| NM_031643 | Map2k1 | Mitogen activated protein kinase kinase 1 | 3 |
| NM_053665 | Akap1 | A kinase (PRKA) anchor protein 1 | 3* |
| NM_053854 | Nat2 | N-Acetyltransferase-2 (arylamine N-acetyltransferase) | 3 |
| XR_006788 | LOC690089 | Hypothetical protein LOC690089 | 2* |
| NM_022175 | Pem | Placentae and embryos oncofetal gene | 2* |
| NM_144755 | Trib3 | Tribbles homolog 3 (Drosophila) | 2* |
| NM_182819 | Cox7b | Cytochrome c oxidase subunit viib | 2* |
| XM_001060627 /// XM_576251 | Zfp706 | Zinc finger protein 706 | 2 |
| NM_001015025 | Stk38 | Serine/threonine kinase 38 | 2 |
| NM_001035001 | Tmem69 | Transmembrane protein 69 | 2 |
| NM_001033987 /// NM_017246 | Map2k5 | Mitogen activated protein kinase kinase 5 | 2 |
| NM_130400 | Dhfr | Dihydrofolate reductase | 2 |
| NM_020101 | Centa2 | Centaurin, alpha 2 | 2 |
| NM_001013177 /// NM_133547 /// XM_576496 /// XM_576498 /// XR_007557 | Sult1c2 /// Sult1c2a /// RGD1559960 /// RGD1565421 | Sulfotransferase family, cytosolic, 1C, member 2 /// sulfotransferase family, cytosolic, 1C, member 2a /// similar to Sulfotransferase K1 (rsult1c2) /// similar to Sulfotransferase K2 (rsult1c2a) | 2 |
| NM_001025146 | Ndufs4 | NADH dehydrogenase (ubiquinone) Fe-S protein 4, 18kda (NADH-coenzyme Q reductase) | 2* |
| XM_001057695 /// XM_343588 | Ctdsp1 | CTD (carboxy-terminal domain, RNA polymerase II, polypeptide A) small phosphatase 1 | 2 |
| NM_022934 | Dnaja1 | Dnaj (Hsp40) homolog, subfamily A, member 1 | 2 |
| NM_031971 /// NM_212504 | Hspa1a /// Hspa1b | Heat shock 70kd protein 1A /// heat shock 70kd protein 1B | 2 |
| NM_024351 /// XM_001053720 /// XM_001055808 /// XM_001070485 /// XR_005876 /// XR_006039 /// XR_006475 /// XR_006759 /// XR_006848 /// XR_007318 /// XR_007979 /// XR_007999 /// XR_008434 | Hspa8 /// LOC295112 /// LOC298615 /// LOC498426 /// LOC499479 /// LOC679778 /// LOC680121 /// LOC689908 /// LOC689955 | Heat shock protein 8 /// similar to heat shock protein 8 /// hypothetical gene supported by NM_024351; M11942 /// similar to heat shock protein 8 /// similar to heat shock protein 8 /// similar to heat shock protein 8 /// similar to heat shock protein 8 /// similar to heat shock protein 8 /// similar to heat shock protein 8 | 2 |
| NM_012695 | Smp2a | Rat senescence marker protein 2A gene, exons 1 and 2 | 2 |
| NM_012583 | Hprt | Hypoxanthine guanine phosphoribosyl transferase | 2 |
| NM_130405 | Khdrbs1 | KH domain containing, RNA binding, signal transduction associated 1 | 2 |
| NM_212510 | MGC72957 | Similar to 60S ribosomal protein l18a | 2 |
| NM_031138 | Ube2b | Ubiquitin-conjugating enzyme E2B, RAD6 homolog (S. Cerevisiae) | 2* |
| XM_001065243 | LOC683283 | Similar to S-adenosylmethionine synthetase isoform type-2 (Methionine adenosyltransferase 2) (adomet synthetase 2) (Methionine adenosyltransferase II) (MAT-II) | 2 |
| XM_001062488 /// XM_001070713 | LOC682651 /// LOC689415 | Similar to Metallothionein-2 (MT-2) (Metallothionein-II) (MT-II) /// similar to Metallothionein-2 (MT-2) (Metallothionein-II) (MT-II) | 2 |
| NM_199403 | Dd25 | Hypothetical protein Dd25 | 2 |
| NM_031789 | Nfe2l2 | Nuclear factor, erythroid derived 2, like 2 | 2 |
| NM_001034163 | Blmh | Bleomycin hydrolase | 2 |
| NM_001008312 | Serinc3 | Serine incorporator 3 | 2 |
| XM_001055374 /// XM_001079531 /// XM_344862 | Znf324 /// LOC691759 | Zinc finger protein 324 /// Hypothetical protein LOC691759 | 2 |
| NM_012507 | Atp1b2 | Atpase, Na+/K+ transporting, beta 2 polypeptide | 2 |
| NM_053604 | Mgat2 | Mannoside acetylglucosaminyltransferase 2 | 2 |
| NM_031140 | Vim | Vimentin | 2 |
| NM_017284 | Psmb2 | Proteasome (prosome, macropain) subunit, beta type 2 | 2 |
| NM_017347 | Mapk3 | Mitogen activated protein kinase 3 | 2 |
| NM_031127 | Suox | Sulfite oxidase | -2 |
| NM_031551 | Idh3g | Isocitrate dehydrogenase 3 (NAD), gamma | -2 |
| NM_019259 | C1qbp | Complement component 1, q subcomponent binding protein | -2* |
| XM_001064851 /// XM_001065579 /// XM_001065640 /// XM_213898 | Ivns1abp | Influenza virus NS1A binding protein | -2* |
| XM_001072618 /// XM_001072656 /// XM_573819 | Tgfb1i4 /// LOC498545 | Transforming growth factor beta 1 induced transcript 4 /// similar to transforming growth factor beta 1 induced transcript 4 isoform 1 | -2 |
| NM_001013124 | Ung | Uracil-DNA glycosylase | -2 |
| NM_017290 | Atp2a2 | Atpase, Ca++ transporting, cardiac muscle, slow twitch 2 | -2 |
| NM_013003 | Pemt | Phosphatidylethanolamine N-methyltransferase | -2* |
| NM_021745 | Nr1h4 | Nuclear receptor subfamily 1, group H, member 4 | -2 |
| XM_001062585 /// XM_214583 | Hspa9a | Heat shock 70kda protein 9A | -2 |
| XM_001054505 /// XM_340973 | Ttc3 | Tetratricopeptide repeat domain 3 | -2 |
| XM_001080190 /// XM_239269 | Ankfy1 | Ankyrin repeat and FYVE domain containing 1 | -2 |
| XM_341141 | Bat2d1 | BAT2 domain containing 1 | -2* |
| NM_053596 | Ece1 | Endothelin converting enzyme 1 | -2* |
| XM_001058539 /// XM_233250 | C8b | Complement component 8, beta polypeptide | -2 |
| NM_001006981 | Dlst | Dihydrolipoamide S-succinyltransferase (E2 component of 2-oxo-glutarate complex) | -2 |
| NM_022952 | Ap2s1 | Adaptor-related protein complex 2, sigma 1 subunit | -2 |
| NM_019238 | Fdft1 | Farnesyl diphosphate farnesyl transferase 1 | -2 |
| NM_017072 | Cps1 | Carbamoyl-phosphate synthetase 1, mitochondrial | -2 |
| NM_080767 | Psmb8 | Proteosome (prosome, macropain) subunit, beta type 8 | -2 |
| NM_012930 | Cpt2 | Carnitine palmitoyltransferase 2 | -2* |
| NM_138904 | Gls2 | Glutaminase 2 (liver, mitochondrial) | -2* |
| NM_031720 | Dio2 | Deiodinase, iodothyronine, type II | -2* |
| NM_021577 /// NM_031330 | Asl /// Hnrpab | Argininosuccinate lyase /// heterogeneous nuclear ribonucleoprotein A/B | -2 |
| NM_030987 | Gnb1 | Guanine nucleotide binding protein, beta 1 | -2* |
| NM_031777 | Usf1 | Upstream transcription factor 1 | -2 |
| NM_053568 | Pcyt2 | Phosphate cytidylyltransferase 2, ethanolamine | -2* |
| NM_012603 | Myc | Myelocytomatosis viral oncogene homolog (avian) | -2 |
| XR_005443 /// XR_009642 /// XR_009643 /// XR_009644 | LOC501546 | Hypothetical protein LOC501546 | -2 |
| NM_031981 | Nsfl1c | NSFL1 (p97) cofactor (p47) | -2 |
| NM_134334 | Ctsd | Cathepsin D | -2 |
| XM_342524 | Plcb1 | Phospholipase C, beta 1 | -2* |
| NM_001007704 | Galm | Galactose mutarotase | -2 |
| NM_012891 | Acadvl | Acyl-Coenzyme A dehydrogenase, very long chain | -2* |
| XM_001058156 /// XM_341239 | Lrpap1 | Low density lipoprotein receptor-related protein associated protein 1 | -2 |
| NM_031049 | Lss | Lanosterol synthase | -2 |
| NM_001039031 | Dak | Dihydroxyacetone kinase 2 homolog (S. Cerevisiae) | -2 |
| NM_053969 | Gps1 | G protein pathway suppressor 1 | -2 |
| XM_001081842 /// XM_573235 | Fnsk | Similar to fructosamine-3-kinase | -2 |
| NM_012554 | Eno1 | Enolase 1, alpha | -2* |
| NM_031756 | Ggcx | Gamma-glutamyl carboxylase | -2* |
| NM_053537 | Slc22a7 | Solute carrier family 22 (organic anion transporter), member 7 | -2* |
| NM_053895 | Frag1 | FGF receptor activating protein 1 | -2* |
| NM_017097 | Ctsc | Cathepsin C | -2 |
| NM_031047 | Jup | Junction plakoglobin | -2* |
| NM_031561 /// XM_001061445 /// XM_001064109 /// XM_001068996 /// XM_001069037 /// XM_575338 /// XM_575339 | Cd36 /// RGD1562323 /// RGD1565355 /// LOC683013 /// LOC685953 | Cd36 antigen /// similar to fatty acid translocase/CD36 /// similar to fatty acid translocase/CD36 /// similar to CD36 antigen /// similar to CD36 antigen | -2 |
| NM_031626 | Nr1h2 | Nuclear receptor subfamily 1, group H, member 2 | -2 |
| NM_175754 | Agrn | Agrin | -2 |
| NM_080886 | Sc4mol | Sterol-C4-methyl oxidase-like | -2 |
| NM_012716 | Slc16a1 | Solute carrier family 16 (monocarboxylic acid transporters), member 1 | -2 |
| XM_001055723 /// XM_001073455 /// XM_214967 | Bclaf1 | BCL2-associated transcription factor 1 | -2* |
| NM_031589 | Slc37a4 | Solute carrier family 37 (glycerol-6-phosphate transporter), member 4 | -2* |
| NM_031333 | Cdh2 | Cadherin 2 | -2 |
| NM_013159 | Ide | Insulin degrading enzyme | -2 |
| NM_031978 | Psmd1 | Proteasome (prosome, macropain) 26S subunit, non-atpase, 1 | -2 |
| NM_057137 | Ebp | Phenylalkylamine Ca2+ antagonist (emopamil) binding protein | -2* |
| XM_001062965 /// XM_343439 | Bckdhb | Branched chain keto acid dehydrogenase E1, beta polypeptide | -2 |
| XM_001055798 /// XM_001055858 /// XM_001055911 /// XM_001055967 /// XM_001056031 /// XM_001056087 /// XM_001056156 /// XM_001056214 /// XM_001056279 /// XM_343513 | Aplp2 | Amyloid beta (A4) precursor-like protein 2 | -2* |
| XM_001075371 /// XM_345669 | RGD1309138 | Similar to hypothetical protein MGC9912 | -2 |
| NM_001013109 | Dapk2 | Death-associated kinase 2 | -2* |
| NM_053589 | Rab14 | RAB14, member RAS oncogene family | -2* |
| NM_013029 | St8sia3 | ST8 alpha-N-acetyl-neuraminide alpha-2,8-sialyltransferase 3 | -2 |
| NM_013221 | Hbp1 | High mobility group box transcription factor 1 | -2 |
| NM_022398 | Slc25a11 | Solute carrier family 25 (mitochondrial carrier; oxoglutarate carrier), member 11 | -2* |
| NM_052798 | Zfp354a | Zinc finger protein 354A | -2 |
| NM_032617 | Rab11b | RAB11B, member RAS oncogene family | -2 |
| XM_001058645 /// XM_227203 | Dhx36 | DEAH (Asp-Glu-Ala-His) box polypeptide 36 | -2 |
| NM_133591 | Rph3al | Rabphilin 3A-like (without C2 domains) | -2 |
| XM_001058886 /// XM_001058950 /// XM_233529 | Zmym4 /// LOC681811 | Zinc finger, MYM-type 4 /// similar to zinc finger protein 262 | -2* |
| NM_031087 | Psen2 | Presenilin 2 | -2* |
| NM_031975 | Ptms | Parathymosin | -2* |
| NM_053887 | Map3k1 | Mitogen activated protein kinase kinase kinase 1 | -2 |
| XM_001055358 /// XM_001065009 | LOC680021 /// LOC683694 | Similar to glyoxylate reductase/hydroxypyruvate reductase /// similar to glyoxylate reductase/hydroxypyruvate reductase | -2 |
| NM_133623 | Slc6a13 | Solute carrier family 6 (neurotransmitter transporter, GABA), member 13 | -2 |
| NM_024484 | Alas1 | Aminolevulinic acid synthase 1 | -2* |
| NM_178866 | Igf1 | Insulin-like growth factor 1 | -2 |
| NM_019286 | Adh1 | Alcohol dehydrogenase 1 (class I) | -2* |
| XM_001072973 /// XM_001073009 /// XM_001073040 | LOC686590 | Similar to IQ motif and Sec7 domain 1 | -2 |
| NM_017005 | Fh1 | Fumarate hydratase 1 | -2 |
| NM_017084 | Gnmt | Glycine N-methyltransferase | -2 |
| NM_080585 | Napa | N-ethylmaleimide sensitive fusion protein attachment protein alpha | -2* |
| NM_031776 | Gda | Guanine deaminase | -2* |
| NM_031851 | Phb | Prohibitin | -2* |
| NM_013016 | Ptpns1 | Protein tyrosine phosphatase, non-receptor type substrate 1 | -2 |
| NM_133315 | Slc40a1 | Solute carrier family 39 (iron-regulated transporter), member 1 | -2* |
| XM_001064355 /// XM_342068 | Fam160b1 | Family with sequence similarity 160, member B1 | -2 |
| NM_017148 | Csrp1 | Cysteine and glycine-rich protein 1 | -2* |
| NM_022508 | Mthfd1 | Methylenetetrahydrofolate dehydrogenase (NADP+ dependent), methenyltetrahydrofolate cyclohydrolase, formyltetrahydrofolate synthase | -2* |
| NM_001014036 | Larp1b | La ribonucleoprotein domain family, member 1B | -2 |
| NM_031035 | Gnai2 | Guanine nucleotide binding protein, alpha inhibiting 2 | -2 |
| NM_012723 | Pigr | Polymeric immunoglobulin receptor | -2 |
| NM_017255 | P2ry2 | Purinergic receptor P2Y, G-protein coupled 2 | -2* |
| NM_022694 | Snd1 | Staphylococcal nuclease domain containing 1 | -2 |
| NM_001014195 | Tmem214 | Transmembrane protein 214 | -2 |
| XM_575828 | RGD1562988 | Similar to EHM2 | -2 |
| XM_001053124 /// XM_341495 | Sptlc1 | Serine palmitoyltransferase, long chain base subunit 1 | -2 |
| NM_022667 | Slco2a1 | Solute carrier organic anion transporter family, member 2a1 | -2 |
| XM_001075680 /// XM_213329 | Srebf1 | Sterol regulatory element binding factor 1 | -2 |
| NM_024132 | Faah | Fatty acid amide hydrolase | -2* |
| NM_133541 | Gtf3c1 | General transcription factor III C 1 | -2 |
| NM_012500 | Apeh | Acylpeptide hydrolase | -2 |
| NM_024391 | Hsd17b2 | Hydroxysteroid (17-beta) dehydrogenase 2 | -2* |
| NM_022180 | Hnf4a | Hepatocyte nuclear factor 4, alpha | -2 |
| NM_012649 | Sdc4 | Syndecan 4 | -2 |
| NM_022588 | Mta1 | Metastasis associated 1 | -2* |
| NM_012866 | Nfyc | Nuclear transcription factor-Y gamma | -2 |
| NM_001012047 | Btd | Biotinidase | -2 |
| NM_012504 | Atp1a1 | Atpase, Na+/K+ transporting, alpha 1 polypeptide | -2* |
| XM_001064051 /// XM_575876 | RGD1564074 | Similar to novel protein | -2* |
| NM_021593 | Kmo | Kynurenine 3-monooxygenase (kynurenine 3-hydroxylase) | -2* |
| NM_173101 | Myo1e | Myosin IE | -2* |
| NM_053493 | Phyh2 | Phytanoyl-coa 2-hydroxylase 2 | -2 |
| XM_001056237 /// XM_341636 | Ces5 | Carboxylesterase 5 | -2* |
| NM_022947 | Clpb | Clpb caseinolytic peptidase B homolog (E. Coli) | -2 |
| XM_001069036 /// XM_342465 | Harbi1l | Harbinger transposase derived 1-like | -2* |
| NM_001013137 | Cxcl14 | Chemokine (C-X-C motif) ligand 14 | -2 |
| NM_017335 | Slc6a12 | Solute carrier family 6 (neurotransmitter transporter, betaine/GABA), member 12 | -2 |
| NM_001017493 | LOC498265 | Similar to hypothetical protein FLJ10706 | -2 |
| NM_001012152 /// NM_001034021 | Tbc1d14 | TBC1 domain family, member 14 | -2 |
| NM_017307 | Slc25a1 | Solute carrier family 25, member 1 | -2* |
| NM_053623 | Acsl4 | Acyl-coa synthetase long-chain family member 4 | -2* |
| NM_001012504 /// XM_001058855 | Set /// LOC681893 | SET nuclear oncogene /// similar to SET protein (Phosphatase 2A inhibitor I2PP2A) (I-2PP2A) (Template-activating factor I) (TAF-I) (Liver regeneration-related protein LRRGR00002) | -2* |
| NM_012656 | Sparc | Secreted acidic cysteine rich glycoprotein | -2* |
| NM_053904 | Oplah | 5-oxoprolinase (ATP-hydrolysing) | -2 |
| NM_001031640 | Eif3s9 | Eukaryotic translation initiation factor 3, subunit 9 (eta) | -2 |
| NM_053553 | Syngr2 | Synaptogyrin 2 | -2 |
| XM_001058034 /// XM_001058095 /// XM_001060277 | Atp6v1a1 /// Atp6v1a1 | Atpase, H transporting, lysosomal V1 subunit A /// atpase, H transporting, lysosomal V1 subunit A | -2* |
| NM_001008280 | Lrrc59 | Leucine rich repeat containing 59 | -2* |
| XM_001059462 /// XM_001059526 /// XM_001059578 /// XM_573923 | RGD1561067 | Similar to RNA binding protein gene with multiple splicing | -2 |
| NM_017088 | Gdi1 | Guanosine diphosphate dissociation inhibitor 1 | -2 |
| NM_019379 | Vdp | Vesicle docking protein | -2 |
| XM_001061438 /// XM_001068584 /// XM_001068834 /// XM_574504 | Nup98 | Nucleoporin 98 | -2* |
| NM_022951 | Ppp1r10 | Protein phosphatase 1, regulatory subunit 10 | -2* |
| NM_017211 | Glg1 | Golgi apparatus protein 1 | -2 |
| NM_012895 | Adk | Adenosine kinase | -3 |
| NM_139329 | Hsd3b7 | Hydroxy-delta-5-steroid dehydrogenase, 3 beta- and steroid delta-isomerase 7 | -3* |
| NM_145724 /// XM_001081477 /// XM_573196 | Zfp99 /// Ccdc56 | Zinc finger protein 99 /// coiled-coil domain containing 56 | -3 |
| NM_017048 | Slc4a2 | Solute carrier family 4, member 2 | -3* |
| NM_139113 | Nr2f6 | Nuclear receptor subfamily 2, group F, member 6 | -3 |
| NM_031135 | Klf10 | Kruppel-like factor 10 | -3* |
| NM_012545 | Ddc | Dopa decarboxylase | -3 |
| NM_145092 | Tor1aip1 | Torsin A interacting protein 1 | -3 |
| NM_012789 | Dpp4 | Dipeptidylpeptidase 4 | -3* |
| NM_017033 | Pgm1 | Phosphoglucomutase 1 | -3* |
| NM_019291 | Ca2 | Carbonic anhydrase 2 | -3 |
| NM_022287 | Slc26a1 | Solute carrier family 26 (sulfate transporter), member 1 | -3* |
| NM_001037979 | Adipor2 | Adiponectin receptor 2 | -3 |
| NM_033230 | Akt1 | Thymoma viral proto-oncogene 1 | -3* |
| NM_031683 | Smc1l1 | Structural maintenance of chromosomes 1 like 1 (S. Cerevisiae) | -3 |
| NM_031700 | Cldn3 | Claudin 3 | -3* |
| NM_133395 | Tpo1 | Developmentally regulated protein TPO1 | -3 |
| XM_001058993 /// XM_343764 | Maoa | Monoamine oxidase A | -3 |
| NM_012998 | P4hb | Prolyl 4-hydroxylase, beta polypeptide | -3 |
| XM_001056725 /// XM_238213 | NIPBL /// LOC681423 | Nipped-B homolog (Drosophila) /// similar to delangin isoform A | -3* |
| XM_001060536 /// XM_001071999 | Slc12a7 | Solute carrier family 12 (potassium/chloride transporters), member 7 | -3 |
| XM_001078236 /// XM_343856 | Litaf | LPS-induced TN factor | -3 |
| XM_001053539 /// XM_001053609 /// XM_225404 | Cdc2l5 | Cell division cycle 2-like 5 (cholinesterase-related cell division controller) | -3* |
| NM_031795 | Ugcg | UDP-glucose ceramide glucosyltransferase | -3 |
| NM_024130 | Dctn1 | Dynactin 1 | -3 |
| NM_012907 | Apobec1 | Apolipoprotein B editing complex 1 | -3 |
| NM_022518 | Arf1 | ADP-ribosylation factor 1 | -3 |
| NM_031002 | Inpp4a | Inositol polyphosphate-4-phosphatase, type 1 | -3 |
| NM_138863 | Ltb4dh | Leukotriene B4 12-hydroxydehydrogenase | -3* |
| NM_053743 | Cdc37 | Cell division cycle 37 homolog (S. Cerevisiae) | -3* |
| NM_001012213 | Sfxn1 | Sideroflexin 1 | -3* |
| XM_001068594 /// XM_574314 | Plg | Plasminogen | -3 |
| XM_001058099 /// XM_240417 | Mtmr7 | Myotubularin related protein 7 | -3* |
| XM_001059684 /// XM_217310 | Slc25a23 | Solute carrier family 25 (mitochondrial carrier; phosphate carrier), member 23 | -3* |
| NM_057140 | Ppp2r1a | Protein phosphatase 2 (formerly 2A), regulatory subunit A (PR 65), alpha isoform | -3 |
| NM_080787 | Dgka | Diacylglycerol kinase, alpha | -3 |
| NM_199256 | Sec61a1 | Sec61 alpha 1 subunit (S. Cerevisiae) | -3 |
| NM_053923 | Pik3c2g | Phosphatidylinositol 3-kinase, C2 domain containing, gamma polypeptide | -3 |
| NM_017094 | Ghr | Growth hormone receptor | -3 |
| NM_023098 | Nup62 | Nucleoporin 62 | -3 |
| NM_030861 | Mgat1 | Mannoside acetylglucosaminyltransferase 1 | -3 |
| XM_001072449 /// XM_215303 | RT1-S3 | RT1 class Ib, locus S3 | -3 |
| NM_013177 | Got2 | Glutamate oxaloacetate transaminase 2, mitochondrial | -3 |
| NM_021750 | Csad | Cysteine sulfinic acid decarboxylase | -3 |
| NM_138896 | Pja2 | Praja 2, RING-H2 motif containing | -3 |
| NM_144748 | Acsm2 | Acyl-coa synthetase medium-chain family member 2 | -3 |
| NM_031684 | Slc29a1 | Solute carrier family 29 (nucleoside transporters), member 1 | -3* |
| XM_001065075 /// XM_341241 | Mxd4 | Max dimerization protein 4 | -3 |
| NM_019303 | Cyp2f2 | Cytochrome P450, family 2, subfamily f, polypeptide 2 | -3 |
| NM_031614 | Txnrd1 | Thioredoxin reductase 1 | -3 |
| XR_009418 | LOC688018 | Similar to SH3-domain binding protein 3 | -3* |
| NM_053625 | Gfm | G elongation factor | -3* |
| NM_013026 | Sdc1 | Syndecan 1 | -3* |
| NM_017340 | Acox1 | Acyl-Coenzyme A oxidase 1, palmitoyl | -3 |
| NM_013219 | Cadps | Ca2+-dependent secretion activator | -3 |
| NM_031008 | Ap2a2 | Adaptor protein complex AP-2, alpha 2 subunit | -3* |
| NM_031063 | Mvk | Mevalonate kinase | -3* |
| NM_199404 | Man2b1 | Mannosidase 2, alpha B1 | -3* |
| NM_053576 | Prdx6 | Peroxiredoxin 6 | -3* |
| NM_017159 | Hal | Histidine ammonia lyase | -3 |
| NM_021744 | Cd14 | CD14 antigen | -3* |
| NM_031707 | Homer1 | Homer homolog 1 (Drosophila) | -3 |
| NM_147206 | Cyp3a13 | Cytochrome P450, family 3, subfamily a, polypeptide 13 | -3 |
| NM_053463 | Nucb1 | Nucleobindin 1 | -3 |
| NM_139256 | Man2c1 | Mannosidase, alpha, class 2C, member 1 | -3 |
| XM_001080123 /// XM_220047 | Tnks2 | Tankyrase, TRF1-interacting ankyrin-related ADP-ribose polymerase 2 | -3* |
| NM_019140 | Ptprd | Protein tyrosine phosphatase, receptor type, D | -3* |
| NM_001008766 | Wdr39 | WD repeat domain 39 | -3 |
| NM_012524 | Cebpa | CCAAT/enhancer binding protein (C/EBP), alpha | -3* |
| NM_199112 | RGD735175 | Hypothetical protein MGC:72616 | -3 |
| NM_001008325 | Tmem218 | Transmembrane protein 218 | -3 |
| NM_013134 | Hmgcr | 3-hydroxy-3-methylglutaryl-Coenzyme A reductase | -3 |
| NM_022297 | Ddah1 | Dimethylarginine dimethylaminohydrolase 1 | -3* |
| NM_031012 | Anpep | Alanyl (membrane) aminopeptidase | -3* |
| XM_001077885 /// XM_220541 | Ulk2 | Unc-51 like kinase 2 (C. Elegans) | -3 |
| NM_031615 | Zfp148 | Zinc finger protein 148 | -3* |
| NM_133295 | Ces3 | Carboxylesterase 3 | -3* |
| NM_198780 | Pck1 | Phosphoenolpyruvate carboxykinase 1 | -3* |
| XM_001053672 /// XM_343427 | Nedd4a | Neural precursor cell expressed, developmentally down-regulated gene 4A | -3 |
| NM_016990 | Add1 | Adducin 1 (alpha) | -3* |
| XM_001076104 /// XM_213943 | Mgst3 | Microsomal glutathione S-transferase 3 | -3 |
| NM_019220 | Aes | Amino-terminal enhancer of split | -3 |
| NM_138549 | Gpsn2 | Glycoprotein, synaptic 2 | -3* |
| XM_001054915 /// XM_343823 | Serpina7 | Serine (or cysteine) peptidase inhibitor, clade A (alpha-1 antipeptidase, antitrypsin), member 7 | -3 |
| NM_057133 | Nr0b2 | Nuclear receptor subfamily 0, group B, member 2 | -3* |
| NM_017269 | Ptprj | Protein tyrosine phosphatase, receptor type, J | -3* |
| NM_019318 | Maf | V-maf musculoaponeurotic fibrosarcoma oncogene homolog (avian) | -3* |
| NM_022690 | Ube2g1 | Ubiquitin-conjugating enzyme E2G 1 (UBC7 homolog, C. Elegans) | -4 |
| NM_022860 | B4galnt1 | Beta-1,4-N-acetyl-galactosaminyl transferase 1 | -4* |
| NM_017245 | Eef2 | Eukaryotic translation elongation factor 2 | -4 |
| NM_153308 | Grina | Glutamate receptor, ionotropic, N-methyl D-asparate-associated protein 1 (glutamate binding) | -4* |
| NM_144750 | Aspg | Asparaginase homolog (S. Cerevisiae) | -4* |
| NM_001014166 | Il33 | Interleukin 33 | -4* |
| NM_053329 | Igfals | Insulin-like growth factor binding protein, acid labile subunit | -4* |
| NM_024139 | Chp | Calcium binding protein p22 | -4 |
| XM_001066753 /// XM_001066809 /// XM_344570 | Fgfr4 | Fibroblast growth factor receptor 4 | -4 |
| XM_001081576 /// XM_573211 | Ern1 | Endoplasmic reticulum to nucleus signaling 1 | -4* |
| NM_022592 | Tkt | Transketolase | -4 |
| NM_053881 | Ptprn | Protein tyrosine phosphatase, receptor type, N | -4 |
| NM_022399 | Calr | Calreticulin | -4* |
| NM_012988 | Nfia | Nuclear factor I/A | -4* |
| NM_022865 | Gphn | Gephyrin | -4 |
| NM_138840 | Tgoln2 | Trans-golgi network protein 2 | -4 |
| NM_012836 | Cpd | Carboxypeptidase D | -4 |
| NM_017070 | Srd5a1 | Steroid 5 alpha-reductase 1 | -4 |
| NM_031604 | Atp6v0a1 | Atpase, H+ transporting, lysosomal V0 subunit A1 | -4* |
| NM_138877 | Cyb5r3 | Cytochrome b5 reductase 3 | -4* |
| NM_019370 | Enpp3 | Ectonucleotide pyrophosphatase/phosphodiesterase 3 | -4* |
| XM_001059586 /// XM_001059704 /// XM_001059759 /// XM_001059813 /// XM_001059879 /// XM_001059950 /// XM_001060012 /// XM_001064131 /// XM_342854 | Nfib | Nuclear factor I/B | -4* |
| XM_001064459 /// XM_232351 | Cops7a | COP9 (constitutive photomorphogenic) homolog, subunit 7a (Arabidopsis thaliana) | -4* |
| NM_017235 | Hsd17b7 | Hydroxysteroid (17-beta) dehydrogenase 7 | -4 |
| NM_145091 | Pdp2 | Pyruvate dehydrogenase phosphatase isoenzyme 2 | -4 |
| NM_012637 | Ptpn1 | Protein tyrosine phosphatase, non-receptor type 1 | -4 |
| NM_031056 | Mmp14 | Matrix metallopeptidase 14 (membrane-inserted) | -4 |
| NM_147136 | LOC257642 | Rrna promoter binding protein | -5 |
| XM_001057484 /// XM_225706 | Atp9b | Atpase, class II, type 9B | -5 |
| NM_017206 | Slc6a6 | Solute carrier family 6 (neurotransmitter transporter, taurine), member 6 | -5 |
| NM_001008725 | Il6st | Interleukin 6 signal transducer | -5* |
| NM_031576 | Por | P450 (cytochrome) oxidoreductase | -5 |
| NM_012683 | Ugt1a1 | UDP glycosyltransferase 1 family, polypeptide A1 | -5* |
| NM_030872 | Pdk2 | Pyruvate dehydrogenase kinase, isoenzyme 2 | -5* |
| NM_133418 | Slc25a10 | Solute carrier family 25 (mitochondrial carrier; dicarboxylate transporter), member 10 | -5* |
| NM_022866 | Slc13a3 | Solute carrier family 13 (sodium-dependent dicarboxylate transporter), member 3 | -5 |
| NM_145770 | Acox2 | Acyl-Coenzyme A oxidase 2, branched chain | -6 |
| NM_001039549 | Ugt1a5 | UDP glycosyltransferase 1 family, polypeptide A5 | -6 |
| XM_001071608 /// XM_213849 | Nfix | Nuclear factor I/X | -6* |
| NM_017332 | Fasn | Fatty acid synthase | -6 |
| NM_001014264 | Igha /// LOC366772 | Immunoglobulin heavy chain (alpha polypeptide) /// similar to immunoglobulin heavy chain | -7* |
| NM_019278 | Resp18 | Regulated endocrine-specific protein 18 | -7 |
| Shown above are a list of differentially expressed genes with a fold change ≥ 2-fold and a p-value < 0.05 as determined by t-test.  * Statistically significant with a p-value of < 0.05 following Benjamini-Hochberg FDR Correction. | | | |
