# Supplementary material for: Toxicogenomic analysis of exposure to TCDD, PCB126 and PCB153: identification of genomic biomarkers of exposure to AhR ligands
Source: BMC Genomics. 2010 Oct 19;11:583. doi: 10.1186/1471-2164-11-583 (PMC3091730; doi:10.1186/1471-2164-11-583)
Supplement: Additional file 8 — Microarray gene expression following 13 weeks of subchronic p.o. exposure to 1000 μg/kg/day PCB153 A list of the 39 genes differentially expressed following 13 weeks of subchronic exposure to 1000 μg/kg/day PCB153. A gene was considered to be differentially expressed if it displayed a gene expression fold change of 2 or greater. [file 1471-2164-11-583-S8.DOC]

| **Additional file 8: List of 39 genes differentially expressed following 13 weeks of subchronic p.o. exposure to 1000µg/kg/day PCB153** | | | |
| --- | --- | --- | --- |
| Transcript ID | Gene Symbol | Gene Name | Fold change |
| NM_017156 | Cyp2b15 | Cytochrome P450, family 2, subfamily b, polypeptide 15 | 136 |
| XM_001062335 /// XM_001070917 /// XM_001070953 /// XM_341808 | Cyp2b2 | Cytochrome P450, family 2, subfamily b, polypeptide 2 | 92 |
| NM_031841 | Scd2 | Stearoyl-Coenzyme A desaturase 2 | 22 |
| NM_133586 | Ces2 | Carboxylesterase 2 (intestine, liver) | 8 |
| XM_001067025 /// XM_001072862 /// XM_001081261 /// XM_001081265 /// XM_001081267 /// XM_213437 /// XM_224699 | Spop /// Tspan14 | Speckle-type POZ protein /// Transcribed locus /// Tetraspanin 14 | 6 |
| XM_214551 | Cidea | Cell death-inducing DNA fragmentation factor, alpha subunit-like effector A | 4 |
| XM_001062874 /// XM_001077570 | Cyp2b15 /// LOC687222 | Cytochrome P450, family 2, subfamily b, polypeptide 15 /// similar to Cytochrome P450 2B12 (CYPIIB12) | 4 |
| NM_001007724 | Dcakd | Dephospho-coa kinase domain containing | 3 |
| XM_001057445 /// XM_341542 | Cul2 | Cullin 2 | 3 |
| XM_001057103 | LOC680422 | Hypothetical protein LOC680422 | 3 |
| NM_017098 | Fabp6 | Fatty acid binding protein 6, ileal (gastrotropin) | 3 |
| NM_198738 | Psat1 | Phosphoserine aminotransferase 1 | 3 |
| NM_024363 | Hrmt1l2 | Heterogeneous nuclear ribonucleoproteins methyltransferase-like 2 (S. Cerevisiae) | 3 |
| NM_031051 /// XM_001054317 /// XM_001074688 /// XM_001077597 /// XM_001081626 /// XM_212679 /// XM_575621 /// XR_006037 /// XR_007991 | Mif /// RGD1565459 /// LOC365286 /// RGD1560513 /// LOC679748 /// LOC686548 | Macrophage migration inhibitory factor /// similar to ribosomal protein L10a /// similar to Macrophage migration inhibitory factor (MIF) (Phenylpyruvate tautomerase) (Glycosylation-inhibiting factor) (GIF) (Delayed early response protein 6) (DER6) /// similar to macrophage migration inhibitory factor /// similar to Macrophage migration inhibitory factor (MIF) (Phenylpyruvate tautomerase) (Glycosylation-inhibiting factor) (GIF) (Delayed early response protein 6) (DER6) /// similar to Macrophage migration inhibitory factor (MIF) (Phenylpyruvate tautomerase) (Glycosylation-inhibiting factor) (GIF) (Delayed early response protein 6) (DER6) | 2 |
| NM_001014255 | Aph1a | Anterior pharynx defective 1a homolog (C. Elegans) | 2 |
| NM_013105 /// NM_173144 | Cyp3a3 /// Cyp3a1 | Cytochrome P450, subfamily 3A, polypeptide 3 /// cytochrome P450, family 3, subfamily a, polypeptide 1 | 2 |
| NM_001013932 | Fggy | FGGY carbohydrate kinase domain containing | 2 |
| NM_001025730 | Fbxw5 | F-box and WD-40 domain protein 5 | 2 |
| NM_001008312 | Serinc3 | Serine incorporator 3 | 2 |
| NM_031983 | Smarcd2 | SWI/SNF related, matrix associated, actin dependent regulator of chromatin, subfamily d, member 2 | 2 |
| XM_001062488 /// XM_001070713 | LOC682651 /// LOC689415 | Similar to Metallothionein-2 (MT-2) (Metallothionein-II) (MT-II) /// similar to Metallothionein-2 (MT-2) (Metallothionein-II) (MT-II) | 2 |
| NM_012742 | Foxa1 | Forkhead box A1 | 2 |
| NM_013197 | Alas2 | Aminolevulinic acid synthase 2 | 2 |
| NM_199102 | MGC72560 | Unknown (protein for MGC:72560) | 2 |
| NM_001033653 /// NM_057103 | Akap12 | A kinase (PRKA) anchor protein (gravin) 12 | 2 |
| XM_001053271 /// XM_343548 | Phf3 | PHD finger protein 3 | 2 |
| NM_134465 | Tslpr | Thymic stromal-derived lymphopoietin, receptor | 2 |
| NM_012668 | Tat | Tyrosine aminotransferase | -2 |
| NM_017073 | Glul | Glutamate-ammonia ligase (glutamine synthase) | -2 |
| NM_030586 | Cyb5b | Cytochrome b5 type B | -2 |
| XM_001056725 /// XM_238213 | NIPBL /// LOC681423 | Nipped-B homolog (Drosophila) /// similar to delangin isoform A | -2 |
| NM_175838 | Eef1a1 | Eukaryotic translation elongation factor 1 alpha 1 | -2 |
| NM_001014071 | Errfi1 | ERBB receptor feedback inhibitor 1 | -2 |
| NM_175761 | Hspca | Heat shock protein 1, alpha | -2 |
| NM_031720 | Dio2 | Deiodinase, iodothyronine, type II | -2 |
| NM_001044260 /// XM_001058170 /// XM_001062318 /// XM_001062437 /// XM_001069481 /// XM_341405 /// XR_005450 /// XR_009609 | LOC361117 /// LOC361325 /// LOC362308 /// LOC366304 /// LOC498826 /// LOC498973 /// LOC499531 /// LOC501173 /// LOC501449 /// LOC685107 /// LOC689117 | Similar to Rb1-inducible coiled coil protein 1 /// similar to RIKEN cdna 2410116I05 /// similar to ORF1 /// similar to LRRGT00126 /// LRRGT00165 /// nucleic acid binding protein /// nucleic acid binding protein /// hypothetical protein LOC501173 /// hypothetical protein LOC501449 /// hypothetical protein LOC685107 /// hypothetical protein LOC689117 | -2 |
| NM_053769 | Dusp1 | Dual specificity phosphatase 1 | -3 |
| NM_198780 | Pck1 | Phosphoenolpyruvate carboxykinase 1 | -3 |
| NM_001001504 | Gtf2ird1 | General transcription factor II I repeat domain-containing 1 | -4 |
| Shown above are a list of differentially expressed genes with a fold change ≥ 2-fold and a p-value < 0.05 as determined by t-test.  * Statistically significant with a p-value of < 0.05 following Benjamini-Hochberg FDR Correction. | | | |
