# Supplementary material for: Toxicogenomic analysis of exposure to TCDD, PCB126 and PCB153: identification of genomic biomarkers of exposure to AhR ligands
Source: BMC Genomics. 2010 Oct 19;11:583. doi: 10.1186/1471-2164-11-583 (PMC3091730; doi:10.1186/1471-2164-11-583)
Supplement: Additional file 9 — Microarray gene expression following 52 weeks of chronic p.o. exposure to 1000 μg/kg/day PCB153 A list of the 47 genes differentially expressed following 52 weeks of chronic exposure to 1000 μg/kg/day PCB153. A gene was considered to be differentially expressed if it displayed a gene expression fold change of 2 or greater. [file 1471-2164-11-583-S9.DOC]

| **Additional file 9: List of 47 genes differentially expressed following 52 weeks of chronic p.o. exposure to 1000µg/kg/day PCB153** | | | |
| --- | --- | --- | --- |
| Transcript ID | Gene Symbol | Gene Name | Fold Change |
| NM_017156 | Cyp2b15 | Cytochrome P450, family 2, subfamily b, polypeptide 15 | 104* |
| NM_031841 | Scd2 | Stearoyl-Coenzyme A desaturase 2 | 90 |
| XM_001062335 /// XM_001070917 /// XM_001070953 /// XM_341808 | Cyp2b2 | Cytochrome P450, family 2, subfamily b, polypeptide 2 | 23 |
| NM_031732 | Sult1c1 | Sulfotransferase family, cytosolic, 1C, member 1 | 22 |
| NM_133586 | Ces2 | Carboxylesterase 2 (intestine, liver) | 8 |
| NM_145782 | Cyp3a18 | Cytochrome P450, 3a18 | 6 |
| NM_080581 | Abcc3 | ATP-binding cassette, sub-family C (CFTR/MRP), member 3 | 5 |
| NM_138836 | Prss8 | Protease, serine, 8 (prostasin) | 5 |
| XM_001062874 /// XM_001077570 | Cyp2b15 /// LOC687222 | Cytochrome P450, family 2, subfamily b, polypeptide 15 /// similar to Cytochrome P450 2B12 (CYPIIB12) | 5 |
| NM_001014264 | Igha /// LOC366772 | Immunoglobulin heavy chain (alpha polypeptide) /// similar to immunoglobulin heavy chain | 5 |
| NM_012881 | Spp1 | Secreted phosphoprotein 1 | 4 |
| NM_031530 | Ccl2 | Chemokine (C-C motif) ligand 2 | 4 |
| NM_017149 | Meox2 | Mesenchyme homeobox 2 | 3 |
| NM_012551 | Egr1 | Early growth response 1 | 3 |
| NM_198738 | Psat1 | Phosphoserine aminotransferase 1 | 3 |
| NM_012598 | Lpl | Lipoprotein lipase | 3 |
| NM_013105 /// NM_173144 | Cyp3a3 /// Cyp3a1 | Cytochrome P450, subfamily 3A, polypeptide 3 /// cytochrome P450, family 3, subfamily a, polypeptide 1 | 3 |
| NM_001007264 | Ugt2b5 | UDP-glucuronosyltransferase 2 family, member 5 | 2 |
| NM_031795 | Ugcg | UDP-glucose ceramide glucosyltransferase | 2 |
| NM_017166 | Stmn1 | Stathmin 1 | 2 |
| NM_138541 | Tacstd1 | Tumor-associated calcium signal transducer 1 | 2 |
| NM_024127 | Gadd45a | Growth arrest and DNA-damage-inducible 45 alpha | 2 |
| NM_024484 | Alas1 | Aminolevulinic acid synthase 1 | 2 |
| NM_022407 | Aldh1a1 | Aldehyde dehydrogenase family 1, member A1 | 2 |
| NM_001004084 | RT1-Bb | RT1 class II, locus Bb | 2 |
| NM_019140 | Ptprd | Protein tyrosine phosphatase, receptor type, D | 2 |
| NM_001040019 | RGD1562373 | Similar to 3-ketoacyl-coa thiolase B, peroxisomal precursor (Beta-ketothiolase B) | 2 |
| XM_214551 | Cidea | Cell death-inducing DNA fragmentation factor, alpha subunit-like effector A | 2 |
| NM_177425 | Csrp2 | Cysteine and glycine-rich protein 2 | 2 |
| XM_001062386 /// XM_001074876 /// XM_214250 /// XM_217114 | Cldn10 /// RGD1311744 | Ig active lambda2-like chain mrna, 3' end /// Clone lumo27.1 immunoglobulin lambda light chain /// Claudin 10 /// Similar to RIKEN cdna 5830475I06 | 2 |
| XM_001058806 /// XM_343227 | Nipal2 | NIPA-like domain containing 2 | 2 |
| NM_012940 | Cyp1b1 | Cytochrome P450, family 1, subfamily b, polypeptide 1 | 2 |
| XM_001067936 /// XM_233065 | RGD1561090 | Similar to protein tyrosine phosphatase, receptor type, D | -2 |
| NM_001012197 | Tra1 | Tumor rejection antigen gp96 | -2 |
| NM_022180 | Hnf4a | Hepatocyte nuclear factor 4, alpha | -2 |
| NM_031649 | Klrg1 | Killer cell lectin-like receptor subfamily G, member 1 | -2 |
| NM_013177 | Got2 | Glutamate oxaloacetate transaminase 2, mitochondrial | -2 |
| NM_012988 | Nfia | Nuclear factor I/A | -2 |
| NM_138833 | Snrk | SNF related kinase | -2 |
| NM_173102 | Tubb5 | Tubulin, beta 5 | -2 |
| NM_019143 | Fn1 | Fibronectin 1 | -2 |
| NM_031048 | Lifr | Leukemia inhibitory factor receptor | -3 |
| NM_021589 | Ntrk1 | Neurotrophic tyrosine kinase, receptor, type 1 | -3 |
| NM_017073 | Glul | Glutamate-ammonia ligase (glutamine synthase) | -3 |
| NM_031120 | Ssr3 | Signal sequence receptor, gamma | -3 |
| NM_001000980 | Olr1366 | Olfactory receptor 1366 | -4 |
| NM_012786 | Cox8h | Cytochrom c oxidase subunit VIII-H (heart/muscle) | -46 |
| Shown above are a list of differentially expressed genes with a fold change ≥ 2-fold and a p-value < 0.05 as determined by t-test.  * Statistically significant with a p-value of < 0.05 following Benjamini-Hochberg FDR Correction. | | | |
